# Stability selection for LASSO with weights based on AUC

**DOI:** 10.1038/s41598-023-32517-4

**Published:** 2023-03-30

**Authors:** Yonghan Kwon, Kyunghwa Han, Young Joo Suh, Inkyung Jung

**Affiliations:** 1 Department of Biostatistics and Computing, Yonsei University Graduate School, 50-1 Yonsei-ro, Seodaemun-gu, Seoul, Republic of Korea; 2 Department of Radiology, Research Institute of Radiological Science, and Center for Clinical Imaging Data Science, Yonsei University College of Medicine, 50-1 Yonsei-ro, Seodaemun-gu, Seoul, Republic of Korea; 3 Division of Biostatistics, Department of Biomedical Systems Informatics, Yonsei University College of Medicine, 50-1 Yonsei-ro, Seodaemun-gu, Seoul, Republic of Korea

**Keywords:** Computational science, Software, Statistics

## Abstract

Stability selection is a variable selection algorithm based on resampling a dataset. Based on stability selection, we propose weighted stability selection to select variables by weighing them using the area under the receiver operating characteristic curve (AUC) from additional modelling. Through an extensive simulation study, we evaluated the performance of the proposed method in terms of the true positive rate (TPR), positive predictive value (PPV), and stability of variable selection. We also assessed the predictive ability of the method using a validation set. The proposed method performed similarly to stability selection in terms of the TPR, PPV, and stability. The AUC of the model fitted on the validation set with the selected variables of the proposed method was consistently higher in specific scenarios. Moreover, when applied to radiomics and speech signal datasets, the proposed method had a higher AUC with fewer variables selected. A major advantage of the proposed method is that it enables researchers to select variables intuitively using relatively simple parameter settings.

## Introduction

Variable selection methods to determine the best subset that explains the relationship between the explanatory variable and the response variables have been extensively studied, especially in the high dimensionality of $p>>n,$ where $p$ denotes the number of variables of covariates and $n$ denotes the number of samples. Many methods have been proposed, namely, the classic best-subset selection, forward- and backward-stepwise selection^1^, and the relatively recent, least absolute shrinkage and selection operator (LASSO)^2^. Meinshausen and Bühlmann^3^ introduced stability selection, which can be widely combined with other variable selection procedures.

The basic idea of stability selection is to define a frequently selected variable as a stable variable by repeating the variable selection method several times on subsampled data. Stability selection is unique as it can adjust the upper bound of the per-family error rate (PFER), which is the expected value of the number of falsely selected variables. Shah and Samworth^4^ later combined this with complementary pair subsampling to further mitigate the assumption of the error bound in the PFER. Stability selection is a groundbreaking concept in variable selection and is currently widely used in genome research, the most representative example of a field concerned with high-dimensional data^5–8^.

Based on stability selection^4^ with logistic LASSO regression, this study proposes a method for selecting variables by weighing them through the area under the receiver operating characteristic (ROC) curve from additional modelling rather than treating all variables equally. A ROC curve is a graphical representation that depicts how a binary classifier system’s diagnostic performance changes when the discrimination threshold changes. Plotting the true positive rate against the false positive rate at various threshold levels yields an ROC curve. The area under ROC curve (AUC) is a metric that provides a comprehensive evaluation of the model's prediction performance, ranging from 0.5 to 1 with a value closer to 1 indicating higher performance of the model’s prediction^9^. Considering that many machine learning problems aim to predict the binary outcomes, we thought that adding the AUC information into stability selection process would lead to better prediction performance.

Similar to stability selection, the variable selection model of the proposed method is repeated several times to calculate the frequency of the selected variable. The AUC, calculated with the models fitted by the selected variables, is reflected as a weight to enable variable selection to increase the prediction ability. We evaluated the true positive rate (TPR), positive predictive value (PPV), and stability of variable selection of the proposed method by comparing the stability selection through an extensive simulation study. We assessed the predictive ability of the proposed method using the AUC on a validation set. In addition, we used radiomics data on aortic valve calcium^10^ and speech signal data on Parkinson's disease^11^ to demonstrate the application of the proposed method to real data.

## Background

### Logistic LASSO regression

Logistic regression is widely used to estimate the probability of a specific binary class or event. Generally, logistic regression is expressed as follows:

Given $n$ collections of predictor and response pairs ${\left\{\left({{\varvec{x}}}_{{\varvec{i}}},{y}_{i}\right)\right\}}_{i=1}^{n}$ with a binary response ${y}_{i}\in \left\{\mathrm{0,1}\right\}$ and a predictor vector ${{\varvec{x}}}_{i}=\left({x}_{i1},\dots ,{x}_{ip}\right)$, a linear logistic regression model is written as:

1 $$\mathrm{log}\frac{P\left({y}_{i}=1|{{\varvec{x}}}_{i}\right)}{P\left({y}_{i}=0|{{\varvec{x}}}_{i}\right)}={\beta }_{0}+{{\varvec{\beta}}}^{{\varvec{T}}}{{\varvec{x}}}_{{\varvec{i}}}$$

where, ${\beta }_{0}$ is an intercept term and ${\varvec{\beta}}=\left({\beta }_{1},\dots ,{\beta }_{p}\right)$ is the $p\times 1$ vector of regression coefficients.

However, logistic regression cannot be applied without modification in high-dimensional settings^12^. Tibshirani^2^ proposed LASSO, which minimizes the negative log-likelihood function under the condition that the sum of the absolute values of the regression coefficients is smaller than a given constant $\lambda$, a regularisation parameter that controls the sparsity of the estimator. Depending on the constraint of the LASSO estimator, the regression coefficient is reduced and some are shrunk to be zero. The LASSO estimator was proposed as a new estimator that simultaneously includes the features of ridge regression and subset selection of explanatory variables. Given model (1), the logistic LASSO estimator ${\widehat{\beta }}_{0}^{\lambda },{\widehat{{\varvec{\beta}}}}^{\lambda }$ is defined as follows:

$$\underset{{\beta }_{0},{\varvec{\beta}}}{\mathrm{argmin}}\sum_{i=1}^{n}\left\{{-y}_{i}\left({\beta }_{0}+{{\varvec{\beta}}}^{T}{{\varvec{x}}}_{{\varvec{i}}}\right)+\mathrm{log}\left(1+{e}^{{\beta }_{0}+{{\varvec{\beta}}}^{{\varvec{T}}}{{\varvec{x}}}_{{\varvec{i}}}}\right)\right\} subject \,\,to \sum_{j=1}^{p}\left|{\beta }_{j}\right|\le \lambda .$$

Cross-validation is used to select the optimal value of $\lambda$. ${\lambda }_{min}$ is the value of $\lambda$ that corresponds to the minimum mean cross-validated error, whereas ${\lambda }_{1se}$ corresponds to the highest level of regularization, while ensuring that the cross-validated error remains within one standard error of the minimum. The ${\lambda }_{1se}$ value gives a model that is less complex than the model produced by ${\lambda }_{min}$. It strikes a balance between model complexity and prediction accuracy, reduces the risk of overfitting, and produces a model with fewer non-zero coefficients, which improves interpretability^1^.

### Stability selection by Meinshausen and Bühlmann

Meinshausen and Bühlmann^3^ proposed the idea of stability selection through a frequency-based stability path rather than a regularisation path in LASSO. The regularisation path is the given coefficient value of each variable across all the regularisation parameters, whereas stability paths are the probabilities of each variable being chosen after resampling the data. The unique feature of stability selection is that the PFER $E(V)$, which is the expected value of the false positive number $V$, can be adjusted using the variable selection method.

Let $S$ represent the set of signal variables. ${\widehat{S}}_{n}$ refers to the set of variables selected by a statistical procedure with $n$ observations. Meinshausen and Bühlmann^3^ demonstrate that PFER are bounded by:

2 $$E\left(V\right)\le \frac{{q}^{2}}{\left(2{\pi }_{thr}-1\right)p},$$

where, $q$ is the number of (unique) selected variables (or groups of variables, depending on the model) in each subsample and ${\pi }_{thr}$ is defined as the threshold for the relative frequency of selected variables. Meinshausen and Bühlmann^3^ recommend that $q$ should be large enough to theoretically pick all $S$. ${\pi }_{thr}$ is recommended to be set between $\left(0.6,0.9\right)$. In general, any value between $\left(\mathrm{0.5,1}\right)$ is possibly acceptable (such that, for a variable to be regarded as stable, the variable must be selected in more than half of the fitted models).

Once the parameters are set, stability selection proceeds as follows:

1. For $$b=1$$ to $$B$$:
  - Pick a random subset of data with size $$\left\lfloor {n/2} \right\rfloor$$$$,$$ where $$\left\lfloor n \right\rfloor$$ is the largest integer $$\le n.$$
  - Fit a model in which as many variables as $q$.
2. Measure relative frequencies per variable:
  $$\hat{\pi }_{j} = \frac{1}{B}\mathop \sum \limits_{b = 1}^{B} {\mathbb{I}}_{{\left\{ {j \in \hat{S}_{{\left\lfloor {n/2} \right\rfloor ,b}} } \right\} }} ,j = 1, \ldots ,p,$$
  where $\hat{S}_{{\left\lfloor {n/2} \right\rfloor ,b}}$ denotes set of selected variables with $\left\lfloor {n/2} \right\rfloor$ observations over ${b}_{th}$ time.
3. Choose the variables that meet the following criteria and define them as stable variables:
  $${\widehat{S}}_{stable}=\left\{j:{\widehat{\pi }}_{j}\ge {\pi }_{thr}\right\}.$$

Two assumptions were required to apply the method above. First, all noise variables must have the same probability of being selected. Second, individual variable selection methods should be better than random guessing methods.

### Stability selection by Shah and Samworth

Shah and Samworth^4^ proposed a modified version of Meinshausen and Bühlmann’s^3^ stability selection. First, they used two subsamples as complementary pairs by randomly splitting sample B times in half and using both to measure the relative frequencies per variable:

$$\tilde{\pi }_{j} = \frac{1}{B}\mathop \sum \limits_{b = 1}^{B} {\mathbb{I}}_{{\left\{ {j \in \hat{S}^{1}_{{\left\lfloor {n/2} \right\rfloor ,b}} } \right\} }} {\mathbb{I}}_{{\left\{ {j \in \hat{S}^{2}_{{\left\lfloor {n/2} \right\rfloor ,b}} } \right\} ,}}$$

$\hat{S}^{1}_{{\left\lfloor {n/2} \right\rfloor ,b}}$ denotes a set of selected variables with $\left\lfloor {n/2} \right\rfloor$ observations at $b_{th}$ time. $\hat{S}^{2}_{{\left\lfloor {n/2} \right\rfloor ,b}}$ is the complementary pair of $\hat{S}^{1}_{{\left\lfloor {n/2} \right\rfloor ,b}}$, which uses the other $\left\lfloor {n/2} \right\rfloor$ observations that $\hat{S}^{1}_{{\left\lfloor {n/2} \right\rfloor ,b}}$ does not use. Second, they proposed three new error bounds that can be used even if the above two assumptions^3^ are not shown. This can be accomplished by controlling the modified bounds of the “expected number of selected variables with low selection probability” as expressed by $E\left(\left|{\widehat{S}}_{stable}\cap {L}_{\theta }\right|\right)$ rather than controlling the PFER. ${L}_{\theta }=\left\{j :{\widehat{\pi }}_{j}\le \theta \right\}$ represents the set of variables with a low probability of selection for $\hat{S}_{{\left\lfloor {n/2} \right\rfloor }}$. Usually, $\theta$ is defined $\frac{q}{p}$, which is the average rate of the selected variables. Three error bounds are suggested.

- None: Error bound without additional assumptions as proposed by Meinshausen and Bühlmann^3^.
- Unimodal: Error bound, assuming that ${\widetilde{\pi }}_{j}$ follows a unimodal probability distribution for all $j\in {L}_{\theta }$.
- r-concave: Error bound, assuming that ${\widetilde{\pi }}_{j}$ follows an r-concave probability distribution with $r=-\frac{1}{2}$ and, ${\widehat{\pi }}_{j}$ follows an r-concave probability distribution with $r=-\frac{1}{4}$ for all $j\in {L}_{\theta }$.

For a rigorous and thorough description of the error bounds, refer to Shah and Samworth^4^.

## Proposed method

We propose a new variable selection method called the weighted stability selection, which weighs the variable frequency based on the complementary pair stability selection presented by Shah and Samworth^4^.

**Figure 1:**
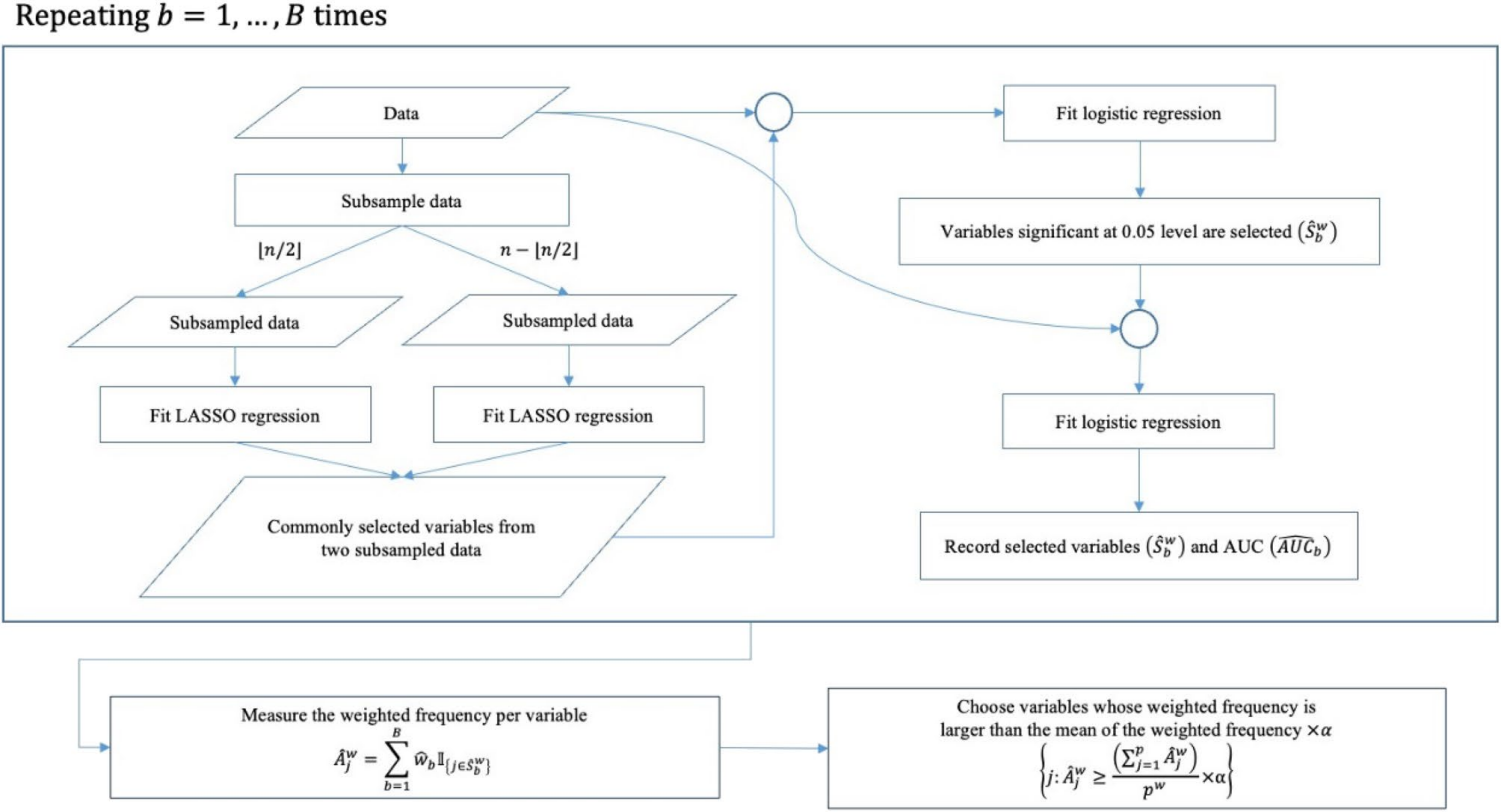
The whole process of weighted stability selection.

The detailed process is as follows:

1. For $$b=1$$ to $$B$$:
  - Divide a dataset randomly with sample size $n$ into two $\left\lfloor {n/2} \right\rfloor$ and $n - \left\lfloor {n/2} \right\rfloor$ non-overlapping size datasets. Fit a logistic regression with LASSO penalty to each of the two datasets and select the common variables selected from the two models.
  - Fit logistic regression with the variables selected in (a) to the original data.
  - In the model fitted in 1(b), only the variables significant at the 0.05 significance level were re-selected, denoted as the set ${\widehat{S}}_{b}^{w}$.
  - Fit logistic regression with the ${\widehat{S}}_{b}^{w}$ to the original data and calculate the AUC, denoted as ${\widehat{AUC}}_{b}$.
2. Measure the weighted frequency per variable denoted as ${\widehat{A}}_{j}^{w}=\sum_{b=1}^{B}{\widehat{w}}_{b}{\mathbb{I}}_{\left\{j\in {\widehat{S}}_{b}^{w}\right\}}$, where ${\widehat{w}}_{b}$ is the $b$ th rescaled $\left\{{\widehat{AUC}}_{1},\dots ,{\widehat{AUC}}_{B}\right\}$ using a min–max scaler.
3. Choose the variables that meet the following criteria:
  $$\left\{j:{\widehat{A}}_{j}^{w}\ge \frac{\left(\sum_{j=1}^{p}{\widehat{A}}_{j}^{w}\right)}{{p}^{w}}\times \alpha \right\},$$
  where ${p}^{w}$ is the number of variables selected at least once in step 1, and $\alpha$ is a parameter used to adjust the threshold for variable selection. The procedure for the proposed weighted stability selection is summarised in Fig. 1.

In step 1, we added steps (b) and (c) to re-select variables selected commonly from the two logistic regression models with LASSO penalty fitted to two subsamples. The reason is that we tried to make variables selection even more stable. We observed that skipping the steps could result in an excessive number of variables being selected, especially in high dimensional datasets, and variable selection can vary significantly.

When calculating the weighted frequency per variable using the AUC in step 2, the AUC values were rescaled into the range of 0 to 1. By rescaling the AUC values, it became easier to compare the importance of different variables and to identify the variables that are more important to the model's predictive performance. This step was essential to ensure that each variable was given a weight that reflected its contribution to the overall predictive importance of the model.

In Step 3, we used a criterion based on weighted frequencies to select variables. We selected variables whose weighted frequency was greater than the average weighted frequency multiplied by a parameter $\alpha$. The average weighted frequency was calculated only for variables that were selected at least once in step 1. We introduced parameter $\alpha$ in this step to adjust the threshold for variable selection based on their weighted frequency. One may choose an α value to select an appropriate number of variables. The recommended approach for optimizing α value is to start with an initial value of 0, gradually increase it by 0.1 units, and choose the value of α that results in the variables that fit the model with the highest AUC on the validation set.

## Simulation

### Simulation setting

We set the sample size ($n$) to 200 and 500, and the number of variables ($p$) to 500, 700, and 1000. The number of signal variables (${p}_{signal}$) was set to 10 and 20. When generating data through the linear predictor, only the ${\beta }_{j}$’s of the signal variables had a non-zero value from a uniform distribution of $U(0.5, 1.5)$ and $U(-\mathrm{3,3})$, and those of the remaining non-signal variables were set to zero. Additionally, we considered event prevalence of 10%, 30%, and 50%.

The dependent variables are generated through a binomial distribution that follows the probability of a logistic function with the following linear predictor:

$${y}_{i}\sim \mathrm{Binom}\left(\frac{\mathrm{exp}\left({{\varvec{x}}}_{i}^{T}{\varvec{\beta}}\right)}{1+\mathrm{exp}\left({{\varvec{x}}}_{i}^{T}{\varvec{\beta}}\right)}\right),$$

${{\varvec{x}}}_{i}$ are independently drawn from ${N}_{p}\left(0,\Sigma \right)$ which are considered two settings for the covariance matrix. The first is the case of an independent structure in which the covariance between all variables is zero: $\Sigma =I$ . The other is the case in which the covariance between all variables has a Toeplitz structure, with an interval of 0.9 magnitudes, ${\Sigma }_{kl}={0.9}^{\left|k-l\right|},k,l=1,\dots ,p$.

The stability selection^4^ is set with the following parameter combination settings: $\mathrm{PFER\,\, upper \,\,bound}\in \left\{\mathrm{1,2},\mathrm{5,10}\right\},$ $\mathrm{error\,\, bound \,\,assumption }\in \left\{r-concave,unimodal,none\right\}, {\pi }_{thr}\in \left\{\mathrm{0.6,0.75,0.9}\right\},$ and $B=50$ complementary pairs. Since we cannot know the number of essential variables $q$ ahead of time, we suggest providing the $\mathrm{PFER upper bound}$ and ${\pi }_{thr}$ first and then let $q$ be computed by (2). The weighted stability selection parameter $\alpha$ is set to $\left\{\mathrm{0.5,0.6,0.7,0.8,0.9,1.0,1.1,1.2,1.3,1.4,1.5}\right\}.$ As $\mathrm{\alpha }$ increases, the number of selected variables decreases, and vice versa.

We used TPR, PPV, several stability indicators, and AUC to show the results of each variable selection method. TPR is the proportion of the predicted signal variables to the actual signal variables. The PPV is the proportion of actual signal variables among the predicted signal variables. Stability indicators are defined as follows: ${\widehat{S}}_{d}\subseteq \{1,\dots ,p\}$ denotes the set of variables selected by the statistical procedure for the $d\mathrm{th}$ out of 50 simulation datasets. Two out of the 50 simulation data were paired to calculate stability. The two pairs are represented as ${d}_{u}$ and ${d}_{v}$, $u=1,\dots 50,v=1,\dots 50, u\ne v,$

- Jaccard similarity coefficient^13,14^:
  $$Jaccard\left({\widehat{S}}_{{d}_{u}},{\widehat{S}}_{{d}_{v}}\right)=\frac{\left|{\widehat{S}}_{{d}_{u}}\cap {\widehat{S}}_{{d}_{v}}\right|}{\left|{\widehat{S}}_{{d}_{u}}\cup {\widehat{S}}_{{d}_{v}}\right|}$$
- Otsuka–Ochiai coefficient^13,15^:
  $$Oc{\text{h}}iai\left({\widehat{S}}_{{d}_{u}},{\widehat{S}}_{{d}_{v}}\right)=\frac{\left|{\widehat{S}}_{{d}_{u}}\cap {\widehat{S}}_{{d}_{v}}\right|}{\sqrt{\left|{\widehat{S}}_{{d}_{u}}\right|\times \left|{\widehat{S}}_{{d}_{v}}\right|}}$$
- Sørensen–Dice coefficient^13,16^:
  $$Dice\left({\widehat{S}}_{{d}_{u}},{\widehat{S}}_{{d}_{v}}\right)=\frac{2\left|{\widehat{S}}_{{d}_{u}}\cap {\widehat{S}}_{{d}_{v}}\right|}{\left|{\widehat{S}}_{{d}_{u}}\right|+\left|{\widehat{S}}_{{d}_{v}}\right|}$$

We repeated each scenario 50 times and presented the results of TPR, PPV, several stability metrics (Jaccard similarity coefficient, Otsuka-Ochiai coefficient, and Sørensen-Dice coefficient), and AUC for all parameter cases of stability selection and weighted stability selection on a boxplot. To properly evaluate the AUC, we generated a validation set with the same settings as the scenario in which the variable selection was made. Therefore, we obtained 50 AUC values of the models fitted to the validation set using the variables selected by each variable selection method.

### Simulation result

Here we only presented the simulation results for four specific scenarios:

- Scenario 1. $n=500$, $p=1000,$ ${p}_{signal}=20$, ${\beta }_{j}$’s of the signal variables $\sim U(-\mathrm{3,3})$, event prevalence = 50%, and the covariance structure of $X =$ Toeplitz.
- Scenario 2. $n=500$, $p=1000,$ ${p}_{signal}=20$, ${\beta }_{j}$’s of the signal variables $\sim U(0.5,1.5)$, event prevalence = 50%, and the covariance structure of $X =$ Toeplitz.
- Scenario 3. $n=200$, $p=1000,$ ${p}_{signal}=20$, ${\beta }_{j}$’s of the signal variables $\sim U(-\mathrm{3,3})$, event prevalence = 50%, and the covariance structure of $X =$ Toeplitz.
- Scenario 4. $n=200$, $p=1000,$ ${p}_{signal}=20$, ${\beta }_{j}$’s of the signal variables $\sim U(0.5,1.5)$, event prevalence = 50%, and the covariance structure of $X =$ Toeplitz.

**Figure 2:**
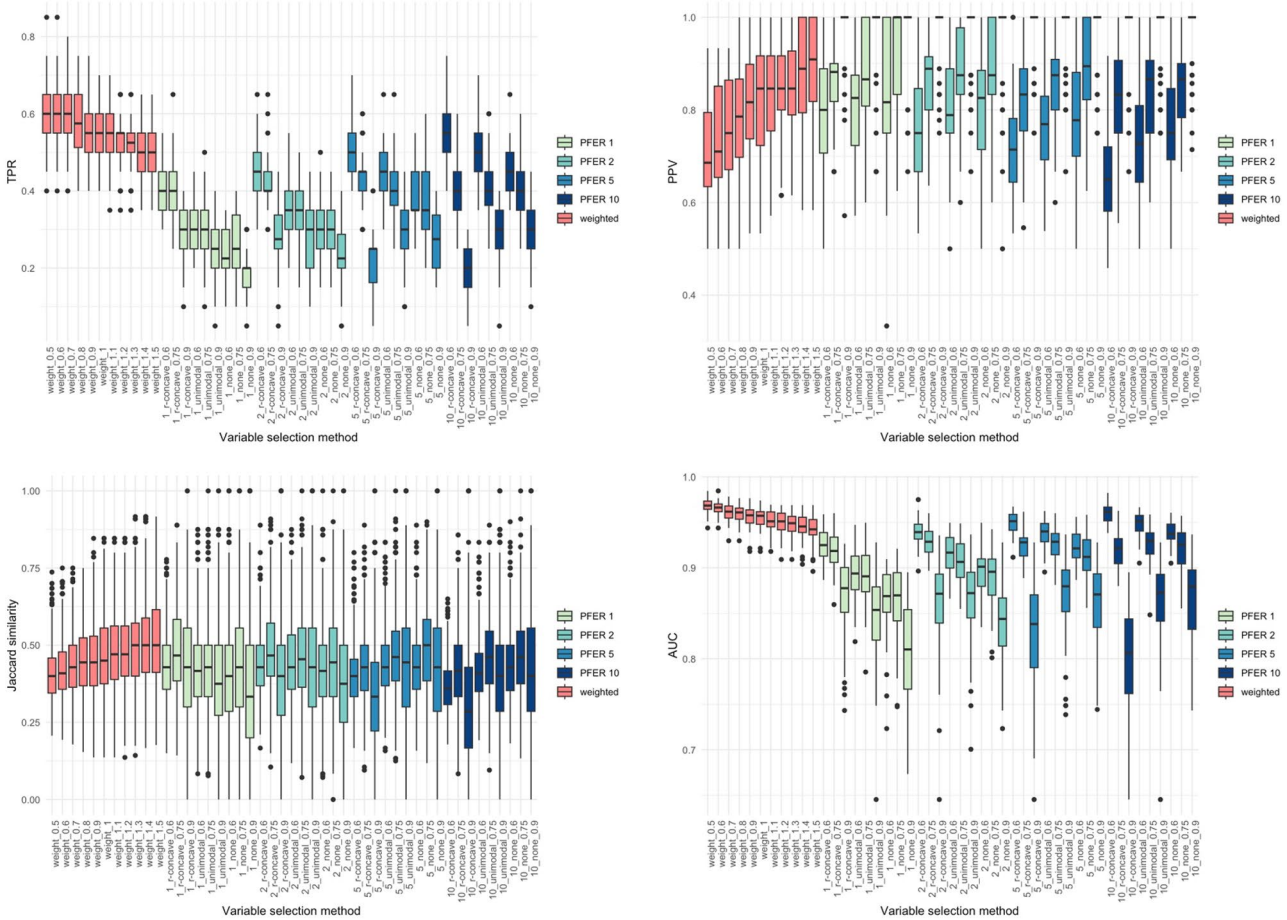
Box plots of true positive rate (TPR), positive predictive value (PPV), stability index (Jaccard similarity coefficient) and the area under the receiver operating characteristic curve (AUC) of the weighted stability selection and the stability selection in scenario 1 ($n=500$, ${\beta }_{j}$’s of the signal variables $\sim U(-\mathrm{3,3})$).

**Figure 3:**
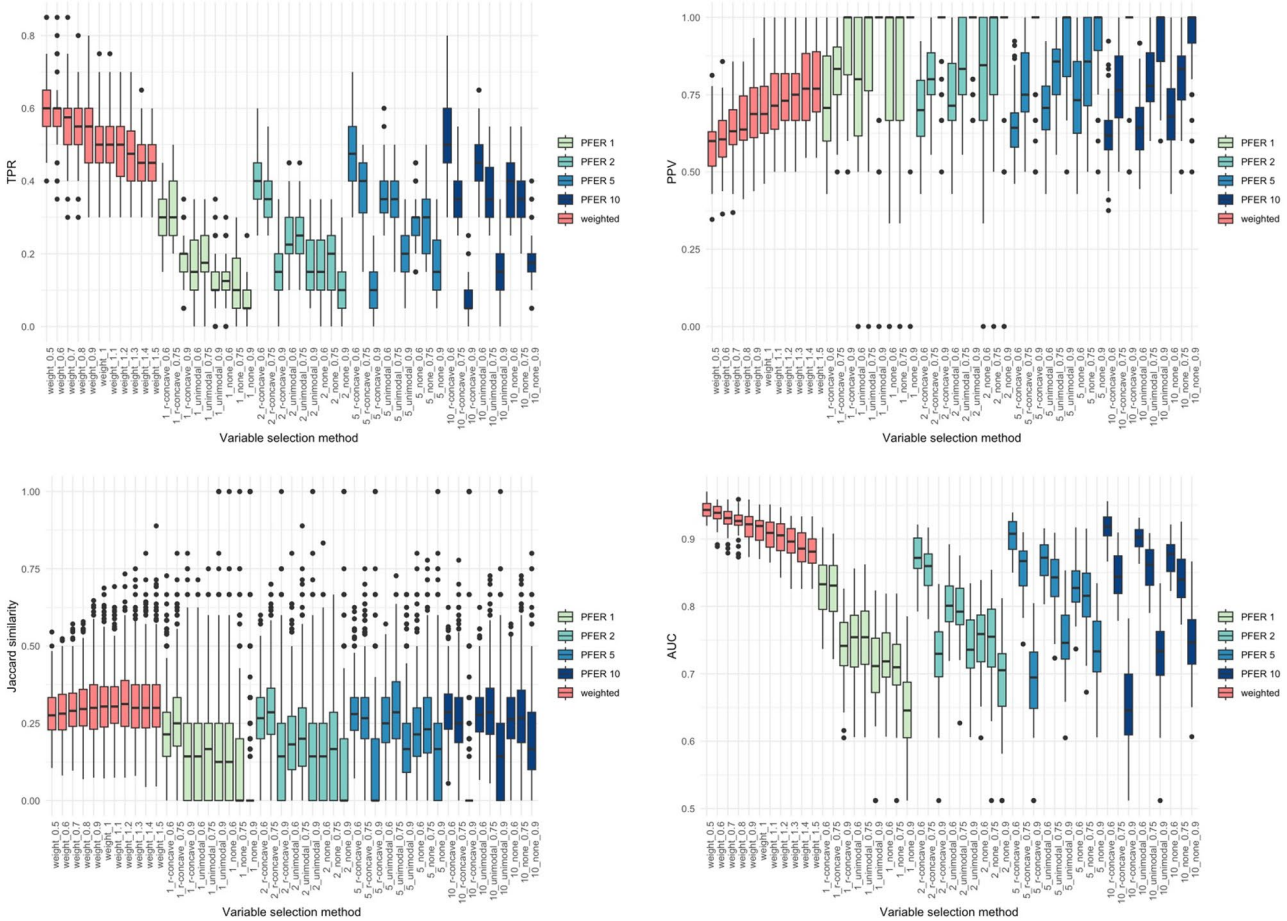
Box plots of true positive rate (TPR), positive predictive value (PPV), stability index (Jaccard similarity coefficient) and the area under the receiver operating characteristic curve (AUC) of the weighted stability selection and the stability selection in scenario 2 ($n=500$, ${\beta }_{j}$’s of the signal variables $\sim U(0.5,1.5)$).

**Figure 4:**
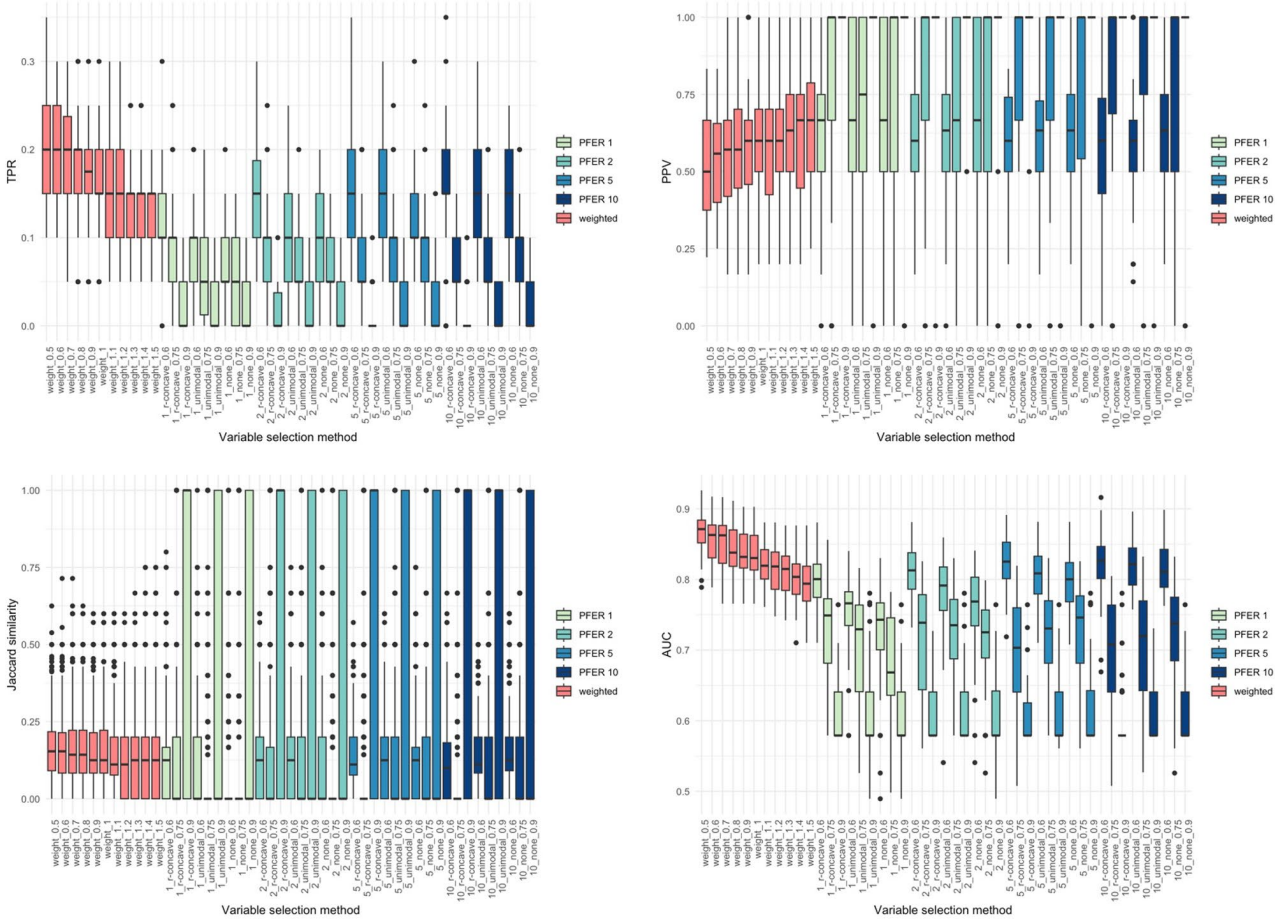
Box plots of true positive rate (TPR), positive predictive value (PPV), stability index (Jaccard similarity coefficient) and the area under the receiver operating characteristic curve (AUC) of the weighted stability selection and the stability selection in scenario 3 ($n=200$, ${\beta }_{j}$’s of the signal variables $\sim U(-\mathrm{3,3})$).

**Figure 5:**
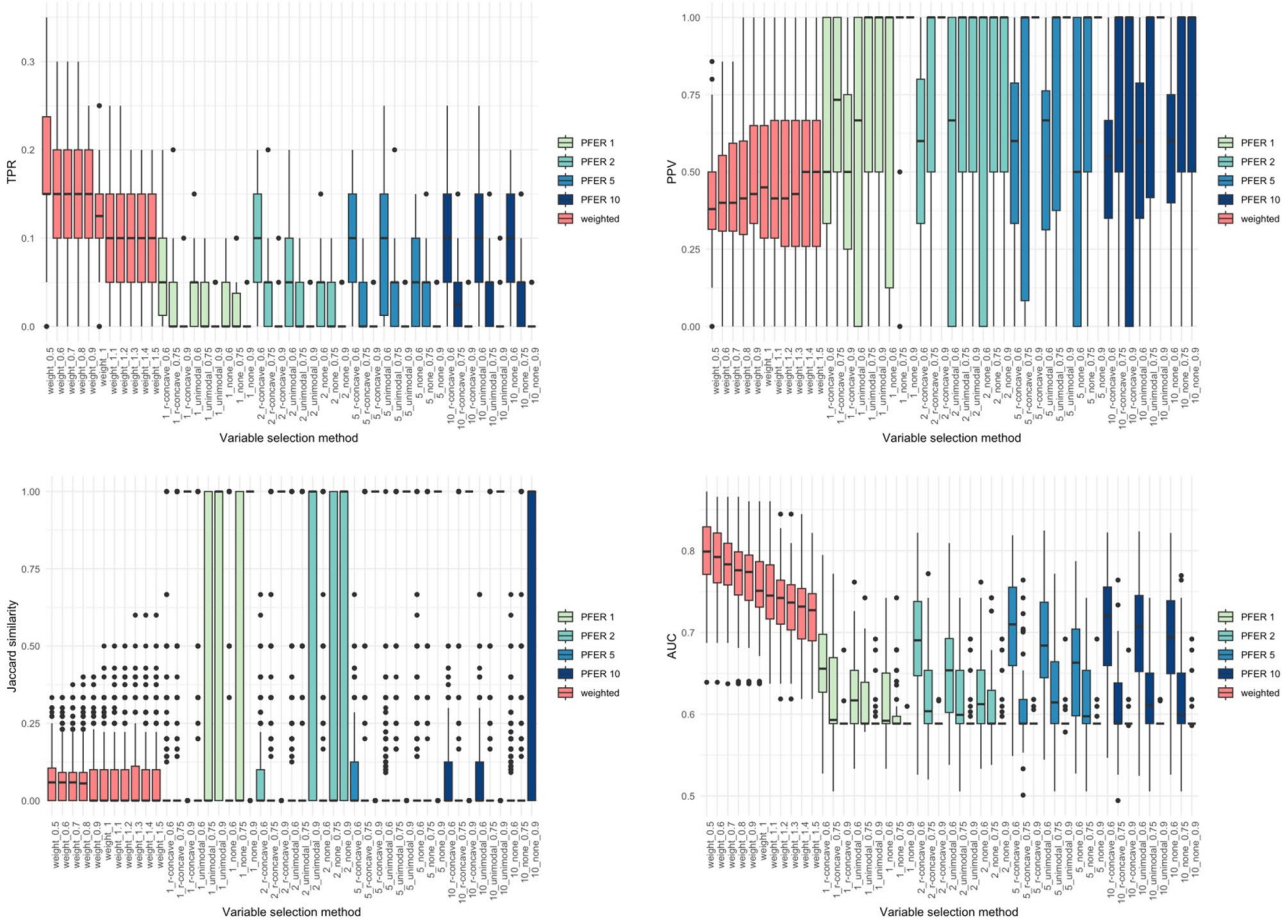
Box plots of true positive rate (TPR), positive predictive value (PPV), stability index (Jaccard similarity coefficient) and the area under the receiver operating characteristic curve (AUC) of the weighted stability selection and the stability selection in scenario 4 ($n=200$, ${\beta }_{j}$’s of the signal variables $\sim U(0.5,1.5)$).

Figures 2, 3, 4 and 5 present the TPR, PPV, Jaccard similarity coefficient, and AUC results for each scenario. Sørensen–Dice coefficient and Otsuka–Ochiai coefficient have the same tendency as Jaccard similarity coefficient; therefore, they are not shown. The simulation results of other scenarios are provided in Supplementary material. However, $n = 200$ and event prevalence $= 10\%$ scenarios are not available. All variable selection methods have poor results to the point that comparisons are meaningless.

The proposed weighted stability selection shows that the TPR decreases and the PPV increases as $\alpha$ increases. When considering stability selection, every parameter, $\mathrm{PFER upper bound}$, ${\pi }_{thr}$, and $\mathrm{error bound assumption}$ affected TPR and PPV. Similarly, the PPV was low if the parameter provided a high TPR. The AUC of both methods followed the trend of TPR. Therefore, when TPR was high, the AUC tended to be high. The proposed method's TPR, PPV, and stability were comparable to the usual stability selection. As expected, the AUCs of the proposed method was higher and had less variation, especially when the covariance structure was Toeplitz. This tendency gets stronger when the bigger ${\beta }_{j}$’s of the signal variables and the more ${p}_{signal}$.

## Application

We used radiomics data on aortic valve calcium^10^ and speech signal data on Parkinson's disease^11^ as examples to demonstrate the application of the proposed method in this study. Due to the large dimensionality of both data sets, appropriate methods for variable selection and model classifiers are necessary. We compared LASSO^2^ and stability selection^4^ with our proposed method, weighted stability selection. Each data set was divided into training, validation, and test sets. In the training set, variable selection was performed using stability selection and weighted stability selection for each parameter shown in simulation section. The validation set was used to determine the optimal parameters for the stability selection (PFER) and weighted stability selection ($\alpha$). The optimal parameters were defined as those that selected variables that fit the logistic regression with the highest AUC in the validation set. For LASSO^2^, we used tenfold cross-validation to select ${\lambda }_{min}$ and ${\lambda }_{1se}$. The final model of logistic regression using the variables selected by each method was fitted on the combined data of the training and validation sets, and the AUC of each method was calculated on the test set.

### Radiomics data on aortic valve calcium

Aortic stenosis (AS) is developed countries' most prevalent valvular heart disease. To prevent recurrent cardiovascular problems, the surgery timing in patients with severe AS is critical. Although echocardiography is a typical reference standard for determining AS severity, these tests are known to often be inconclusive. Low-gradient AS is the most common cause of inconclusive echocardiography. Evaluating the aortic valve calcium (AVC) score on cardiac computed tomography (CT) can help ameliorate this. Although AVC is important in AS, it is not the sole factor influencing the severity of the condition; some individuals with minimal AVC are diagnosed with hemodynamically severe AS. Other parameters that determine the severity of AS include AVC attenuation, shape, symmetry, or distribution. According to a previous study, the severity of AS is linked to the degree of AVC and the location of the valve^10^.

Radiomics refers to the extraction of high-dimensional, quantitative information from medical images in a high-throughput manner^17^. Since radiomics characteristics, such as volume, shape, texture, and high-order variables provide quantitative information about a region of interest (ROI) of AVC, a study of these aspects may produce predictive data concerning AS severity.

The entire dataset consisted of two clinical variables (age and sex) and 129 radiomic variables. Each radiomics variable was extracted using the AVIEW software (AVIEW Research, Coreline Soft Inc.). All radiomic variables are summarised in Table 1 in the Supplementary material. The total number of samples was 408 (240 with severe AS and 168 non-severe AS), with 201 randomly assigned to the training set, 85 to the validation set, and 122 to the test set.

**Table 1:** Results of variable selection for four different methods using radiomics data on aortic valve calcium.

Variable selection method	LASSO (min)	LASSO (1se)	Stability selection	Weighted stability selection
Selected parameter	$\uplambda =0.024$	$\uplambda =0.05$	$\mathrm{PFER upper bound}=10$ $\mathrm{error bound assumption}{=}^{\mathrm{^{\prime}}}\mathrm{r}-{\mathrm{concave}}^{\mathrm{^{\prime}}}$ ${\pi }_{thr}=0.6$	$\mathrm{\alpha }=0.2$
Number of variables selected	9	5	13	7
Selected variables	Sex 3D_Shape3D_SurfaceArea(mm2) 3D_Shape3D SphericalDisproportion 3D_Texture_Histo_Min 3D_Texture_Histo_Max 3D_Texture_Percentile_10 3D_Texture_GLRLM_SRE 3D_Texture_GLRLM_SRHGE 3D_Texture_GLDM_SDLGLE	3D_Shape3D_SurfaceArea(mm2) 3D_Shape3D_SphericalDisproportion 3D_Texture_Percentile_25 3D_Texture_GLDM_DN 3D_Texture_GLDM_SDLGLE	Sex 3D_Shape3D_SurfaceAreaToVolumeRatio 3D_Shape3D_Roundness 3D_Shape3D_SphericalDisproportion 3D_Shape3D_Longest1stAxisOnSagittal(mm) 3D_Shape3D_Longest2ndAxisOnCoronal(mm) 3D_Shape3D_Flatness 3D_Texture_FirstOrder_Min 3D_Texture_Histo_Min 3D_Texture_Percentile_10 3D_Texture_GLRLM_SRE 3D_Texture_GLDM_SDLGLE 3D_Texture_GLDM_LDHGLE	Sex 3D_Shape3D_SurfaceArea (mm2) 3D_Shape3D_SphericalDisproportion 3D_Texture_FirstOrder_Min 3D_Texture_Percentile_10 3D_Texture_GLRLM_SRE 3D_Texture_GLDM_SDLGLE
AUC	0.906	0.902	0.895	0.914

Table 1 presents the results of each variable selection method’s selected parameter settings, selected variables, and the fitted models AUC with the selected variables. Weighted stability selection presented a higher AUC with fewer parameters than stability selection and LASSO. Weighted stability selection selected 7 variables: “Sex”, “3D_Shape3D_SphericalDisproportion (a ratio that compares the surface area of a tumor region to the surface area of a sphere having the same volume as the tumor region)”, “3D_Shape3D_SurfaceArea (mm2) (the sum of all calculated sub areas)”, “3D_Texture_FirstOrder_Min (the minimum of a set of voxels included in the ROI)”, “3D_Texture_Percentile_10 (the 10th percentile of a set of voxels included in the ROI)”, “3D_Texture_GLRLM_SRE (the texture measure of an image based on the frequency of short, uninterrupted segments of similar pixel values)” and “3D_Texture_GLDM_SDLGLE (the measure of how smaller gray-level values are dependent on each other in a joint distribution)”.

### Speech signal data on Parkinson's disease

Parkinson's disease (PD) is a degenerative disease that progresses over time and is marked by numerous motor and non-motor symptoms. It is the second most frequently occurring neurodegenerative disease, after Alzheimer's, in individuals over the age of 60. There has been a growing interest in developing telediagnosis and telemonitoring systems for PD that measure the motor system impairments caused by the disease. As vocal impairments are prevalent in approximately 90% of PD patients in the early stages of the disease, speech signal processing algorithms have been employed to extract useful clinical information for assessing PD^11^.

Sakar et al.^11^ used a tunable Q-factor wavelet transform (TQWT)^18,19^ to extract variables from voice signals of PD patients, which offers a higher frequency resolution than the classical discrete wavelet transforms. They recorded the voices of 252 patients (188 with PD and 64 healthy) three times and extracted 752 variables from the recordings using other state-of-the-art variable extraction methods and TQWT speech signal processing algorithm. Additional details about these variables are available in Sakar et al.^11^. The data has 753 variables, including the gender variable. 145 samples were randomly assigned to the training set, 42 to the validation set, and 65 to the test set.

Table 2 presents the results of each variable selection method’s selected parameter settings, selected variables, and the AUC of the fitted model with the selected variables. Consistent with the results in section "Radiomics data on aortic valve calcium", the weighted stability selection achieved a higher AUC with fewer parameters than the stability selection and LASSO. Weighted stability selection selected 4 variables: “mean_MFCC_2nd_coef (the average value of the second Mel Frequency Cepstral Coefficients (MFCC) coefficient across multiple frames of a speech signal)”, “std_delta_delta_log_energy (the standard deviation of the change in the delta delta log energy across multiple frames of a speech signal)”, “std_6th_delta_delta (the standard deviation of the sixth order delta-delta coefficients across multiple frames of a speech signal)”, and “tqwt_meanValue_dec_5 (the mean value of the TQWT coefficients at the 5th level of decomposition)”.

**Table 2:** Results of variable selection for four different methods using a speech signal data on Parkinson's disease.

Variable selection method	LASSO (min)		LASSO (1se)	Stability selection	Weighted stability selection
Selected parameter	$\uplambda =0.014$		$\uplambda =0.025$	$\mathrm{PFER upper bound}=2$ $\mathrm{error bound assumption}{=}^{\mathrm{^{\prime}}}\mathrm{r}-{\mathrm{concave}}^{\mathrm{^{\prime}}}$ ${\pi }_{thr}=0.6$	$\mathrm{\alpha }=2.0$
Number of variables selected	66		40	11	4
Selected variables	gender DFA locDbShimmer apq11Shimmer meanHarmToNoiseHarmonicity f2 GQ_prc5_95 GNE_NSR_TKEO IMF_SNR_SEO IMF_NSR_TKEO mean_MFCC_1st_coef mean_MFCC_3rd_coef mean_MFCC_6th_coef mean_MFCC_7th_coef mean_MFCC_8th_coef mean_MFCC_10th_coef mean_MFCC_11th_coef mean_delta_log_energy mean_2nd_delta mean_11th_delta_delta std_8th_delta std_delta_delta_log_energy std_6th_delta_delta std_7th_delta_delta std_8th_delta_delta det_entropy_log_4_coef det_LT_entropy_shannon_4_coef tqwt_energy_dec_11 tqwt_energy_dec_15 tqwt_entropy_shannon_dec_25 tqwt_entropy_shannon_dec_26 tqwt_entropy_shannon_dec_27 tqwt_entropy_shannon_dec_34	tqwt_entropy_log_dec_26 tqwt_entropy_log_dec_33 tqwt_TKEO_mean_dec_17 tqwt_TKEO_mean_dec_20 tqwt_TKEO_mean_dec_21 tqwt_TKEO_mean_dec_30 tqwt_TKEO_std_dec_11 tqwt_TKEO_std_dec_12 tqwt_TKEO_std_dec_20 tqwt_medianValue_dec_5 tqwt_medianValue_dec_10 tqwt_medianValue_dec_12 tqwt_medianValue_dec_25 tqwt_medianValue_dec_29 tqwt_medianValue_dec_33 tqwt_medianValue_dec_36 tqwt_meanValue_dec_5 tqwt_meanValue_dec_7 tqwt_meanValue_dec_12 tqwt_meanValue_dec_22 tqwt_meanValue_dec_25 tqwt_stdValue_dec_6 tqwt_stdValue_dec_21 tqwt_minValue_dec_12 tqwt_maxValue_dec_6 tqwt_skewnessValue_dec_6 tqwt_skewnessValue_dec_17 tqwt_skewnessValue_dec_27 tqwt_skewnessValue_dec_35 tqwt_kurtosisValue_dec_22 tqwt_kurtosisValue_dec_27 tqwt_kurtosisValue_dec_30 tqwt_kurtosisValue_dec_33	gender DFA apq11Shimmer meanHarmToNoiseHarmonicity GNE_NSR_TKEO IMF_SNR_SEO mean_MFCC_1st_coef mean_MFCC_2nd_coef mean_MFCC_3rd_coef mean_MFCC_6th_coef mean_MFCC_7th_coef mean_MFCC_8th_coef mean_2nd_delta std_8th_delta std_delta_delta_log_energy std_6th_delta_delta std_7th_delta_delta std_8th_delta_delta det_LT_entropy_shannon_4_coef tqwt_energy_dec_11 tqwt_energy_dec_12 tqwt_energy_dec_15 tqwt_entropy_shannon_dec_25 tqwt_entropy_shannon_dec_34 tqwt_entropy_log_dec_26 tqwt_entropy_log_dec_33 tqwt_TKEO_mean_dec_17 tqwt_TKEO_mean_dec_21 tqwt_TKEO_mean_dec_30 tqwt_TKEO_std_dec_11 tqwt_medianValue_dec_5 tqwt_medianValue_dec_10 tqwt_medianValue_dec_33 tqwt_medianValue_dec_36 tqwt_meanValue_dec_5 tqwt_stdValue_dec_6 tqwt_maxValue_dec_6 tqwt_skewnessValue_dec_27 tqwt_kurtosisValue_dec_27 tqwt_kurtosisValue_dec_33	DFA mean_MFCC_2nd_coef std_delta_delta_log_energy std_6th_delta_delta tqwt_energy_dec_11 tqwt_entropy_log_dec_26 tqwt_meanValue_dec_5 tqwt_stdValue_dec_6 tqwt_kurtosisValue_dec_26 tqwt_kurtosisValue_dec_27 tqwt_kurtosisValue_dec_33	mean_MFCC_2nd_coef std_delta_delta_log_energy std_6th_delta_delta tqwt_meanValue_dec_5
AUC	0.669		0.756	0.776	0.826

## Conclusion

This paper proposes a weighted stability selection to improve stability selection. Weighted stability selection improves AUC prediction and enables more straightforward parameter settings.

Similar to the existing methods, the proposed weighted stability selection concentrates on frequently selected variables through repeated variable selection, which is accomplished by subsampling data many times. However, we go one step further. When calculating the selected frequency of each variable, we assign different weights to each variable using the AUC of the model fitted on the selected variables. Using this process, the weighted stability selection makes it possible that the AUC of the selected model is higher than that of the selected model based on the usual stability selection.

We conducted extensive simulations under various scenarios by considering the degree of $n$, $p$, ${p}_{signal}$, ${\beta }_{j}$’s of the signal variables, event prevalence, and the covariance structures. The performance of the proposed method is comparable to that of stability selection in terms of TPR, PPV, and stability. The AUCs of the validation set for the proposed method was always higher and had a smaller variance when the covariance structure was Toeplitz. The excellent performance in the case of Toeplitz, a covariance structure similar to the real data, shows the value of the proposed method.

Using the radiomics data on aortic valve calcium^10^ and speech signal data on Parkinson's disease^11^, variable selection was performed. We found that weighted stability selection produced higher AUCs with fewer variables than LASSO and stability selection. These are meaningful results because the weighted stability selection demonstrates that researchers who want a model with a higher AUC can use it intuitively in real situations. On stability selection, it is necessary to select a combination based on the understanding of three parameters: $\mathrm{PFER upper bound}$, $\mathrm{error bound assumption}$, and ${\pi }_{thr}$ whereas in the case of the proposed weighted stability selection, only $\alpha$ is used. Researchers can easily change the number of variables selected by changing $\alpha$ as the purpose of variable selection.

While the stability selection allows the application of various variable selection methods as deductive methods, this study used an inductive approach with binary data and logistic regression. This indicates that this study has potential for further development. Application to other variable selection methods and other data types, such as survival data, are also promising areas for future research.

## Software

Sample R code, together with a simulated data example, is available on the author’s GitHub page at https://github.com/yonghankwon0/weighted_stability_selection.

## Supplementary Information

Supplementary Information.

## Data Availability

The datasets used and/or analysed during the current study available from the corresponding author on reasonable request.

## References

[1] Hastie T, Tibshirani R, Friedman JH (2009). The Elements of Statistical Learning: Data Mining, Inference, and Prediction.

[2] Tibshirani R (1996). Regression shrinkage and selection via the lasso. J. R. Stat. Soc. Ser. B Methodol..

[3] Meinshausen N, Bühlmann P (2010). Stability selection. J. R. Stat. Soc. Ser. B Methodol. B.

[4] Shah RD, Samworth RJ (2013). Variable selection with error control: Another look at stability selection. J. R. Stat. Soc. Ser. B Methodol..

[5] Marbach D (2012). Wisdom of crowds for robust gene network inference. Nat. Methods.

[6] Haury AC, Mordelet F, Vera-Licona P, Vert JP (2012). TIGRESS: Trustful inference of gene regulation using stability selection. BMC Syst. Biol..

[7] Hu X, Hu Y, Wu F, Leung RWT, Qin J (2020). Integration of single-cell multi-omics for gene regulatory network inference. Comput. Struct. Biotechnol. J..

[8] De-Groot P (2021). A Faecal microbiota transplantation halts progression of human new-onset type 1 diabetes in a randomised controlled trial. Gut.

[9] Hanley JA, McNeil BJ (1982). The meaning and use of the area under a receiver operating characteristic (ROC) curve. Radiology.

[10] Kang NG, Suh YJ, Han K, Kim YJ, Choi BW (2021). Performance of prediction models for diagnosing severe aortic stenosis based on aortic valve calcium on cardiac computed tomography: Incorporation of Radiomics and Machine Learning. Korean J. Radiol..

[11] Sakar CO (2019). A comparative analysis of speech signal processing algorithms for Parkinson’s disease classification and the use of the tunable Q-factor wavelet transform. Appl. Soft Comput..

[12] Hastie T, Tibshirani R, Wainwright M (2015). Statistical Learning with Sparsity: The Lasso and Generalizations.

[13] Nogueira S, Sechidis K, Brown G (2017). On the stability of feature selection algorithms. J. Mach. Learn. Res..

[14] Jaccard P (1908). Nouvelles recherches sur la distribution florale. Bull. Soc. Vaud. Sci. Nat..

[15] Akira O (1957). Zoogeographical studies on the soleoid fishes found in Japan and its neighbouring regions–II. Bull. Jpn. Soc. Sci. Fish..

[16] Dice LR (1945). Measures of the amount of ecologic association between species. Ecology.

[17] Van Timmeren JE (2020). Radiomics in medical imaging—“how-to” guide and critical reflection. Insights Imaging.

[18] Selesnick IW (2011). Wavelet transform with tunable Q-factor. IEEE Trans. Signal Process..

[19] Selesnick IW (2011). Resonance-based signal decomposition: A new sparsity-enabled signal analysis method. Signal Process..

