# Supplementary material for: Stability selection for LASSO with weights based on AUC
Source: Sci Rep. 2023 Mar 30;13:5207. doi: 10.1038/s41598-023-32517-4 (PMC10063650; doi:10.1038/s41598-023-32517-4)
Supplement: Supplementary file 1 — Supplementary Information. [file 41598_2023_32517_MOESM1_ESM.docx]

**Supplementary material to ‘Stability selection for LASSO with weights based on AUC’**

Yonghan Kwon

Departments of Biostatistics and Computing, Yonsei University Graduate School, 50-1, Yonsei-ro, Seodaemun-gu, Seoul, Republic of Korea

YONGHANKWON0@yuhs.ac

Kyunghwa Han

Department of Radiology, Research Institute of Radiological Science, and Center for Clinical Imaging Data Science, Yonsei University College of Medicine, 50-1, Yonsei-ro, Seodaemun-gu, Seoul, Republic of Korea

KHHAN@yuhs.ac

Young Joo Suh

Department of Radiology, Research Institute of Radiological Science, and Center for Clinical Imaging Data Science, Yonsei University College of Medicine, 50-1, Yonsei-ro, Seodaemun-gu, Seoul, Republic of Korea

RONGZU@yuhs.ac

Inkyung Jung*

Division of Biostatistics, Department of Biomedical Systems Informatics, Yonsei University College of Medicine 50-1, Yonsei-ro, Seodaemun-gu, Seoul, Republic of Korea

IJUNG@yuhs.ac

| Variable category | Extracted CT radiomic variables |
| --- | --- |
| 3D size and shape variables  (23 variables) | Shape3D_Volume(mm3) |
|  | Shape3D_SurfaceArea (mm2) |
|  | Shape3D_SurfaceAreaToVolumeRatio |
|  | Shape3D_Sphericity |
|  | Shape3D_Compactness |
|  | Shape3D_Compactness2 |
|  | Shape3D_Compactness3 |
|  | Shape3D_Roundness |
|  | Shape3D_Circularity |
|  | Shape3D_SphericalDisproportion |
|  | Shape3D_Longest1stAxis (mm) |
|  | Shape3D_Longest2ndAxis (mm) |
|  | Shape3D_Longest1stAxisOnAxial (mm) |
|  | Shape3D_Longest2ndAxisOnAxial (mm) |
|  | Shape3D_Longest1stAxisOnSagittal (mm) |
|  | Shape3D_Longest2ndAxisOnSagittal (mm) |
|  | Shape3D_Longest1stAxisOnCoronal (mm) |
|  | Shape3D_Longest2ndAxisOnCoronal (mm) |
|  | Shape3D_PCA1stMajorStd (mm) |
|  | Shape3D_PCA2ndMajorStd (mm) |
|  | Shape3D_PCA3rdMajorStd (mm) |
|  | Shape3D_Elongation |
|  | Shape3D_Flatness |
| First order and histogram-based  variables (29 variables) | Texture_FirstOrder_Energy |
|  | Texture_FirstOrder_TotalEnergy |
|  | Texture_FirstOrder_Min |
|  | Texture_FirstOrder_Max |
|  | Texture_FirstOrder_Range |
|  | Texture_FirstOrder_Mean |
|  | Texture_FirstOrder_MAD |
|  | Texture_FirstOrder_rMAD |
|  | Texture_FirstOrder_RMS |
|  | Texture_FirstOrder_Std |
|  | Texture_FirstOrder_Skewness |
|  | Texture_FirstOrder_ExcessKurtosis |
|  | Texture_FirstOrder_Variance |
|  | Texture_FirstOrder_InterquartileRange |
|  | Texture_Histo_Mean |
|  | Texture_Histo_Std |
|  | Texture_Histo_Skewness |
|  | Texture_Histo_ExcessKurtosis |
|  | Texture_Histo_Energy |
|  | Texture_Histo_Entropy |
|  | Texture_Histo_Min |
|  | Texture_Histo_Max |
|  | Texture_Histo_VoxelCount |
|  | Texture_Percentile_10 |
|  | Texture_Percentile_25 |
|  | Texture_Percentile_50 |
|  | Texture_Percentile_75 |
|  | Texture_Percentile_90 |
|  | Texture_Percentile_95 |
| Gray Level Co-occurrence Matrix (GLCM) variables (22 variables) | Texture_GLCM_ASM |
|  | Texture_GLCM_IDM |
|  | Texture_GLCM_IDMN |
|  | Texture_GLCM_Homogeneity |
|  | Texture_GLCM_HomogeneityNormalized |
|  | Texture_GLCM_InverseVariance |
|  | Texture_GLCM_Contrast |
|  | Texture_GLCM_Correlation |
|  | Texture_GLCM_Autocor |
|  | Texture_GLCM_Entropy |
|  | Texture_GLCM_CP |
|  | Texture_GLCM_CS |
|  | Texture_GLCM_CT |
|  | Texture_GLCM_SumEntropy |
|  | Texture_GLCM_DiffAverage, |
|  | Texture_GLCM_DiffEntropy |
|  | Texture_GLCM_DiffVariance |
|  | Texture_GLCM_IMC1 |
|  | Texture_GLCM_IMC2 |
|  | Texture_GLCM_MCC |
|  | Texture_GLCM_MaxProb |
|  | Texture_GLCM_SumAverage |
| Gray Level Run Length Matrix (GLRLM) variables (14 variables) | Texture_GLRLM_SRE |
|  | Texture_GLRLM_LRE |
|  | Texture_GLRLM_LGRE |
|  | Texture_GLRLM_HGRE |
|  | Texture_GLRLM_SRLGE |
|  | Texture_GLRLM_SRHGE |
|  | Texture_GLRLM_LRLGE |
|  | Texture_GLRLM_LRHGE |
|  | Texture_GLRLM_GNUN |
|  | Texture_GLRLM_RLNUN |
|  | Texture_GLRLM_RP |
|  | Texture_GLRLM_RV |
|  | Texture_GLRLM_RE |
|  | Texture_GLRLM_GLV |
| Gray Level Size Zone (GLSZM)  variables (16 variables) | Texture_GLSZM_SAE |
|  | Texture_GLSZM_LAE |
|  | Texture_GLSZM_GLN |
|  | Texture_GLSZM_GLNN |
|  | Texture_GLSZM_SZN |
|  | Texture_GLSZM_SZNN |
|  | Texture_GLSZM_ZP |
|  | Texture_GLSZM_GLV |
|  | Texture_GLSZM_ZV |
|  | Texture_GLSZM_ZE |
|  | Texture_GLSZM_LGLZE |
|  | Texture_GLSZM_HGLZE |
|  | Texture_GLSZM_SALGLE |
|  | Texture_GLSZM_SAHGLE |
|  | Texture_GLSZM_LALGLE |
|  | Texture_GLSZM_LAHGLE |
| Gray Level Dependence Matrix (GLDM) variables (14 variables) | Texture_GLDM_SDE |
|  | Texture_GLDM_LDE |
|  | Texture_GLDM_GLN |
|  | Texture_GLDM_DN |
|  | Texture_GLDM_DNN |
|  | Texture_GLDM_GLV |
|  | Texture_GLDM_DV |
|  | Texture_GLDM_DE |
|  | Texture_GLDM_LGLE |
|  | Texture_GLDM_HGLE |
|  | Texture_GLDM_SDLGLE |
|  | Texture_GLDM_SDHGLE |
|  | Texture_GLDM_LDLGLE |
|  | Texture_GLDM_LDHGLE |
| Neighbouring Gray Tone Difference Matrix (NGTDM) variables  (5 variables) | Texture_NGTDM_Coarseness |
|  | Texture_NGTDM_Contrast |
|  | Texture_NGTDM_Busyness |
|  | Texture_NGTDM_Complexity |
|  | Texture_NGTDM_Strength |
| Moment variables (3 variables) | Texture_Moment_J1 |
|  | Texture_Moment_J2 |
|  | Texture_Moment_J3 |
| Gradient variables (2 variables) | Texture_Grad_Mean |
|  | Texture_Grad_Std |
| Fractal variables (1 variables) | FractalDimension |

Table 1. Extracted radiomics variables by AVIEW software (AVIEW Research, Coreline Soft Inc.).


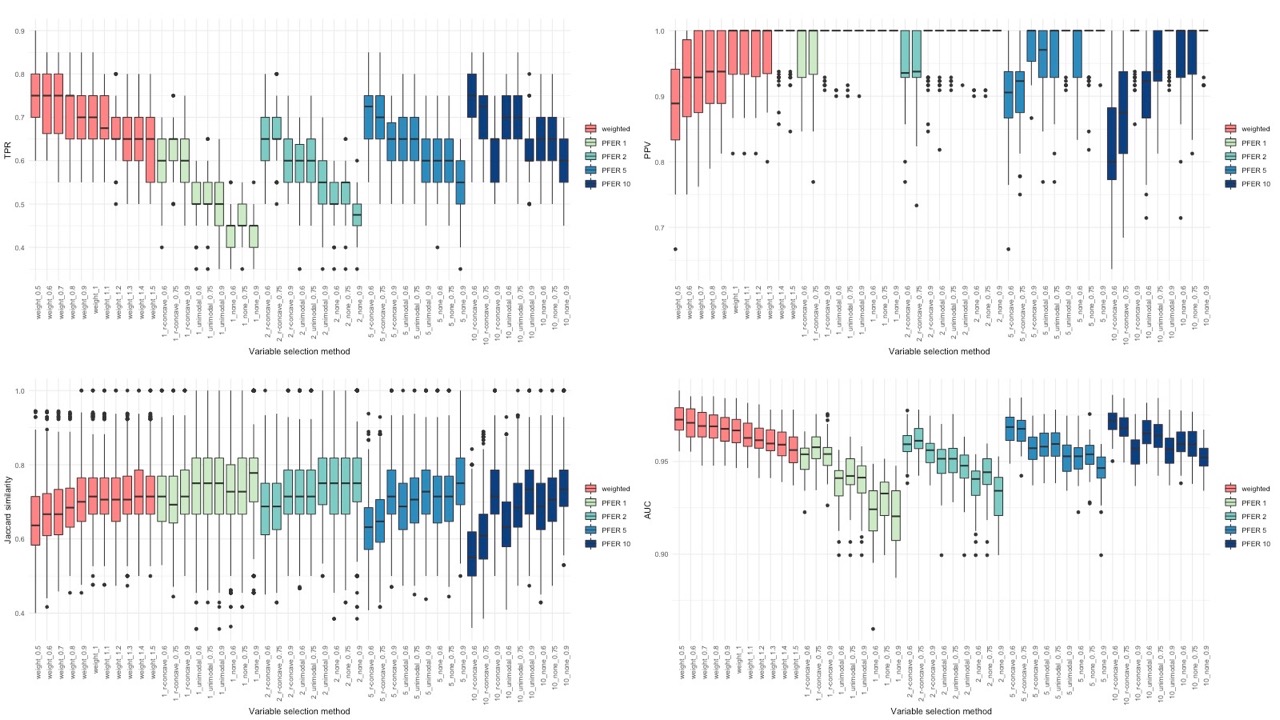


Box plots of scenario 1. $n=500, p=1000, p_{signal}=20, \beta_{j}$’s of the signal variables $\sim U\left( -3,3 \right)$, event prevalence$=0.5$ and the covariance structure of $X=$independent.


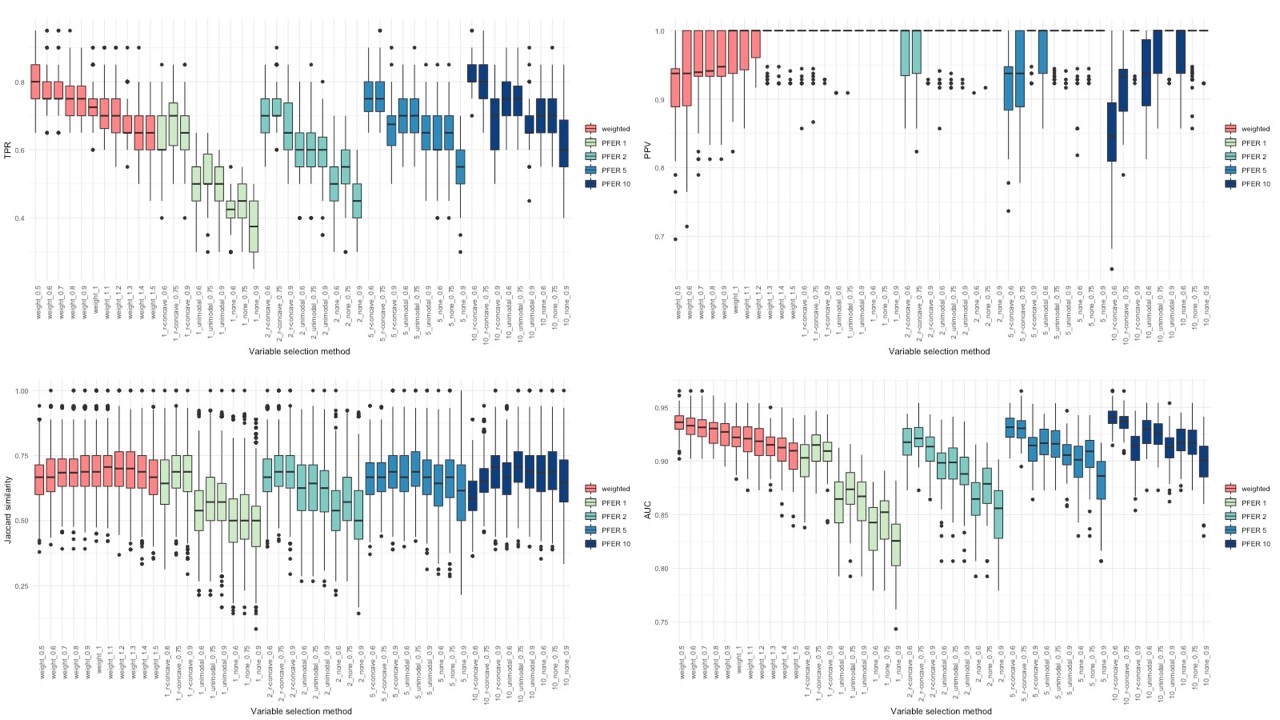


Box plots of scenario 2. $n=500, p=1000, p_{signal}=20, \beta_{j}$’s of the signal variables $\sim U\left( 0.5,1.5 \right)$, event prevalence$=0.5$ and the covariance structure of $X=$independent.


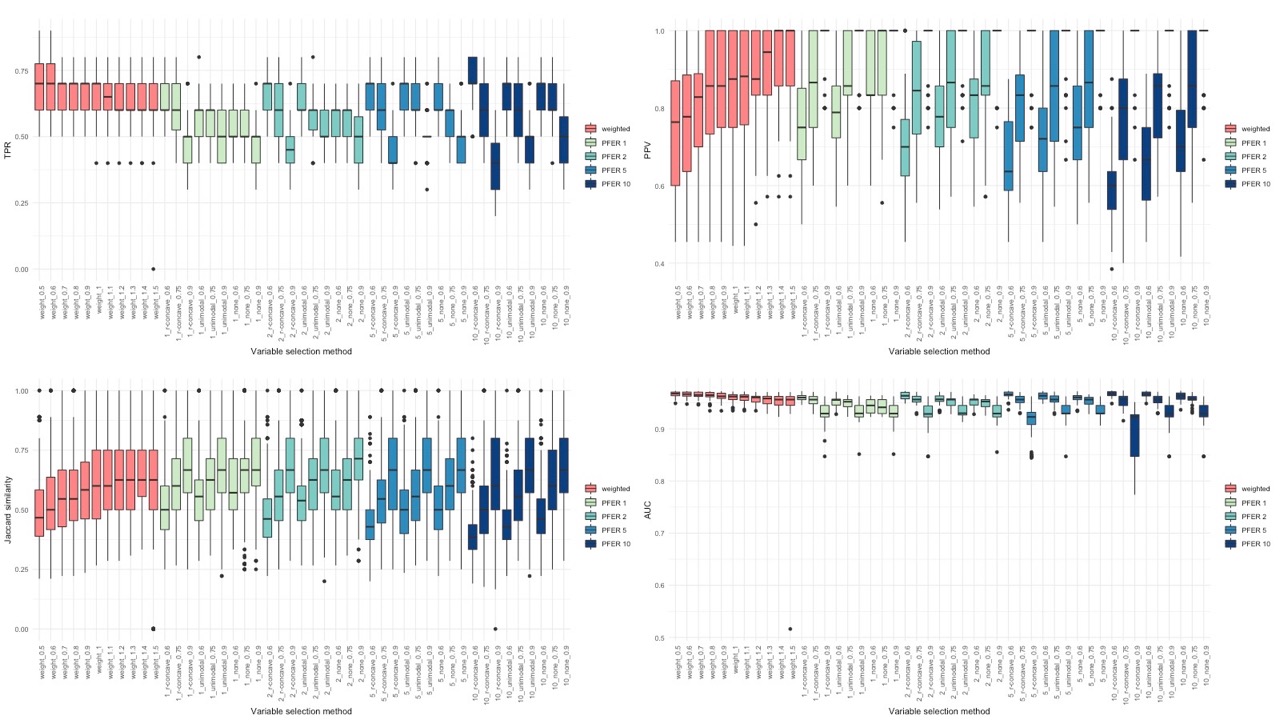


Box plots of scenario 3. $n=500, p=1000, p_{signal}=10, \beta_{j}$’s of the signal variables $\sim U\left( -3,3 \right)$, event prevalence$=0.5$ and the covariance structure of $X=$Toeplitz.


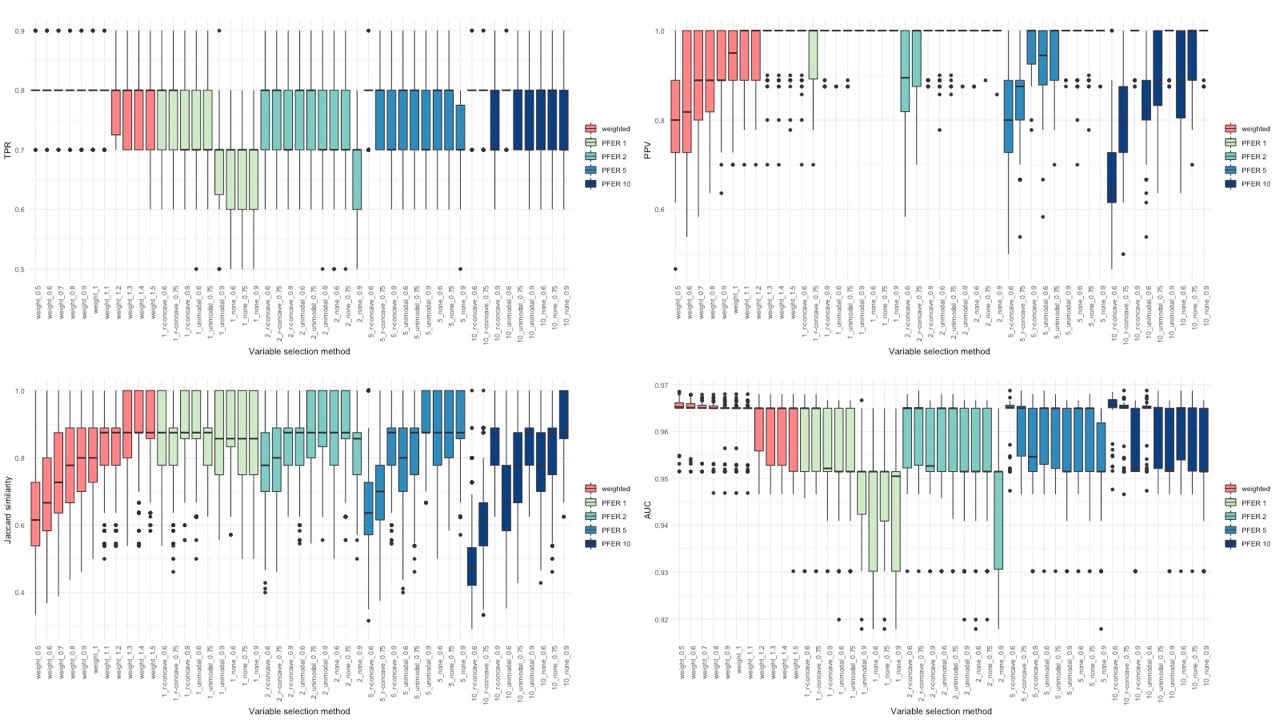


Box plots of scenario 4. $n=500, p=1000, p_{signal}=10, \beta_{j}$’s of the signal variables $\sim U\left( -3,3 \right)$, event prevalence$=0.5$ and the covariance structure of $X=$independent.


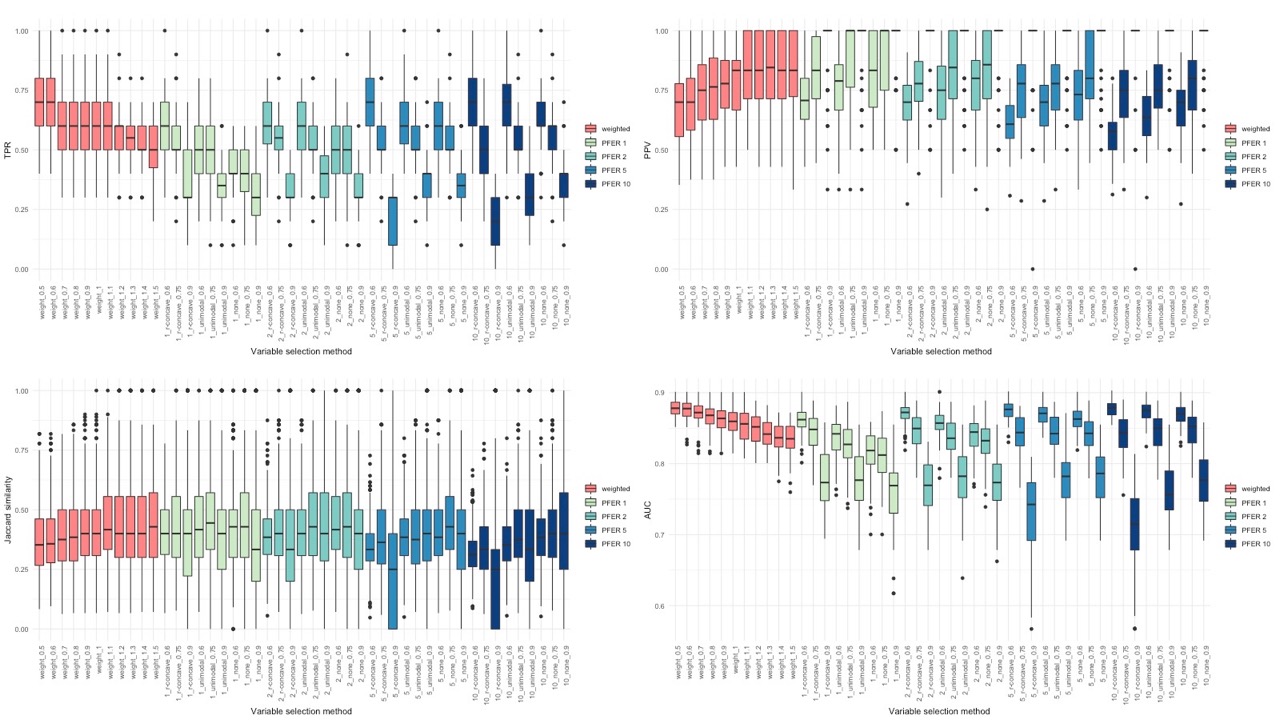


Box plots of scenario 5. $n=500, p=1000, p_{signal}=10, \beta_{j}$’s of the signal variables $\sim U\left( 0.5,1.5 \right)$, event prevalence$=0.5$ and the covariance structure of $X=$Toeplitz.


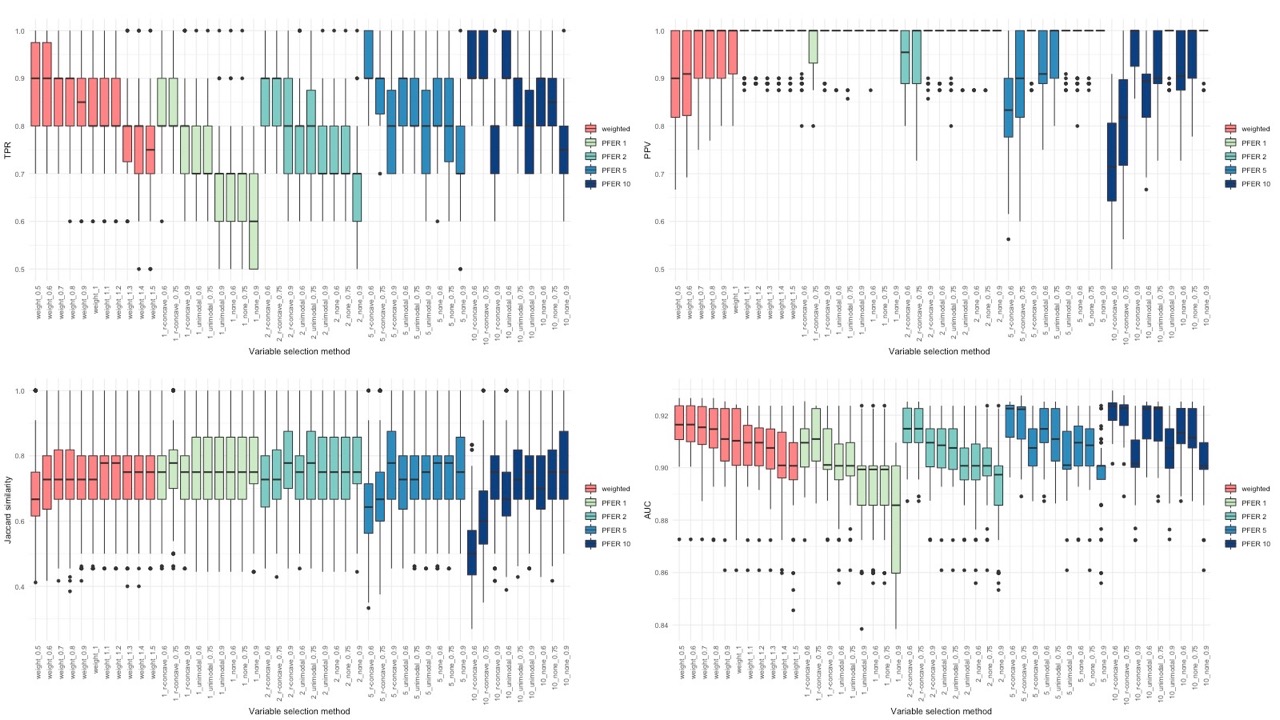


Box plots of scenario 6. $n=500, p=1000, p_{signal}=10, \beta_{j}$’s of the signal variables $\sim U\left( 0.5,1.5 \right)$, event prevalence$=0.5$ and the covariance structure of $X=$independent.


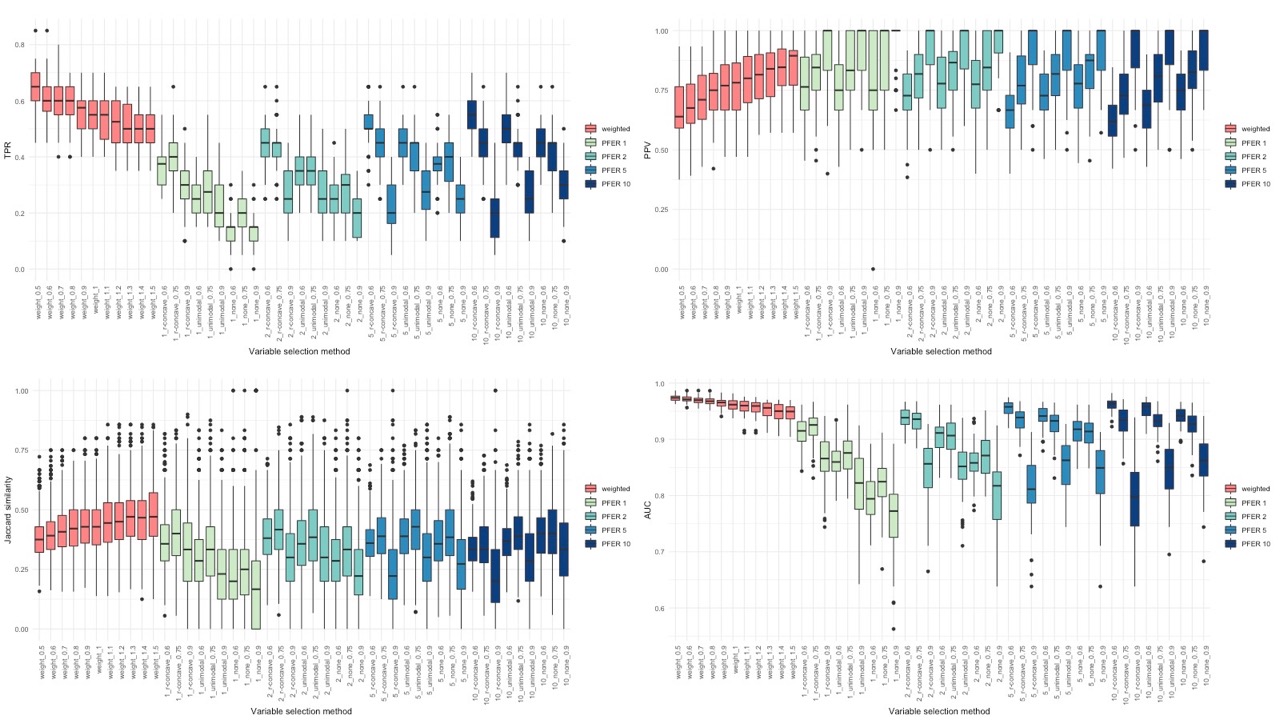


Box plots of scenario 7. $n=500, p=700, p_{signal}=20, \beta_{j}$’s of the signal variables $\sim U\left( -3,3 \right)$, event prevalence$=0.5$ and the covariance structure of $X=$Toeplitz.


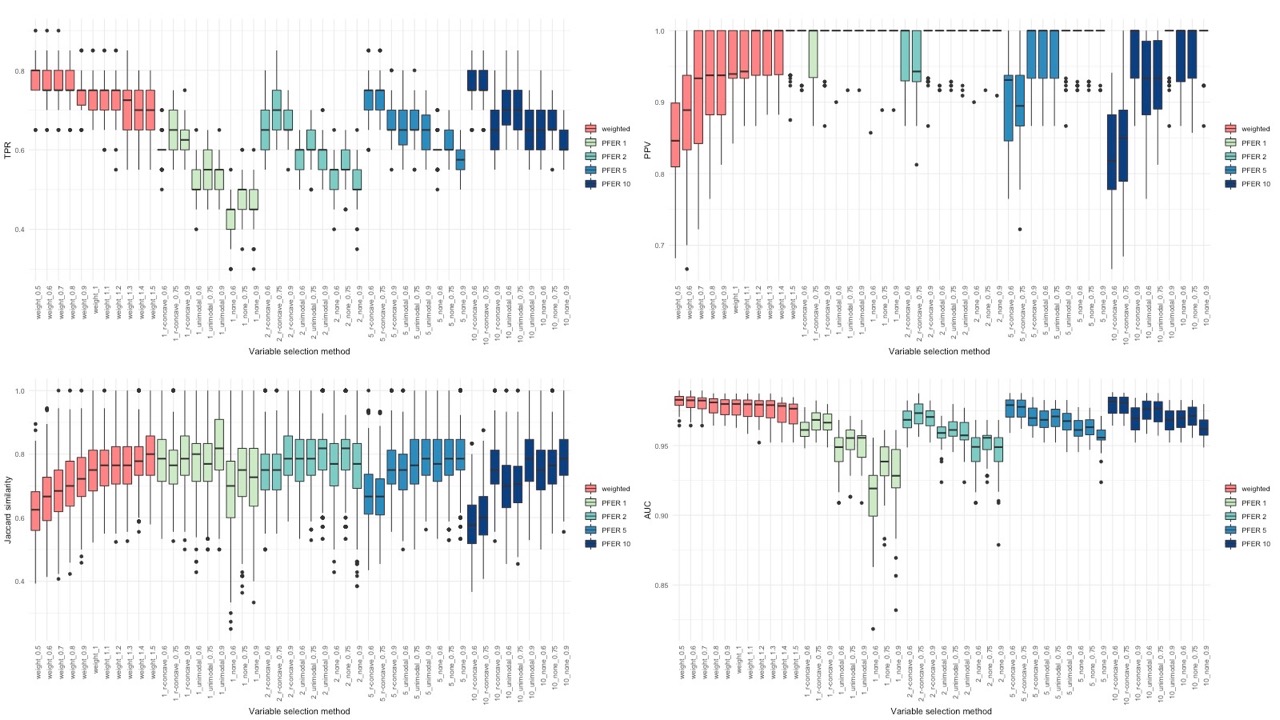


Box plots of scenario 8. $n=500, p=700, p_{signal}=20, \beta_{j}$’s of the signal variables $\sim U\left( -3,3 \right)$, event prevalence$=0.5$ and the covariance structure of $X=$independent.


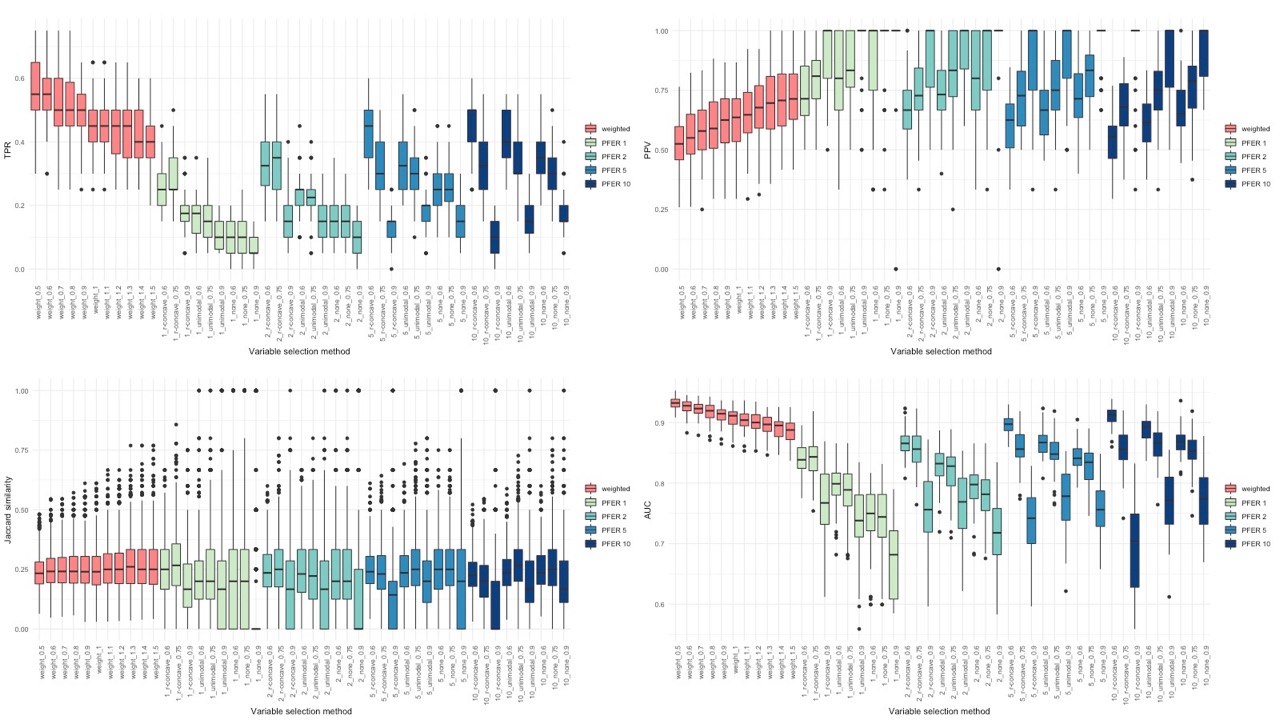


Box plots of scenario 9. $n=500, p=700, p_{signal}=20, \beta_{j}$’s of the signal variables $\sim U\left( 0.5,1.5 \right)$, event prevalence$=0.5$ and the covariance structure of $X=$Toeplitz.


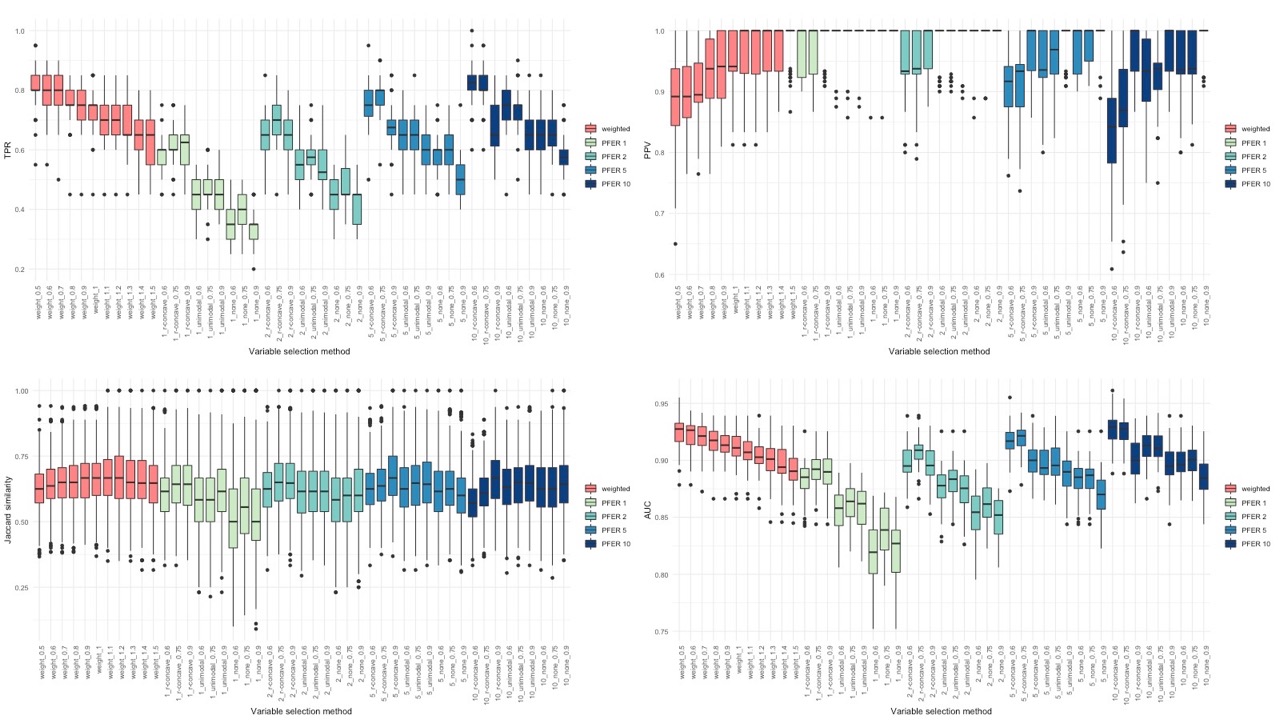


Box plots of scenario 10. $n=500, p=700, p_{signal}=20, \beta_{j}$’s of the signal variables $\sim U\left( 0.5,1.5 \right)$, event prevalence$=0.5$ and the covariance structure of $X=$independent.


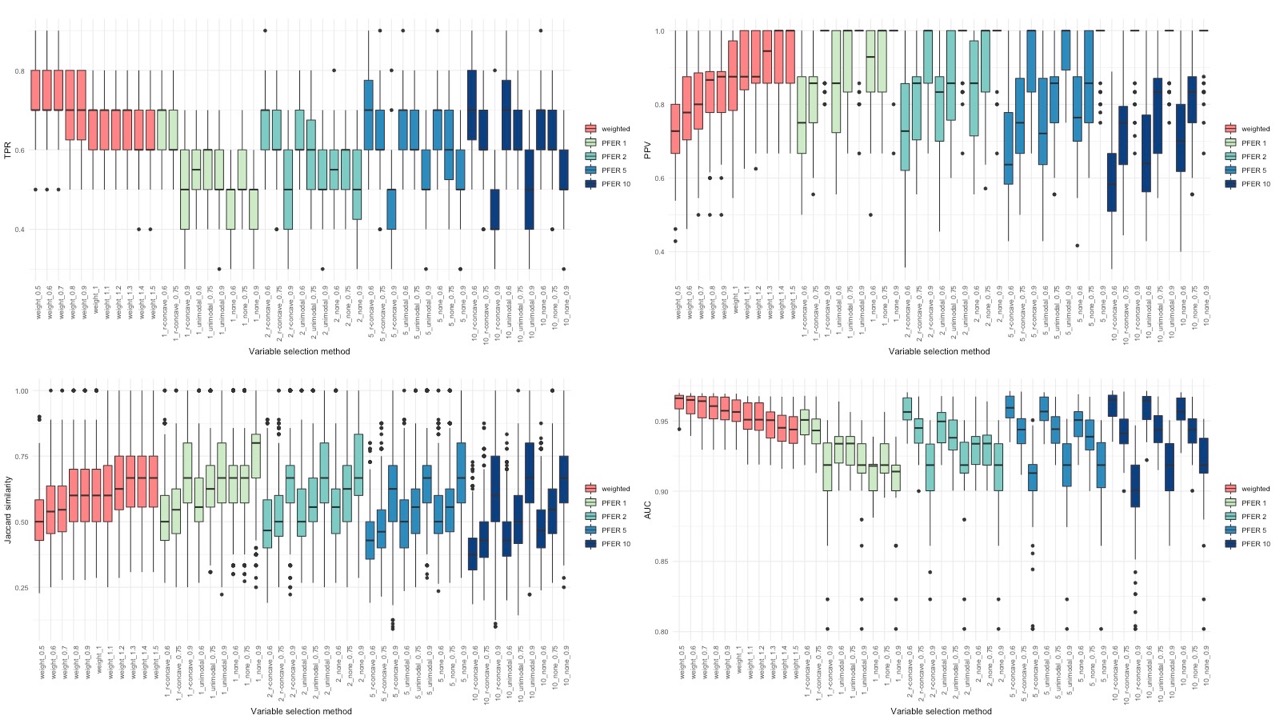


Box plots of scenario 11. $n=500, p=700, p_{signal}=10, \beta_{j}$’s of the signal variables $\sim U\left( -3,3 \right)$, event prevalence$=0.5$ and the covariance structure of $X=$Toeplitz.


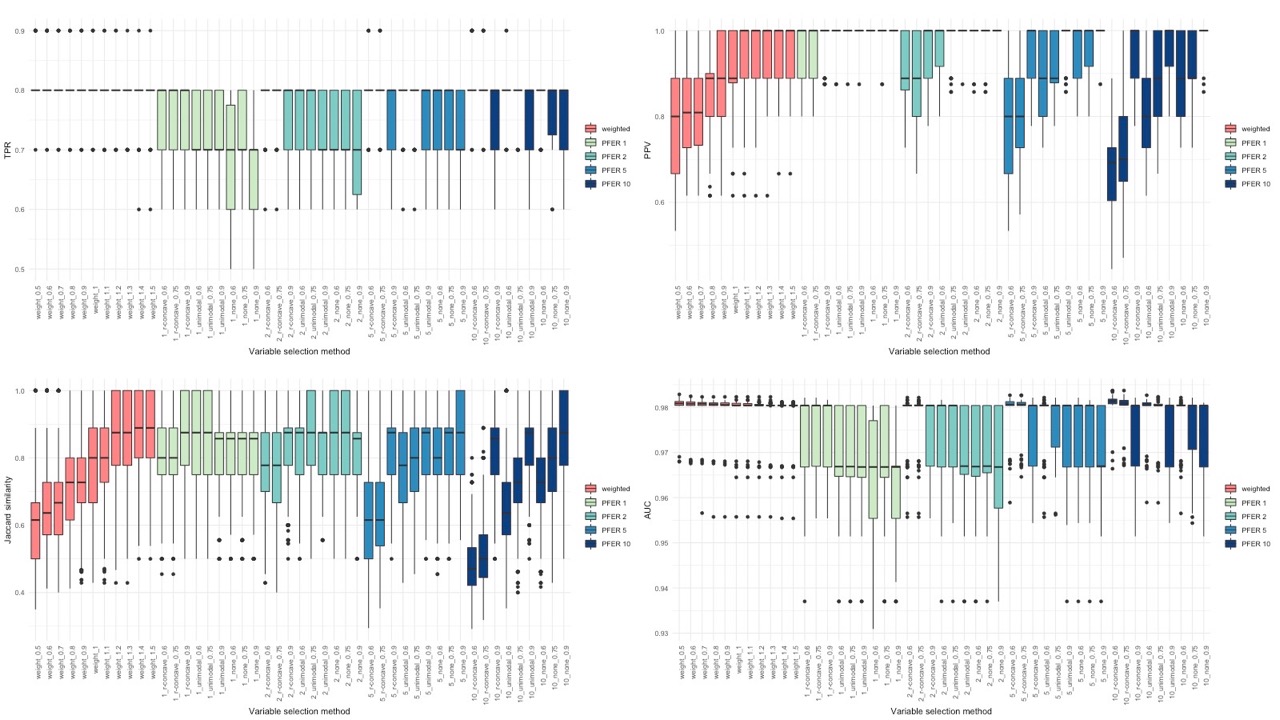


Box plots of scenario 12. $n=500, p=700, p_{signal}=10, \beta_{j}$’s of the signal variables $\sim U\left( -3,3 \right)$, event prevalence$=0.5$ and the covariance structure of $X=$independent.


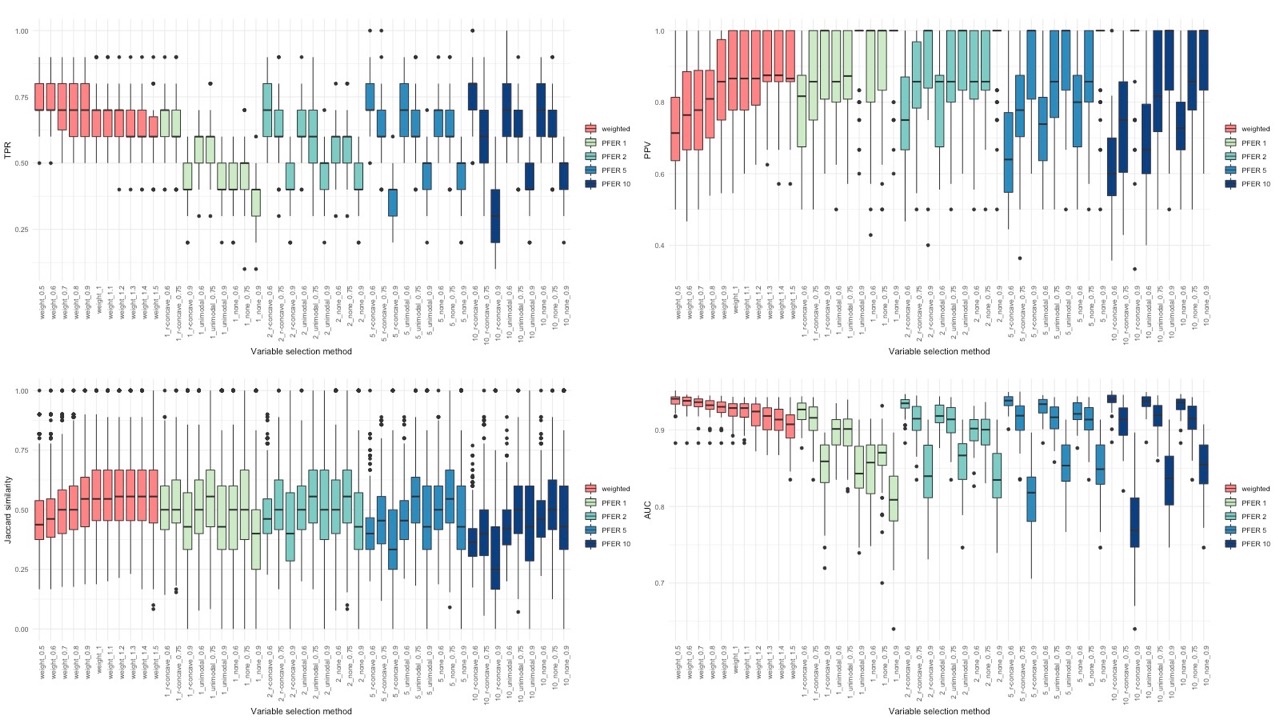


Box plots of scenario 13. $n=500, p=700, p_{signal}=10, \beta_{j}$’s of the signal variables $\sim U\left( 0.5,1.5 \right)$, event prevalence$=0.5$ and the covariance structure of $X=$Toeplitz.


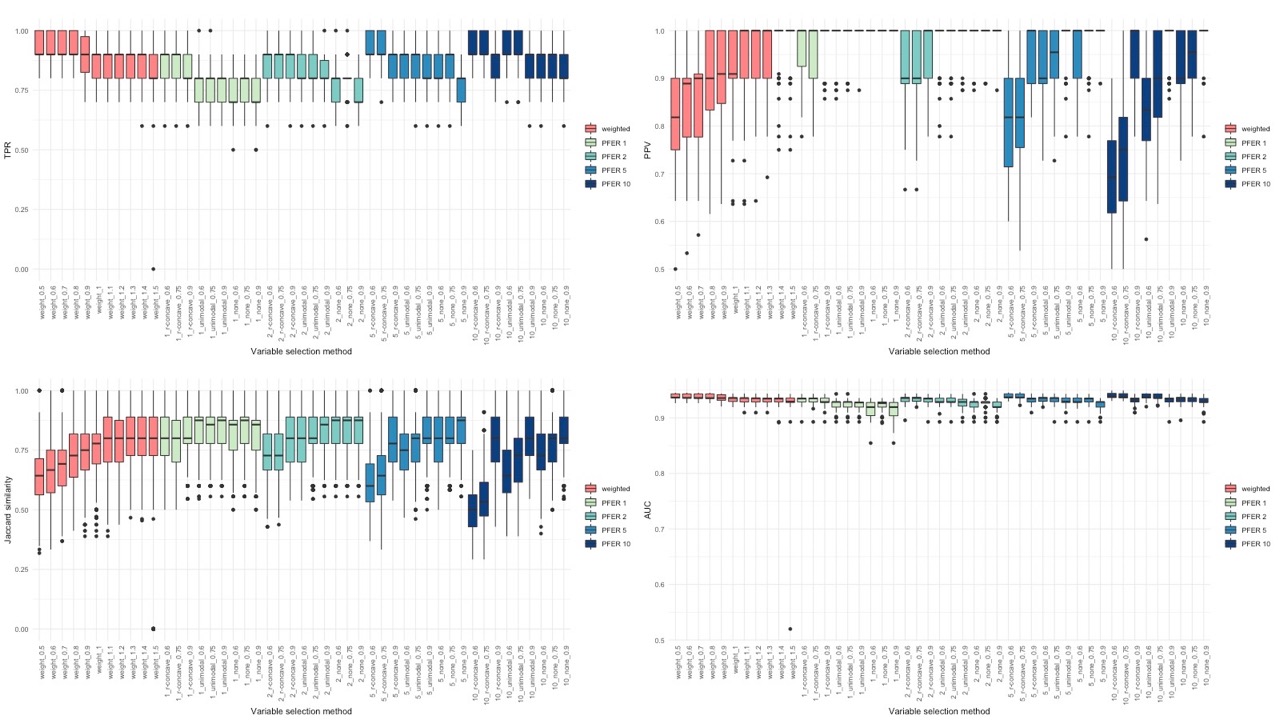


Box plots of scenario 14. $n=500, p=700, p_{signal}=10, \beta_{j}$’s of the signal variables $\sim U\left( 0.5,1.5 \right)$, event prevalence$=0.5$ and the covariance structure of $X=$independent.


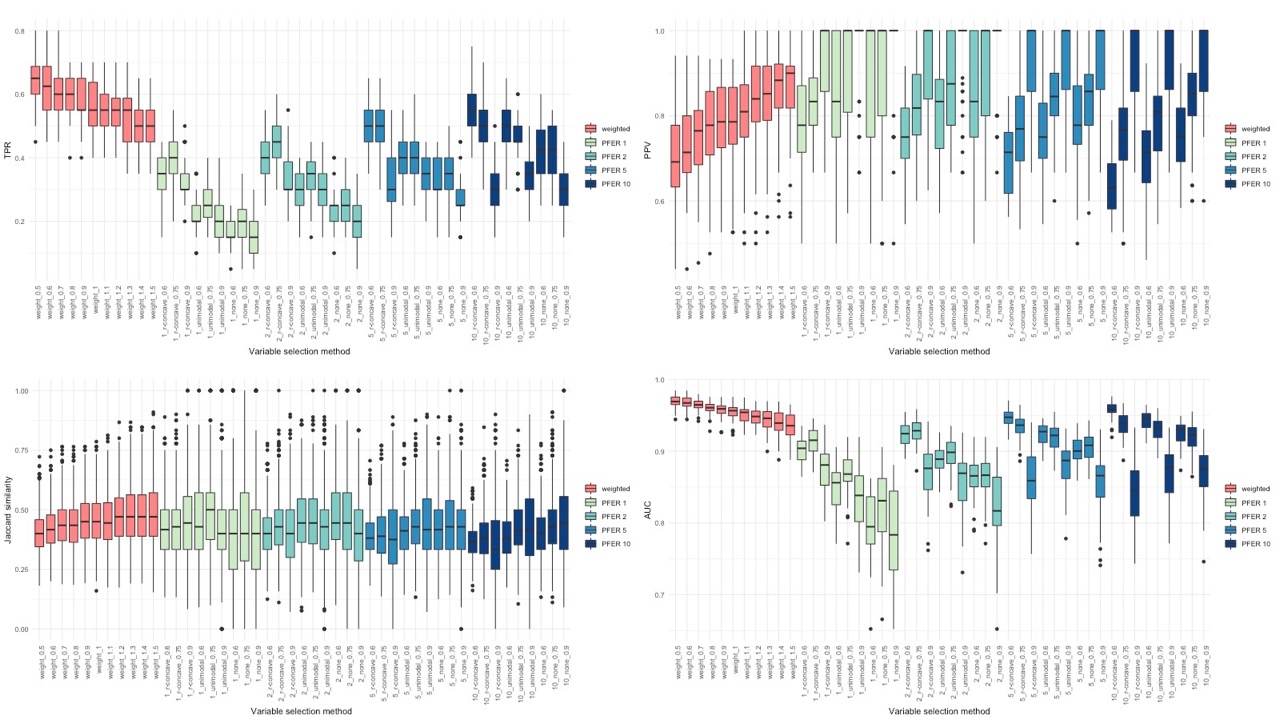


Box plots of scenario 15. $n=500, p=500, p_{signal}=20, \beta_{j}$’s of the signal variables $\sim U\left( -3,3 \right)$, event prevalence$=0.5$ and the covariance structure of $X=$Toeplitz.


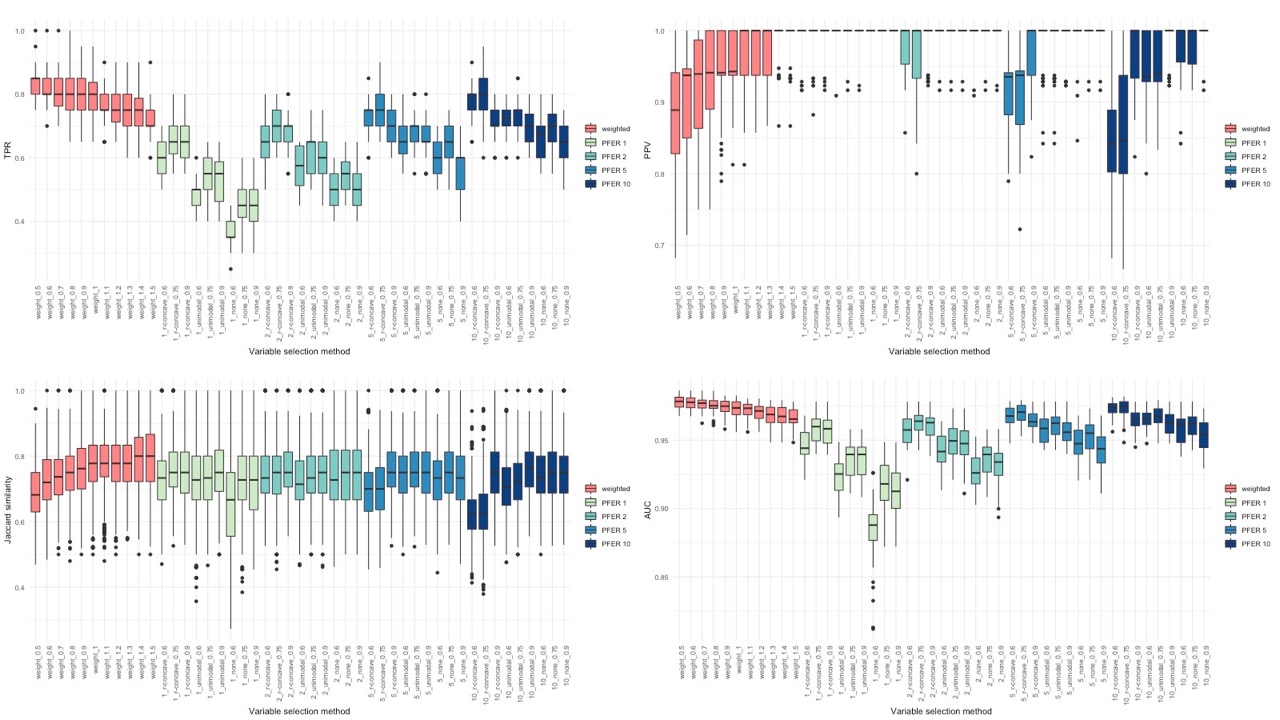


Box plots of scenario 16. $n=500, p=500, p_{signal}=20, \beta_{j}$’s of the signal variables $\sim U\left( -3,3 \right)$, event prevalence$=0.5$ and the covariance structure of $X=$independent.


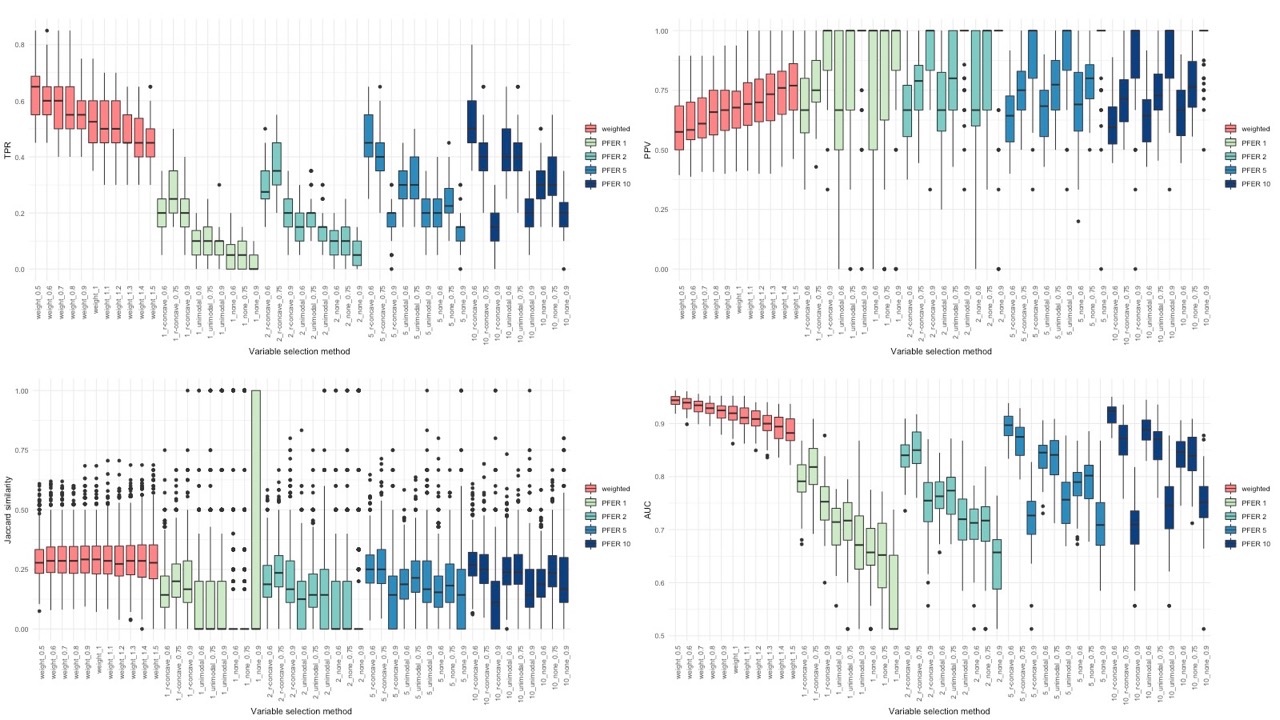


Box plots of scenario 17. $n=500, p=500, p_{signal}=20, \beta_{j}$’s of the signal variables $\sim U\left( 0.5,1.5 \right)$, event prevalence$=0.5$ and the covariance structure of $X=$Toeplitz.


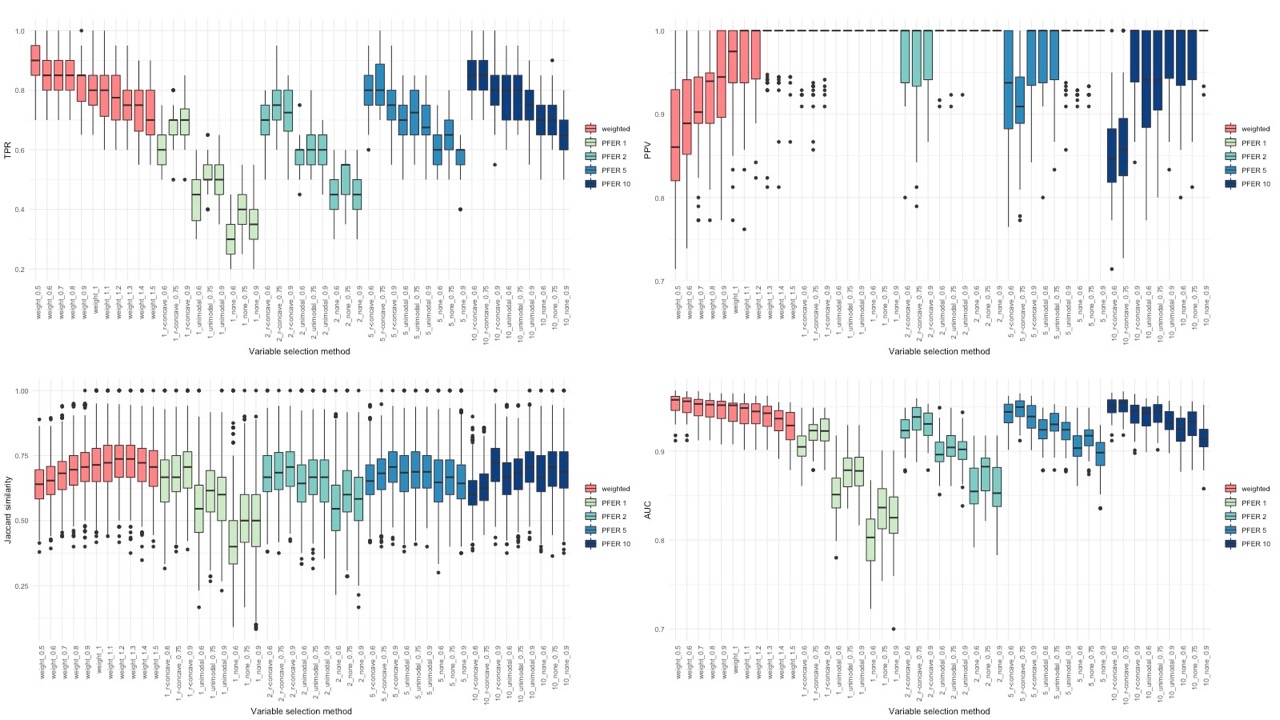


Box plots of scenario 18. $n=500, p=500, p_{signal}=20, \beta_{j}$’s of the signal variables $\sim U\left( 0.5,1.5 \right)$, event prevalence$=0.5$ and the covariance structure of $X=$independent.


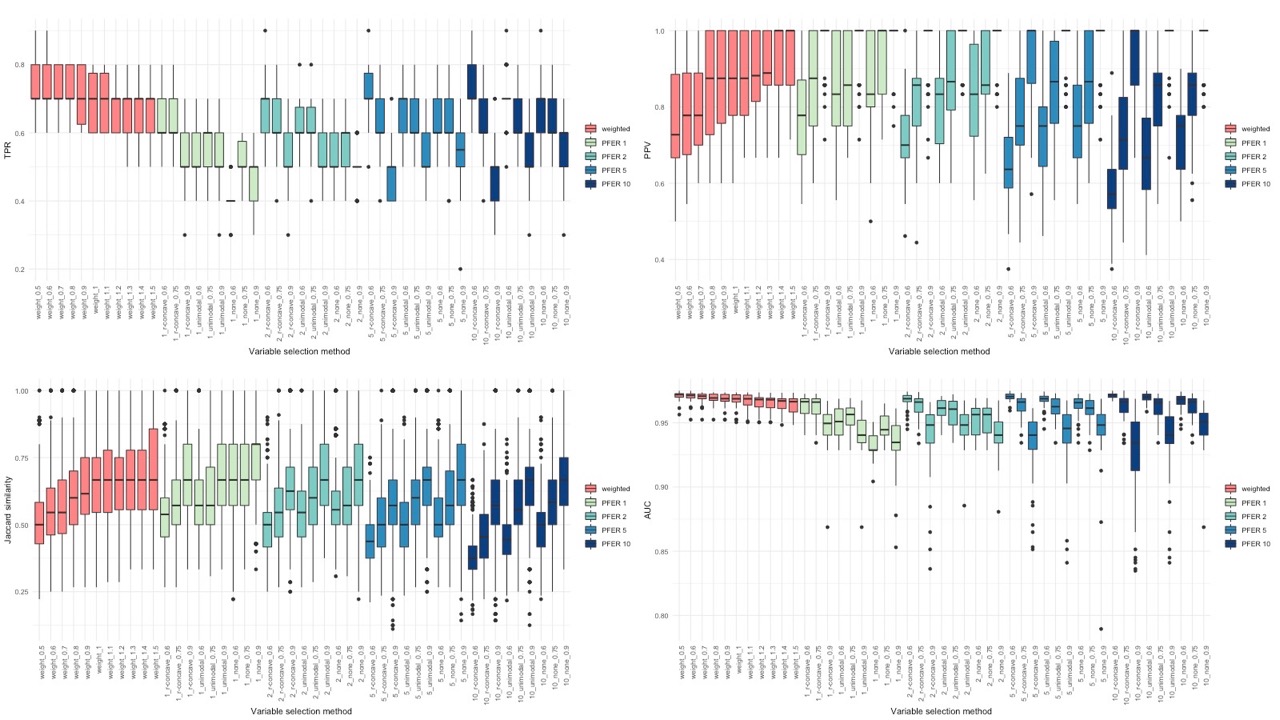


Box plots of scenario 19. $n=500, p=500, p_{signal}=10, \beta_{j}$’s of the signal variables $\sim U\left( -3,3 \right)$, event prevalence$=0.5$ and the covariance structure of $X=$Toeplitz.


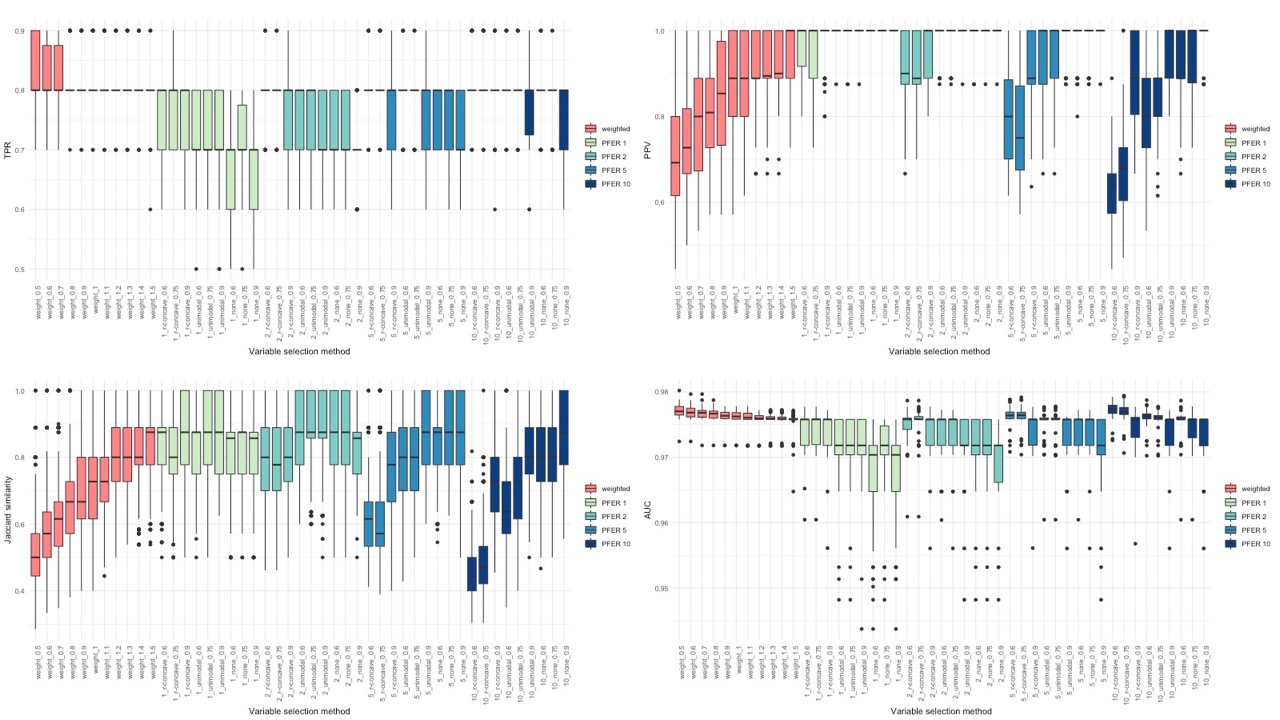


Box plots of scenario 20. $n=500, p=500, p_{signal}=10, \beta_{j}$’s of the signal variables $\sim U\left( -3,3 \right)$, event prevalence$=0.5$ and the covariance structure of $X=$independent.


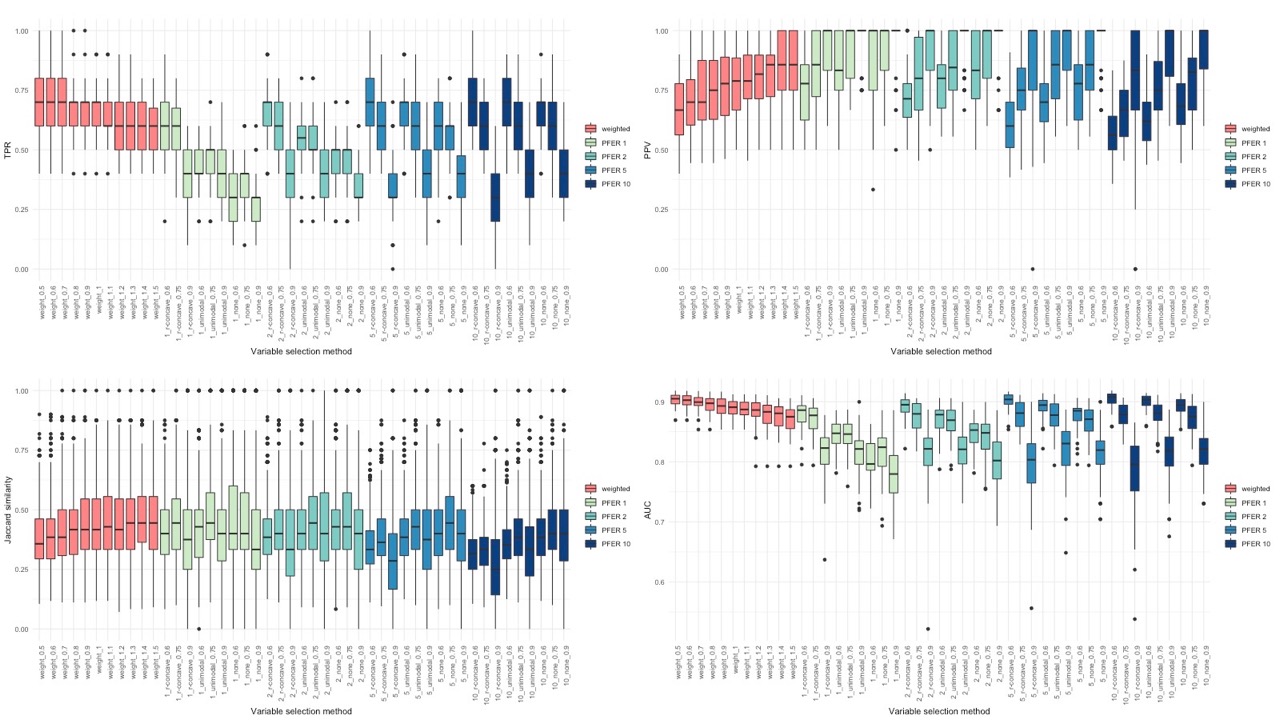


Box plots of scenario 21. $n=500, p=500, p_{signal}=10, \beta_{j}$’s of the signal variables $\sim U\left( 0.5,1.5 \right)$, event prevalence$=0.5$ and the covariance structure of $X=$Toeplitz.


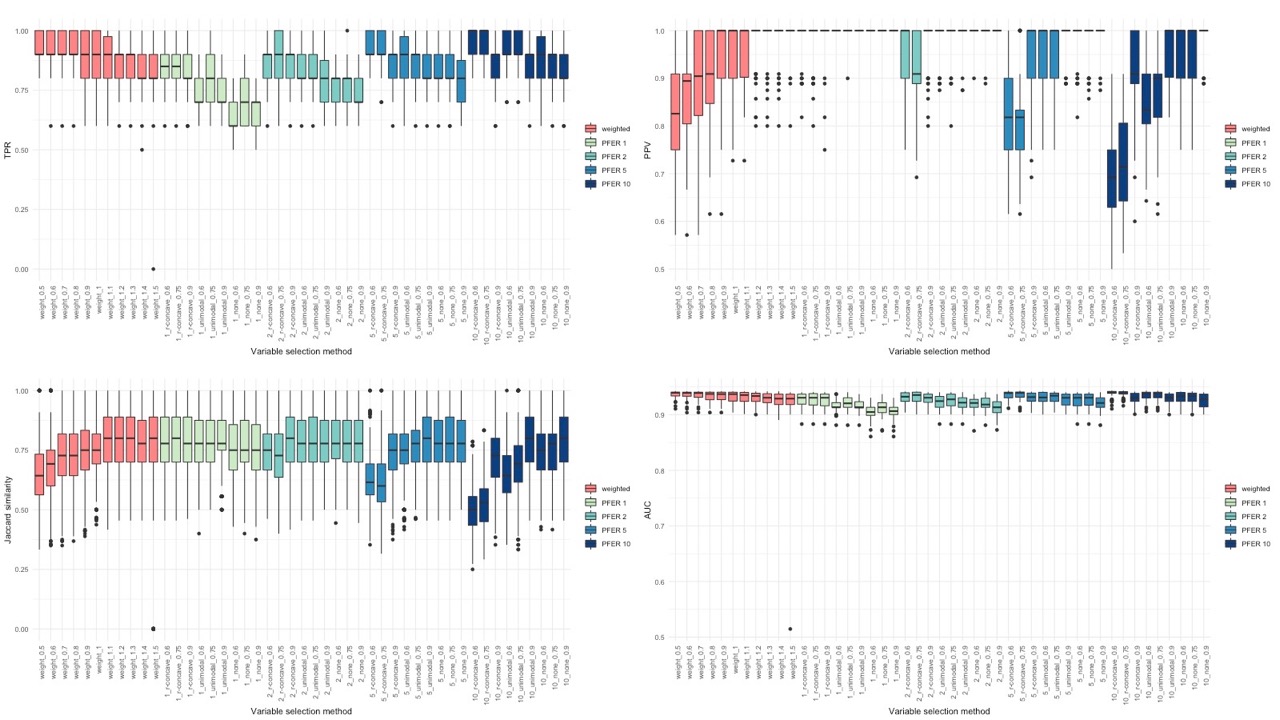


Box plots of scenario 22. $n=500, p=500, p_{signal}=10, \beta_{j}$’s of the signal variables $\sim U\left( 0.5,1.5 \right)$, event prevalence$=0.5$ and the covariance structure of $X=$independent.


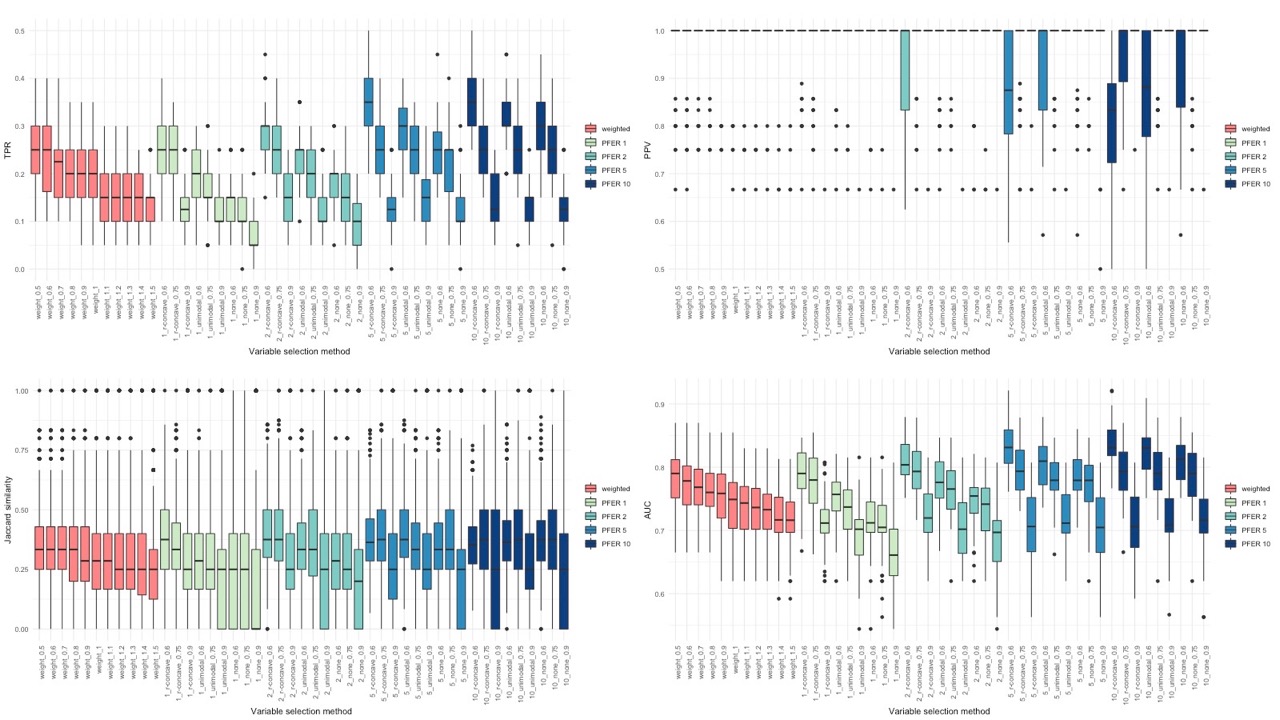


Box plots of scenario 23. $n=200, p=1000, p_{signal}=20, \beta_{j}$’s of the signal variables $\sim U\left( -3,3 \right)$, event prevalence$=0.5$ and the covariance structure of $X=$independent.


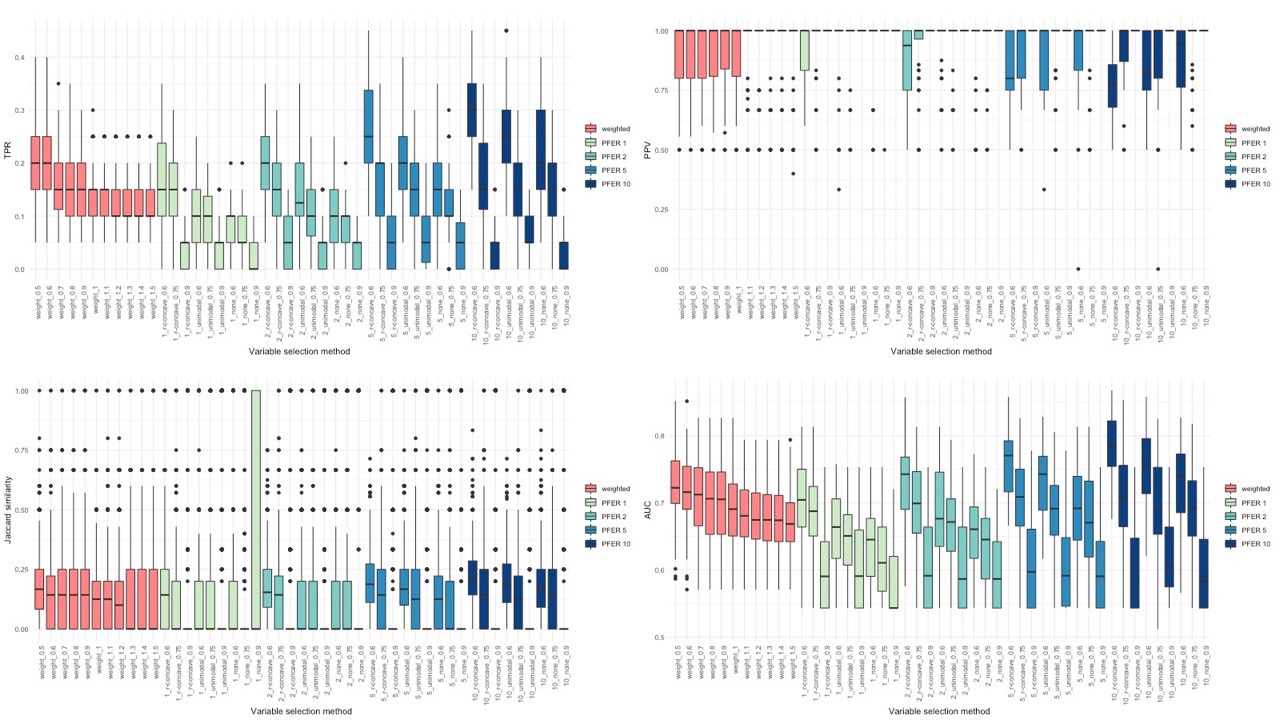


Box plots of scenario 24. $n=200, p=1000, p_{signal}=20, \beta_{j}$’s of the signal variables $\sim U\left( 0.5,1.5 \right)$, event prevalence$=0.5$ and the covariance structure of $X=$independent.


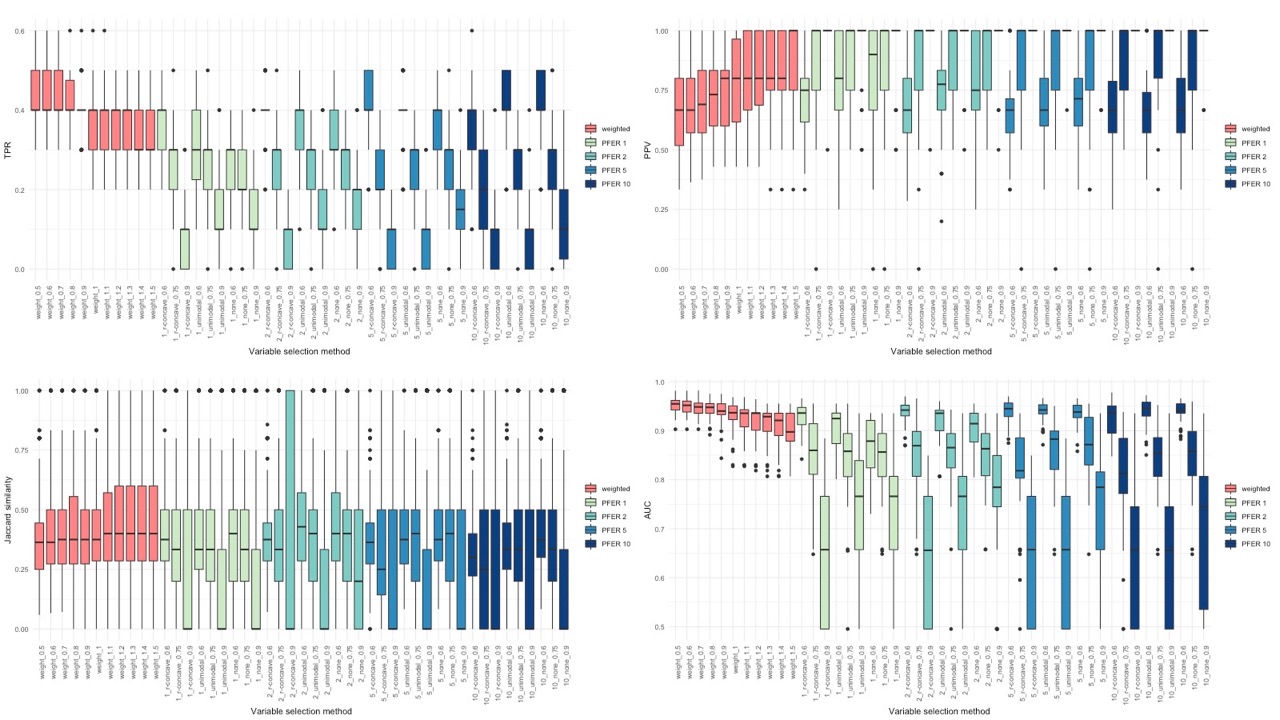


Box plots of scenario 25. $n=200, p=1000, p_{signal}=10, \beta_{j}$’s of the signal variables $\sim U\left( -3,3 \right)$, event prevalence$=0.5$ and the covariance structure of $X=$Toeplitz.


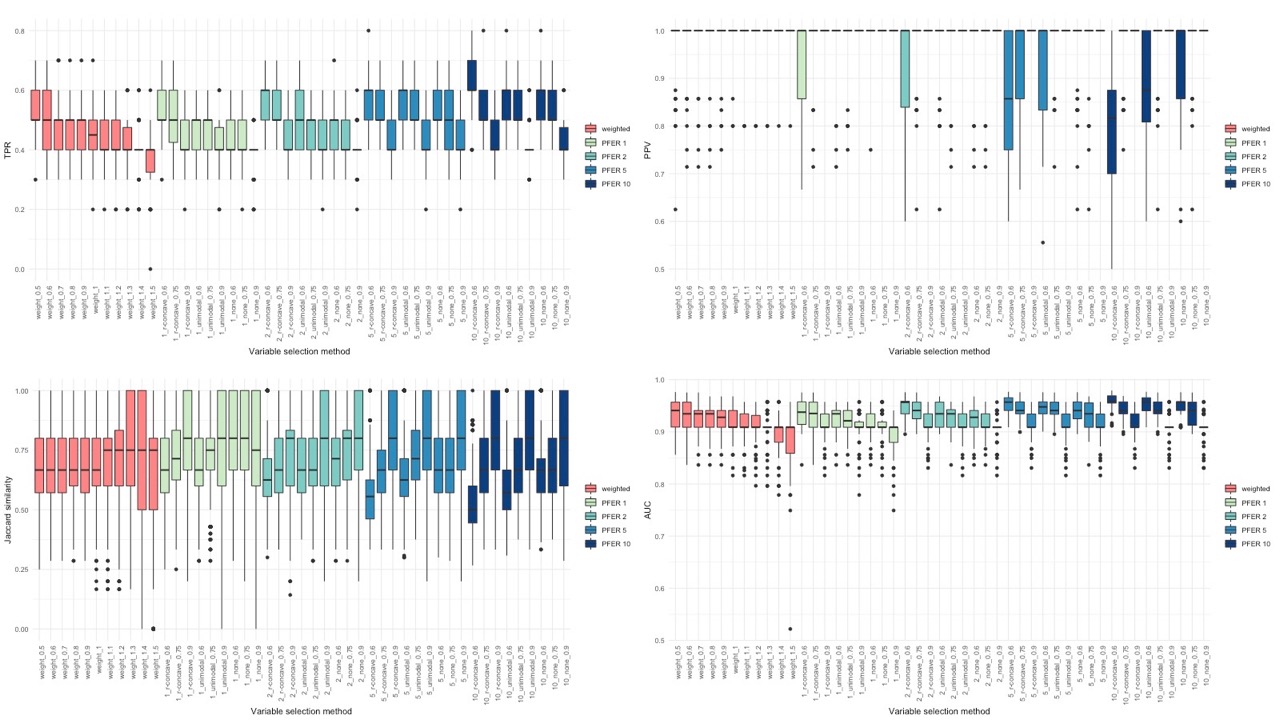


Box plots of scenario 26. $n=200, p=1000, p_{signal}=10, \beta_{j}$’s of the signal variables $\sim U\left( -3,3 \right)$, event prevalence$=0.5$ and the covariance structure of $X=$independent.


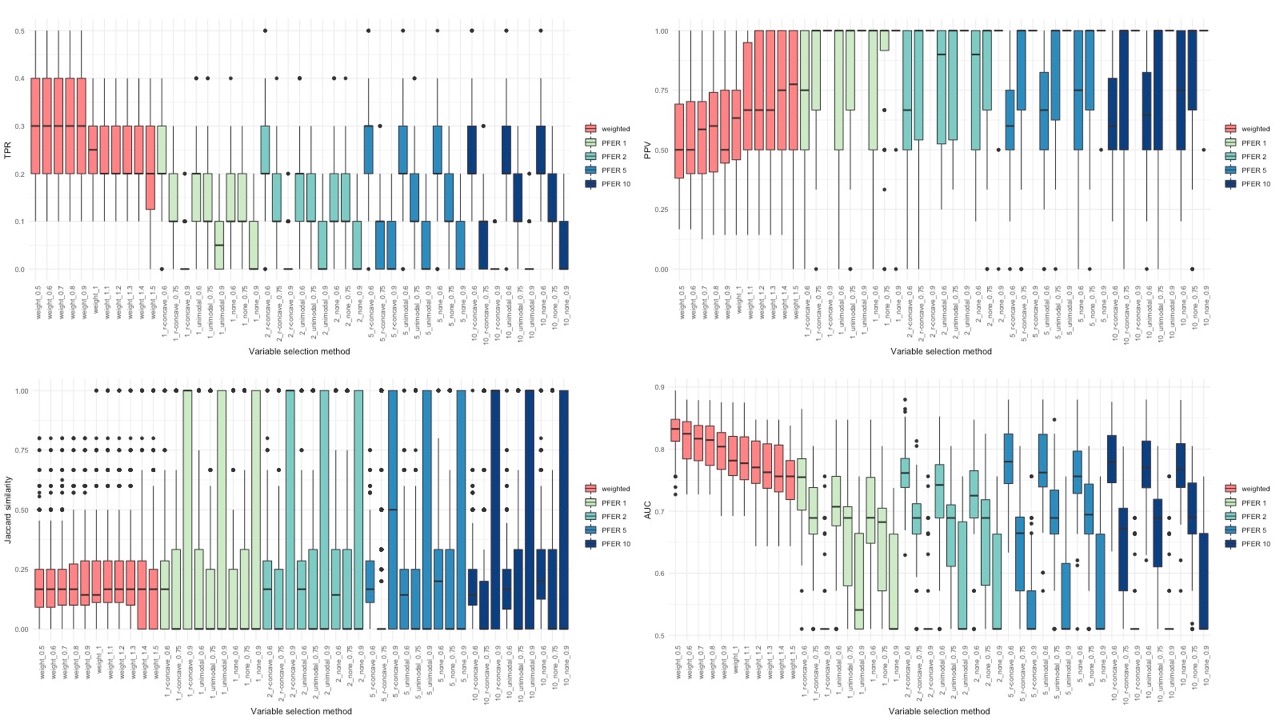


Box plots of scenario 27. $n=200, p=1000, p_{signal}=10, \beta_{j}$’s of the signal variables $\sim U\left( 0.5,1.5 \right)$, event prevalence$=0.5$ and the covariance structure of $X=$Toeplitz.


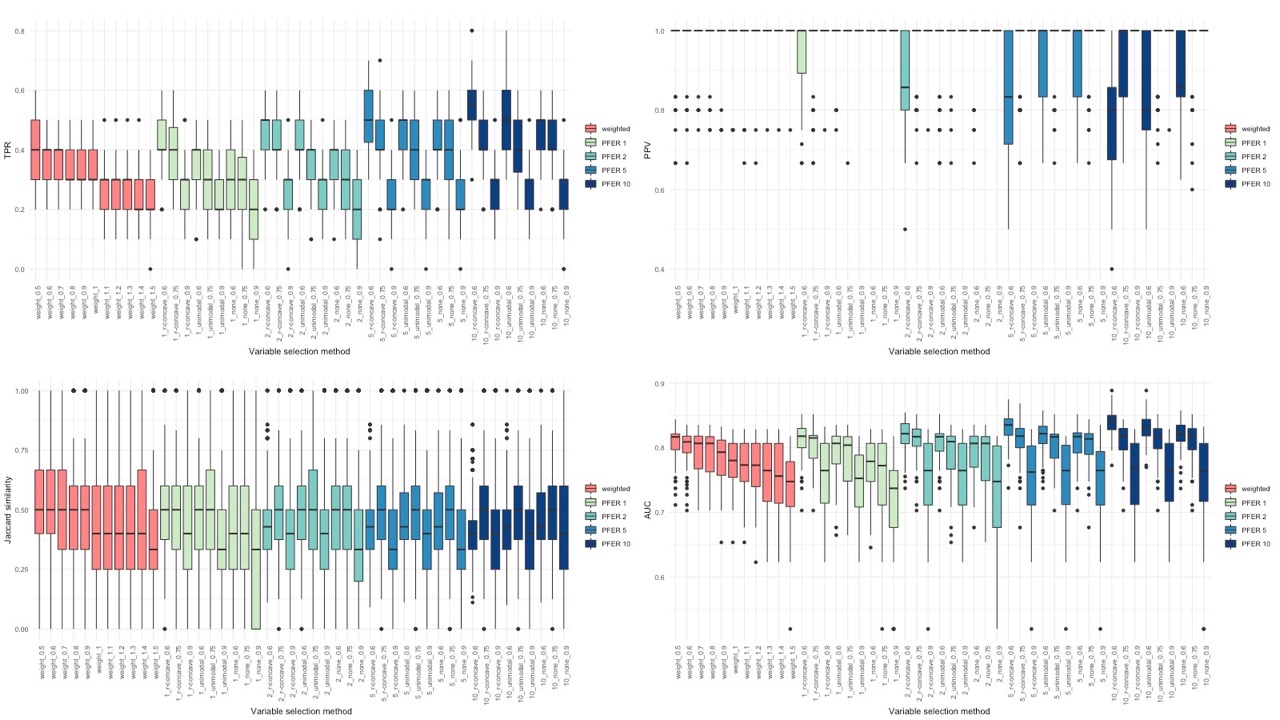


Box plots of scenario 28. $n=200, p=1000, p_{signal}=10, \beta_{j}$’s of the signal variables $\sim U\left( 0.5,1.5 \right)$, event prevalence$=0.5$ and the covariance structure of $X=$independent.


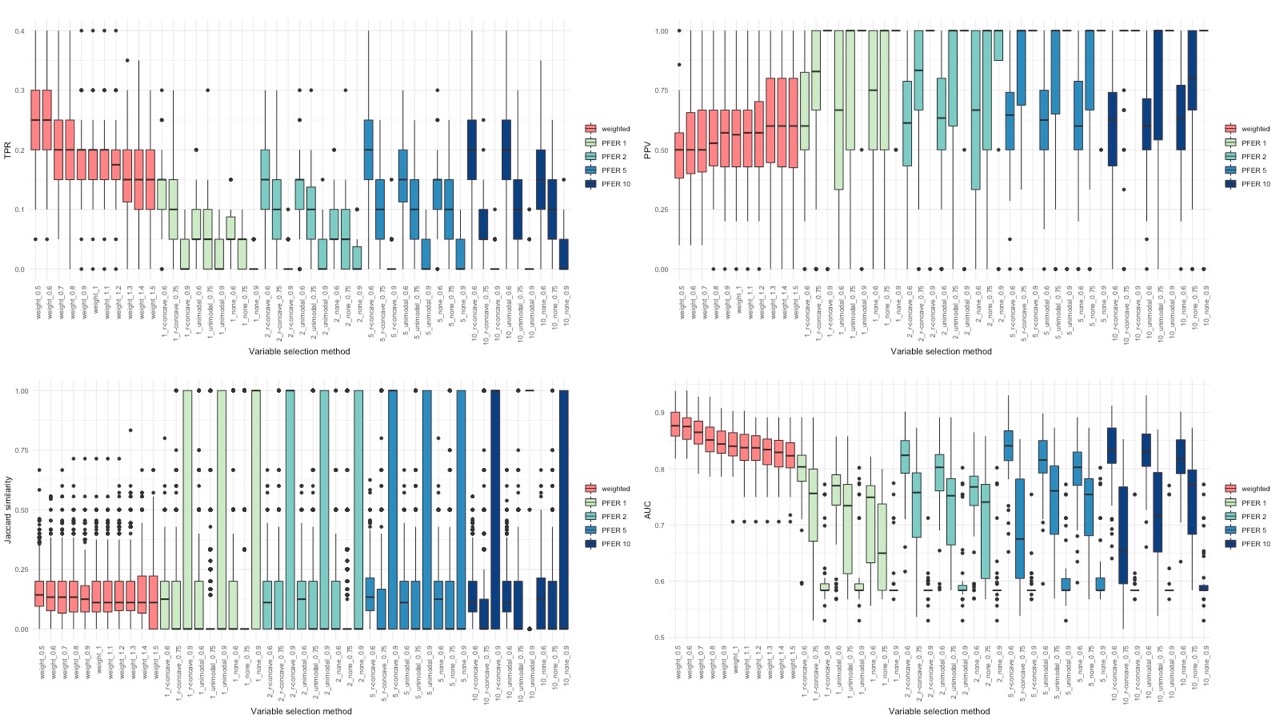


Box plots of scenario 29. $n=200, p=700, p_{signal}=20, \beta_{j}$’s of the signal variables $\sim U\left( -3,3 \right)$, event prevalence$=0.5$ and the covariance structure of $X=$Toeplitz.


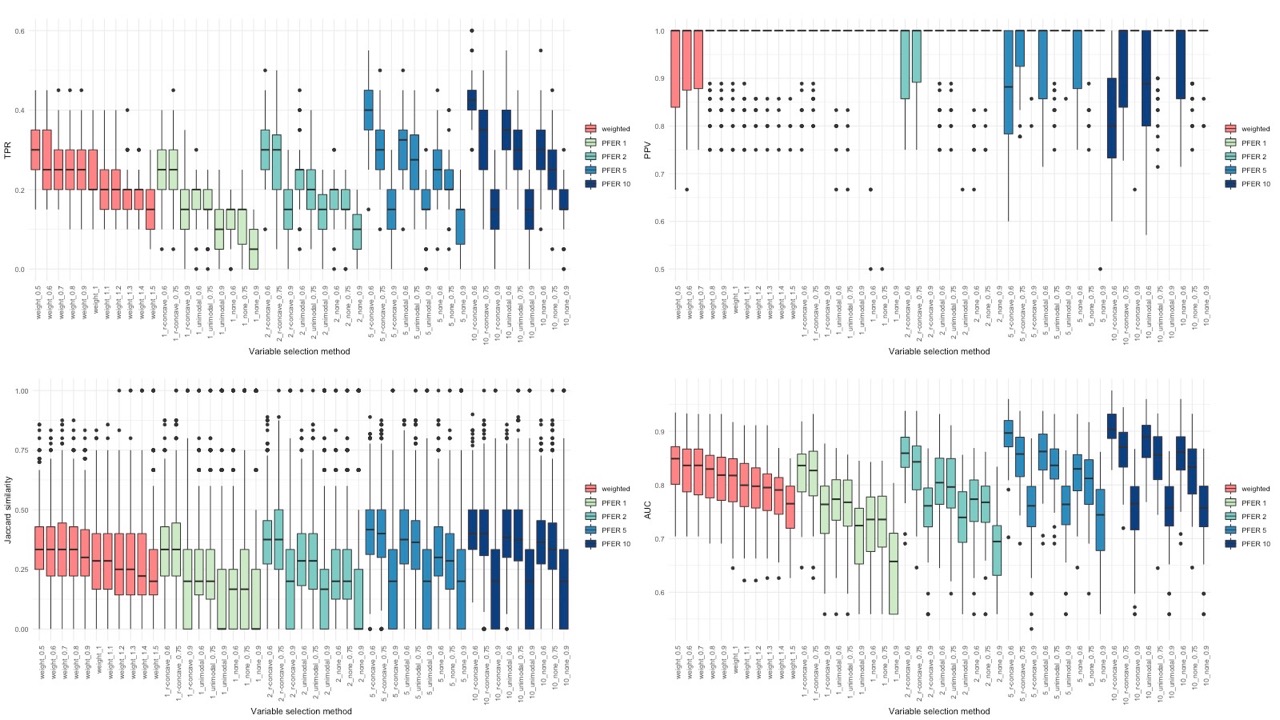


Box plots of scenario 30. $n=200, p=700, p_{signal}=20, \beta_{j}$’s of the signal variables $\sim U\left( -3,3 \right)$, event prevalence$=0.5$ and the covariance structure of $X=$independent.


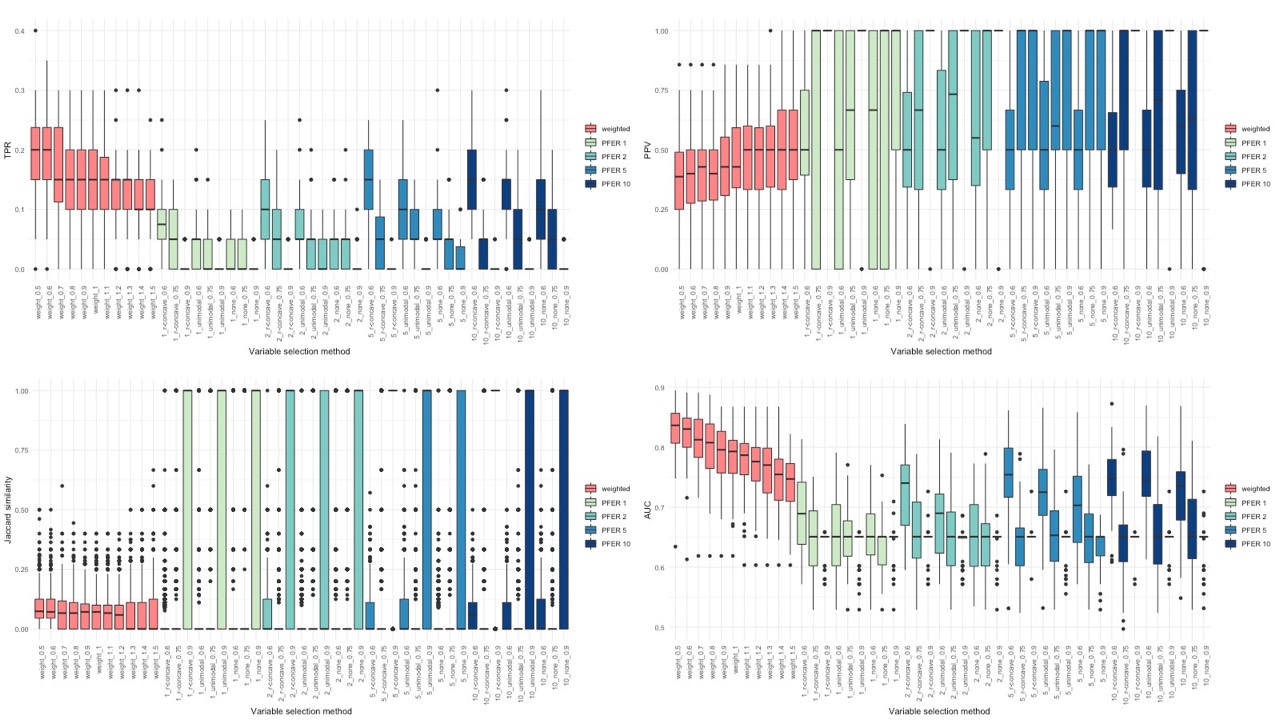


Box plots of scenario 31. $n=200, p=700, p_{signal}=20, \beta_{j}$’s of the signal variables $\sim U\left( 0.5,1.5 \right)$, event prevalence$=0.5$ and the covariance structure of $X=$Toeplitz.


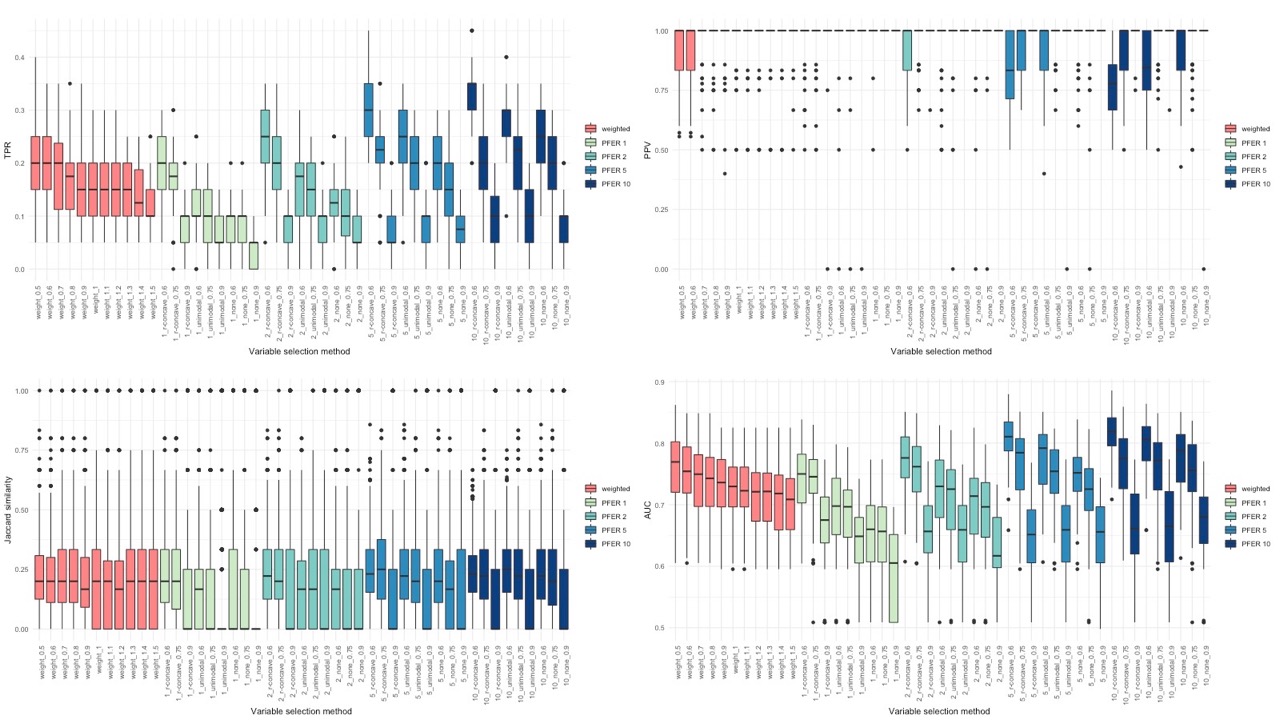


Box plots of scenario 32. $n=200, p=700, p_{signal}=20, \beta_{j}$’s of the signal variables $\sim U\left( 0.5,1.5 \right)$, event prevalence$=0.5$ and the covariance structure of $X=$independent.


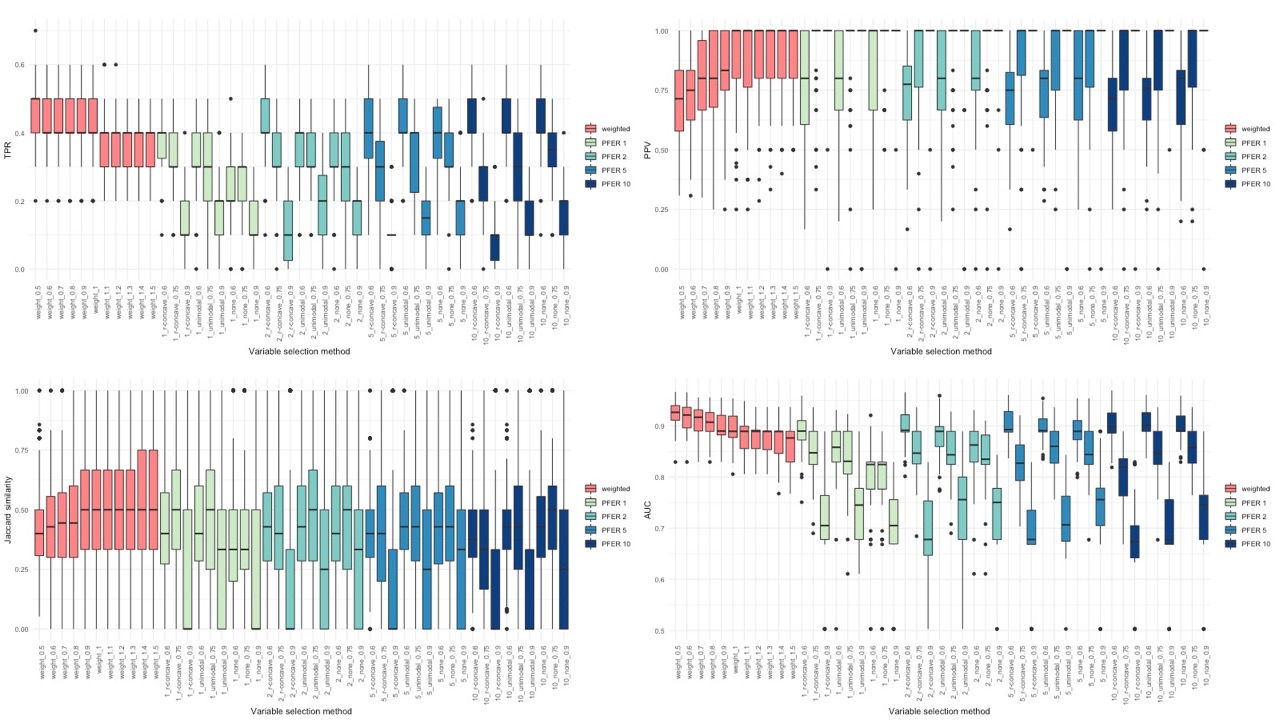


Box plots of scenario 33. $n=200, p=700, p_{signal}=10, \beta_{j}$’s of the signal variables $\sim U\left( -3,3 \right)$, event prevalence$=0.5$ and the covariance structure of $X=$Toeplitz.


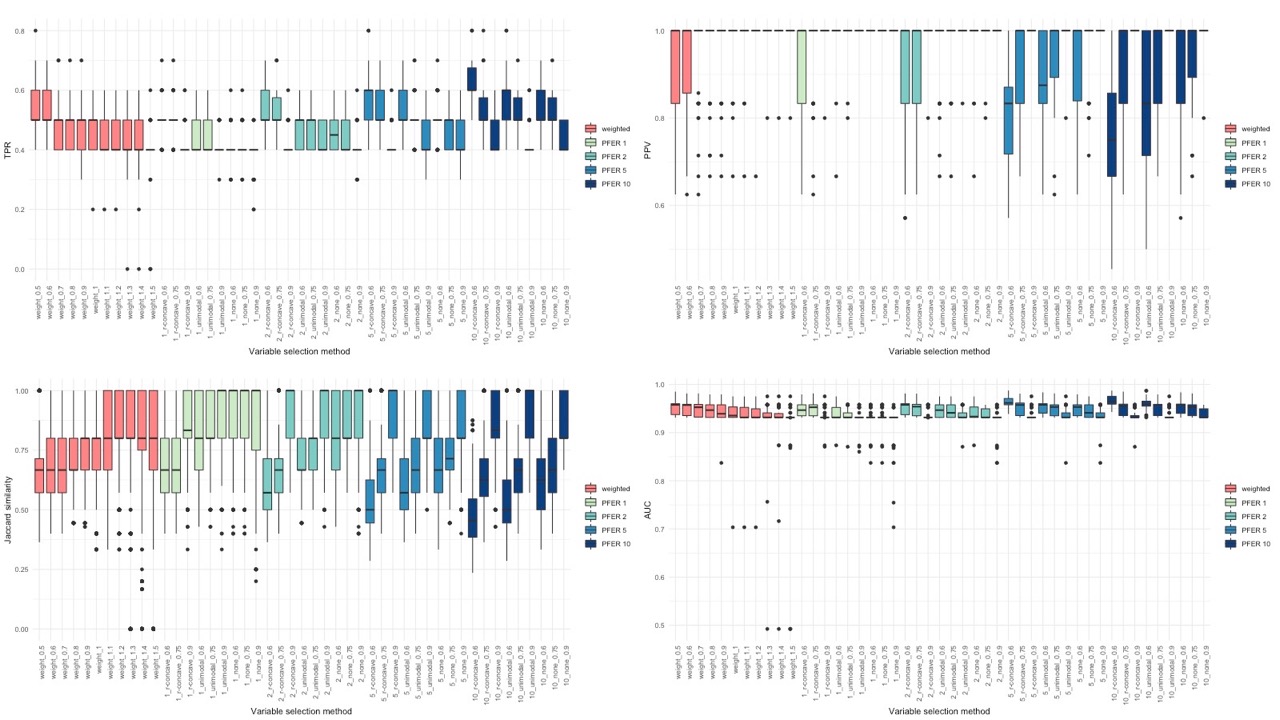


Box plots of scenario 34. $n=200, p=700, p_{signal}=10, \beta_{j}$’s of the signal variables $\sim U\left( -3,3 \right)$, event prevalence$=0.5$ and the covariance structure of $X=$independent.


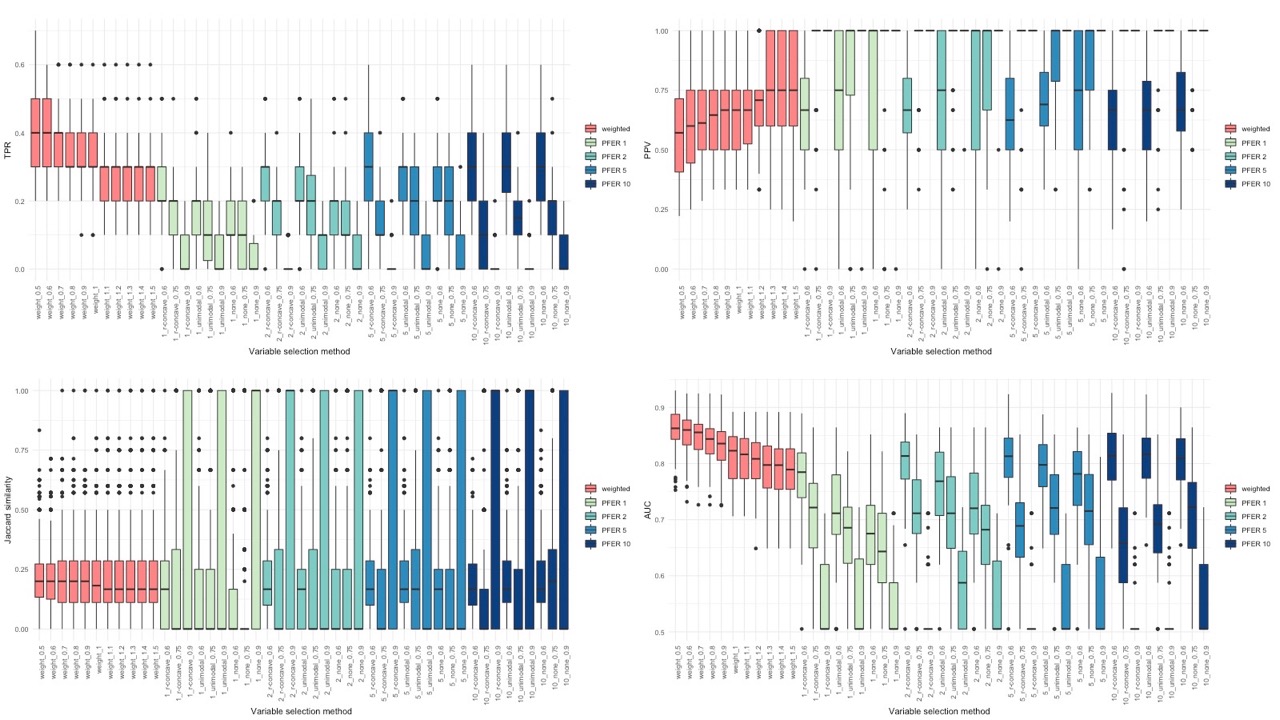


Box plots of scenario 35. $n=200, p=700, p_{signal}=10, \beta_{j}$’s of the signal variables $\sim U\left( 0.5,1.5 \right)$, event prevalence$=0.5$ and the covariance structure of $X=$Toeplitz.


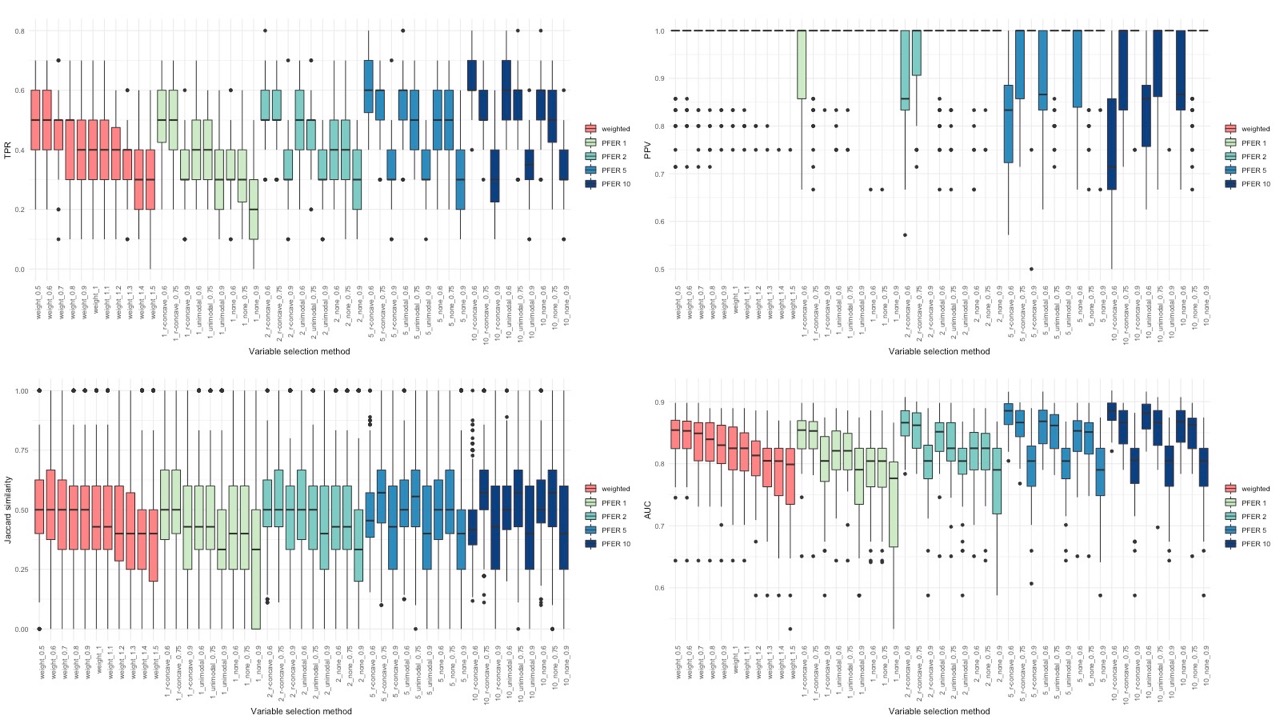


Box plots of scenario 36. $n=200, p=700, p_{signal}=10, \beta_{j}$’s of the signal variables $\sim U\left( 0.5,1.5 \right)$, event prevalence$=0.5$ and the covariance structure of $X=$independent.


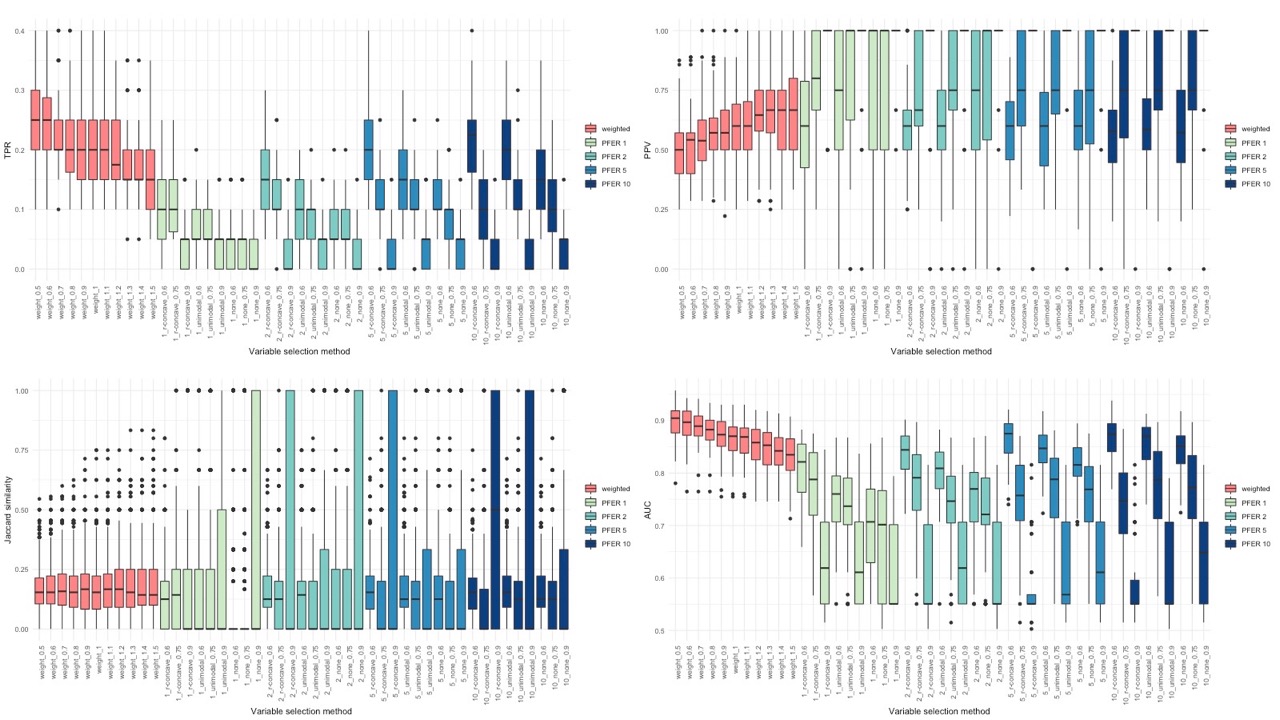


Box plots of scenario 37. $n=200, p=500, p_{signal}=20, \beta_{j}$’s of the signal variables $\sim U\left( -3,3 \right)$, event prevalence$=0.5$ and the covariance structure of $X=$Toeplitz.


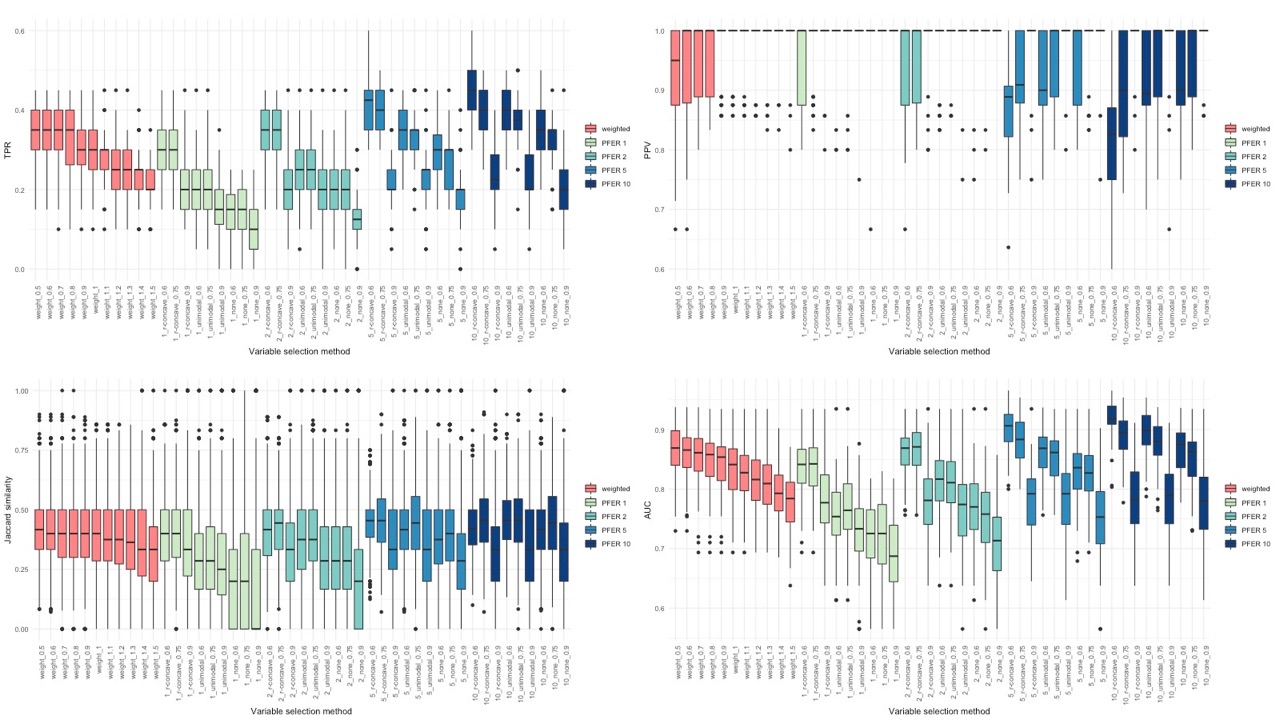


Box plots of scenario 38. $n=200, p=500, p_{signal}=20, \beta_{j}$’s of the signal variables $\sim U\left( -3,3 \right)$, event prevalence$=0.5$ and the covariance structure of $X=$independent.


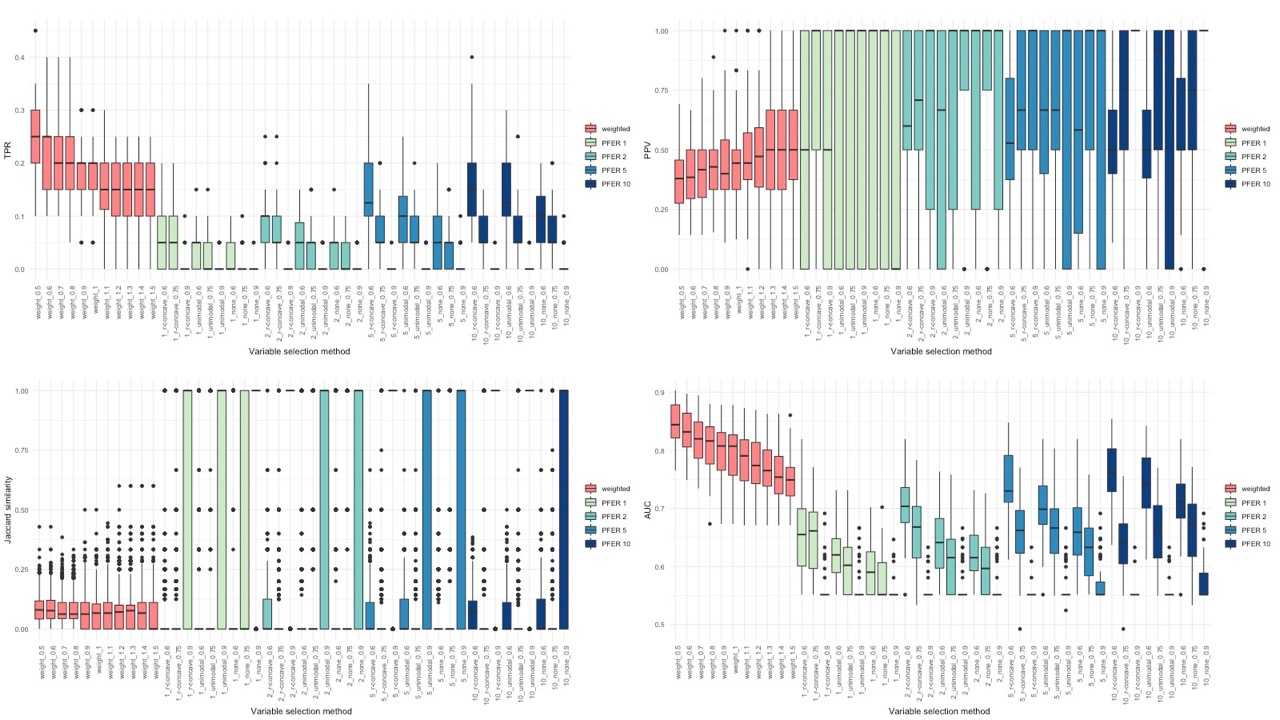


Box plots of scenario 39. $n=200, p=500, p_{signal}=20, \beta_{j}$’s of the signal variables $\sim U\left( 0.5,1.5 \right)$, event prevalence$=0.5$ and the covariance structure of $X=$Toeplitz.


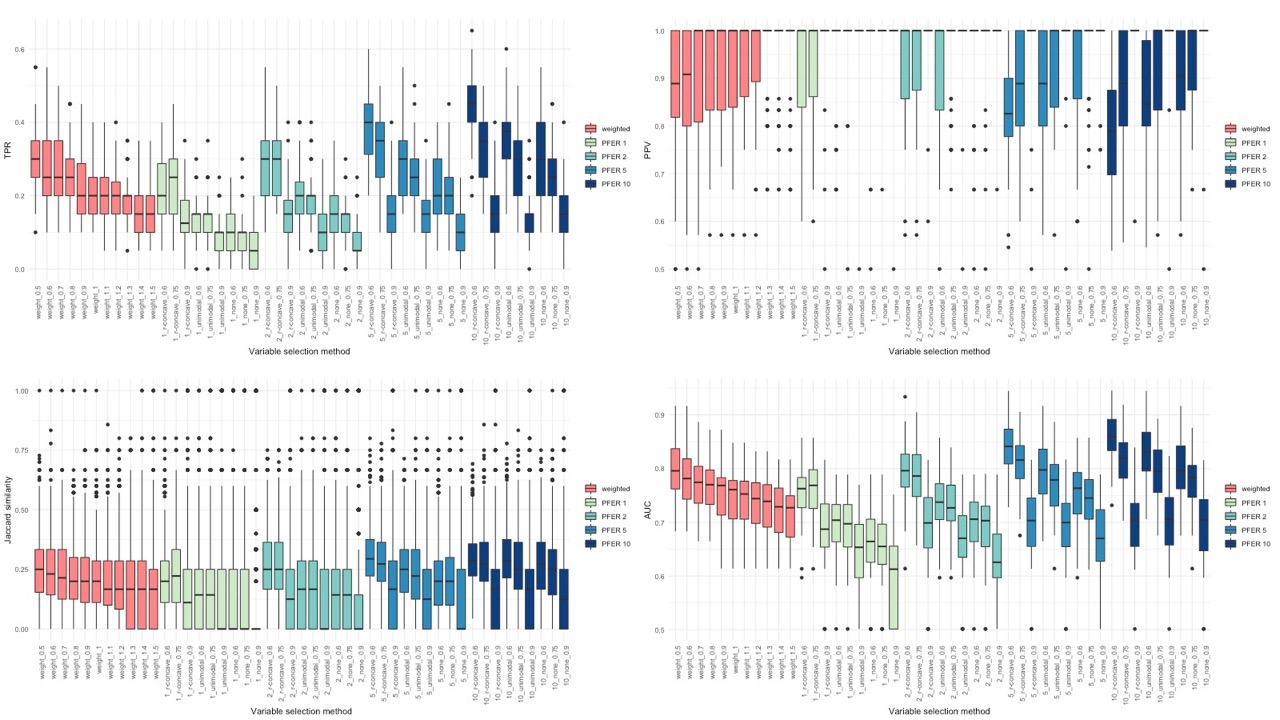


Box plots of scenario 40. $n=200, p=500, p_{signal}=20, \beta_{j}$’s of the signal variables $\sim U\left( 0.5,1.5 \right)$, event prevalence$=0.5$ and the covariance structure of $X=$independent.


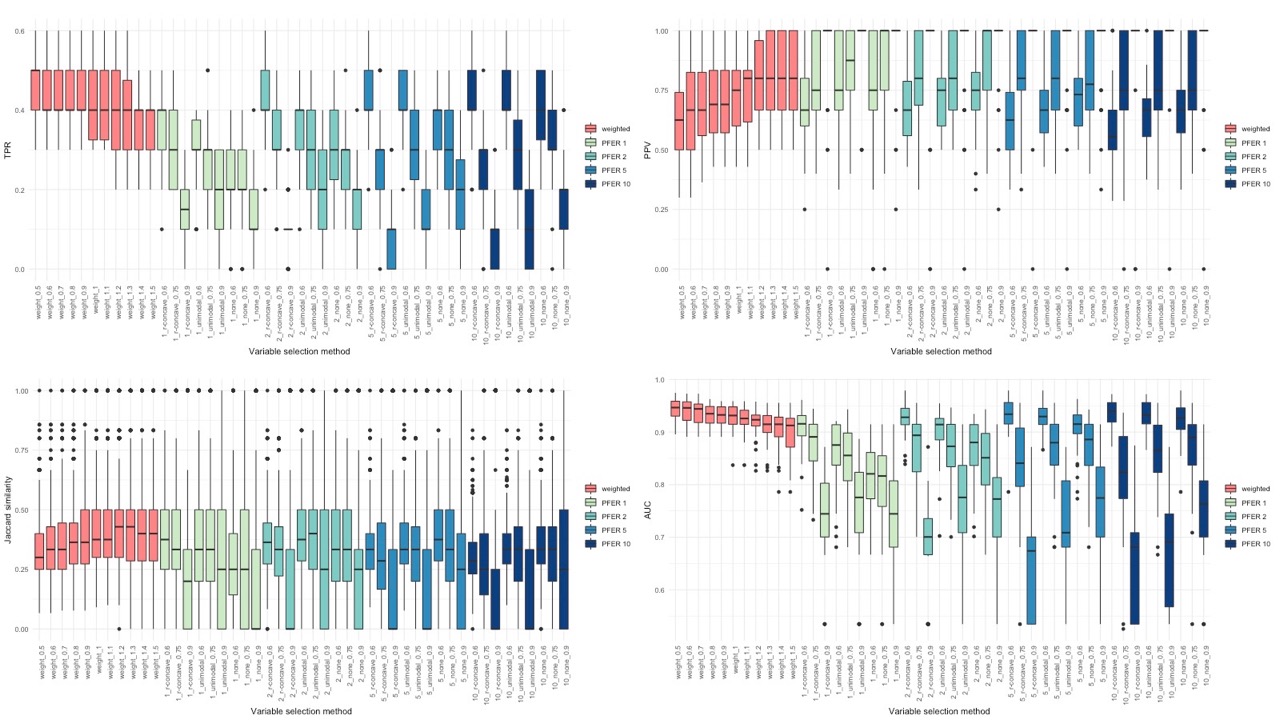


Box plots of scenario 41. $n=200, p=500, p_{signal}=10, \beta_{j}$’s of the signal variables $\sim U\left( -3,3 \right)$, event prevalence$=0.5$ and the covariance structure of $X=$Toeplitz.


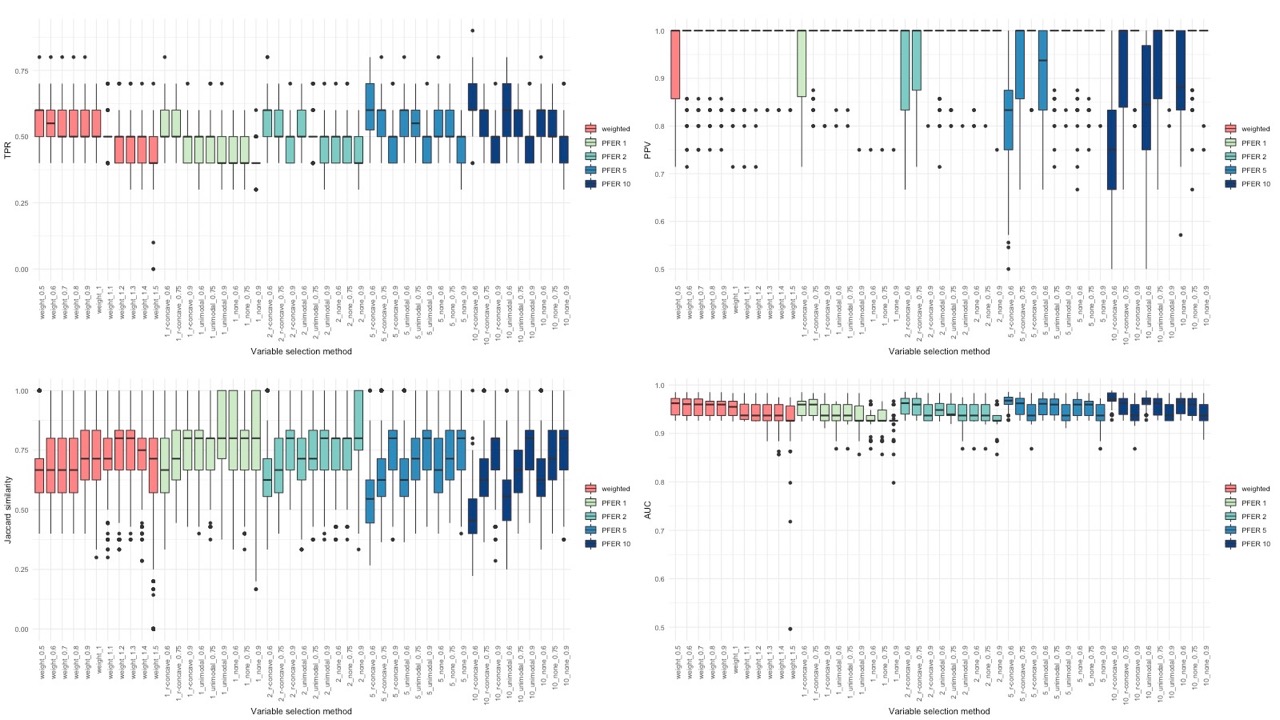


Box plots of scenario 42. $n=200, p=500, p_{signal}=10, \beta_{j}$’s of the signal variables $\sim U\left( -3,3 \right)$, event prevalence$=0.5$ and the covariance structure of $X=$independent.


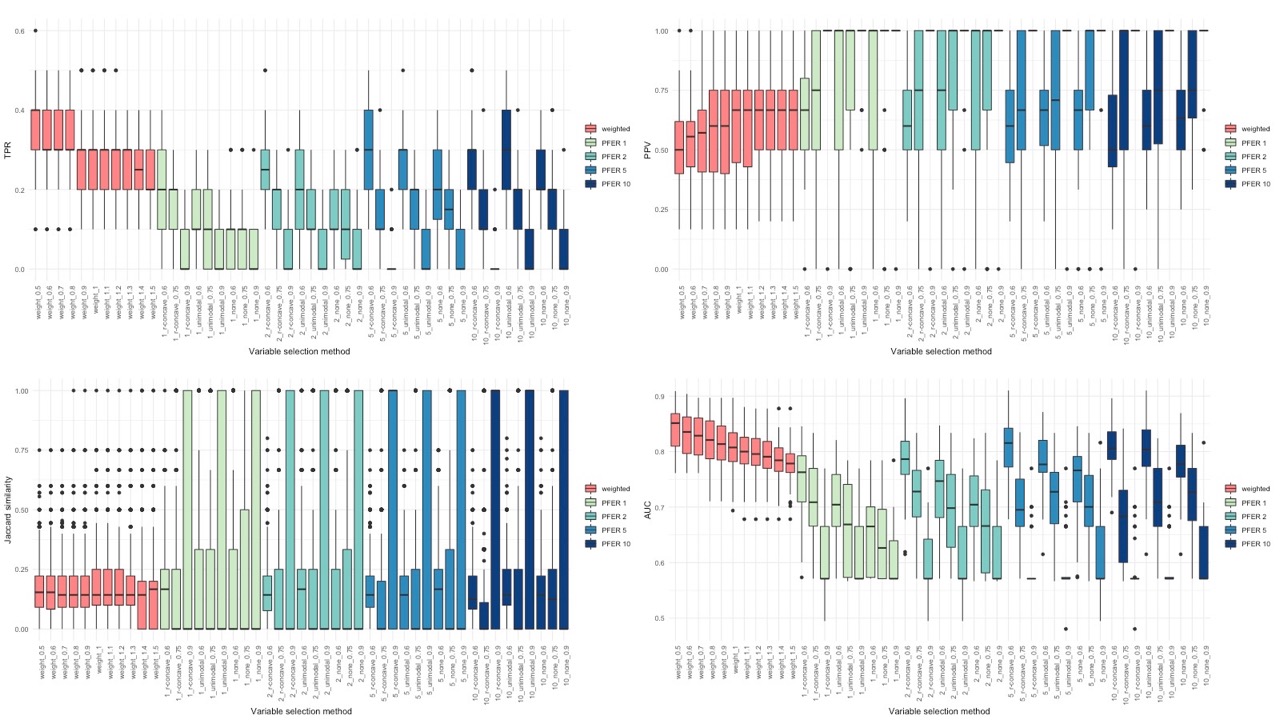


Box plots of scenario 43. $n=200, p=500, p_{signal}=10, \beta_{j}$’s of the signal variables $\sim U\left( 0.5,1.5 \right)$, event prevalence$=0.5$ and the covariance structure of $X=$Toeplitz.


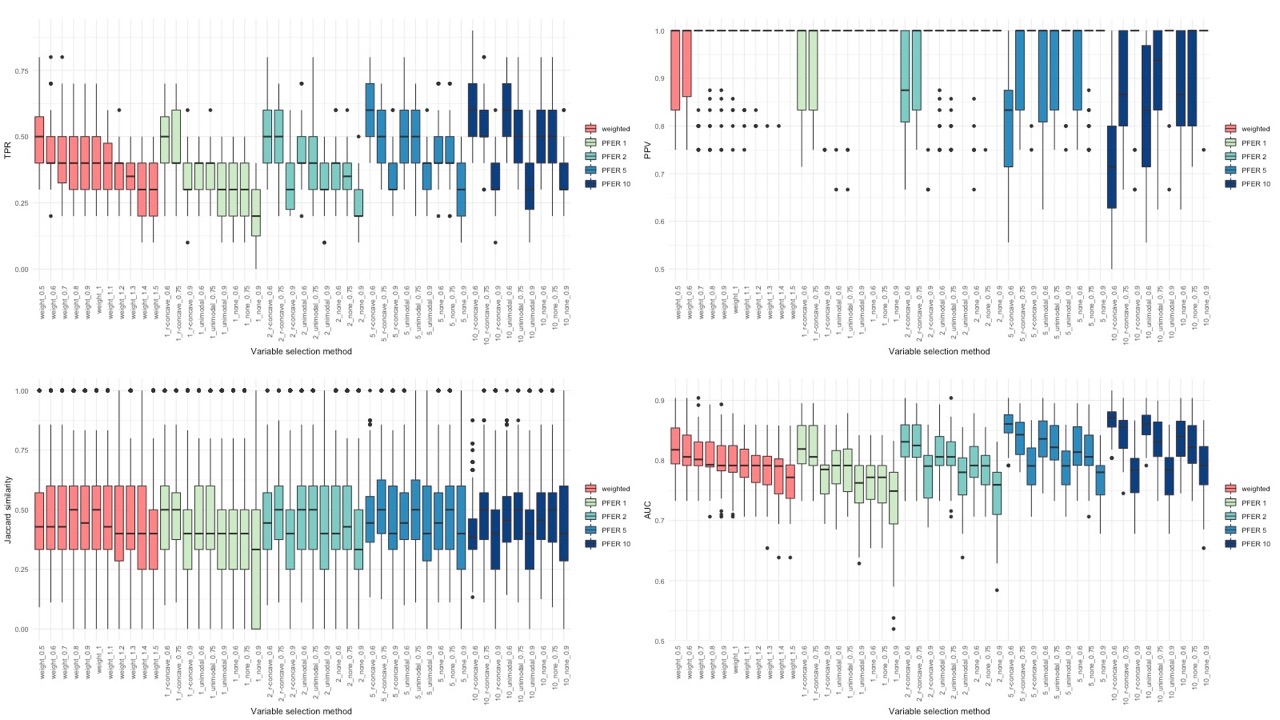


Box plots of scenario 44. $n=200, p=500, p_{signal}=10, \beta_{j}$’s of the signal variables $\sim U\left( 0.5,1.5 \right)$, event prevalence$=0.5$ and the covariance structure of $X=$independent.


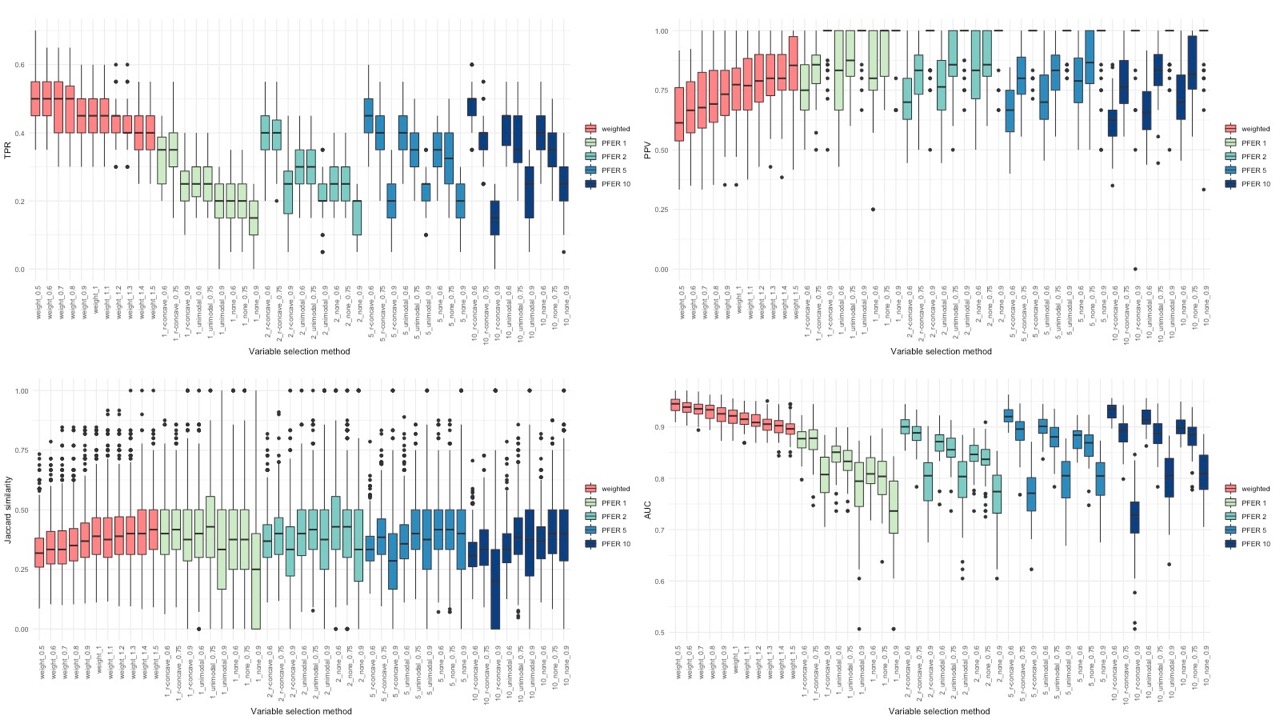


Box plots of scenario 45. $n=500, p=1000, p_{signal}=20, \beta_{j}$’s of the signal variables $\sim U\left( -3,3 \right)$, event prevalence$=0.3$ and the covariance structure of $X=$Toeplitz.


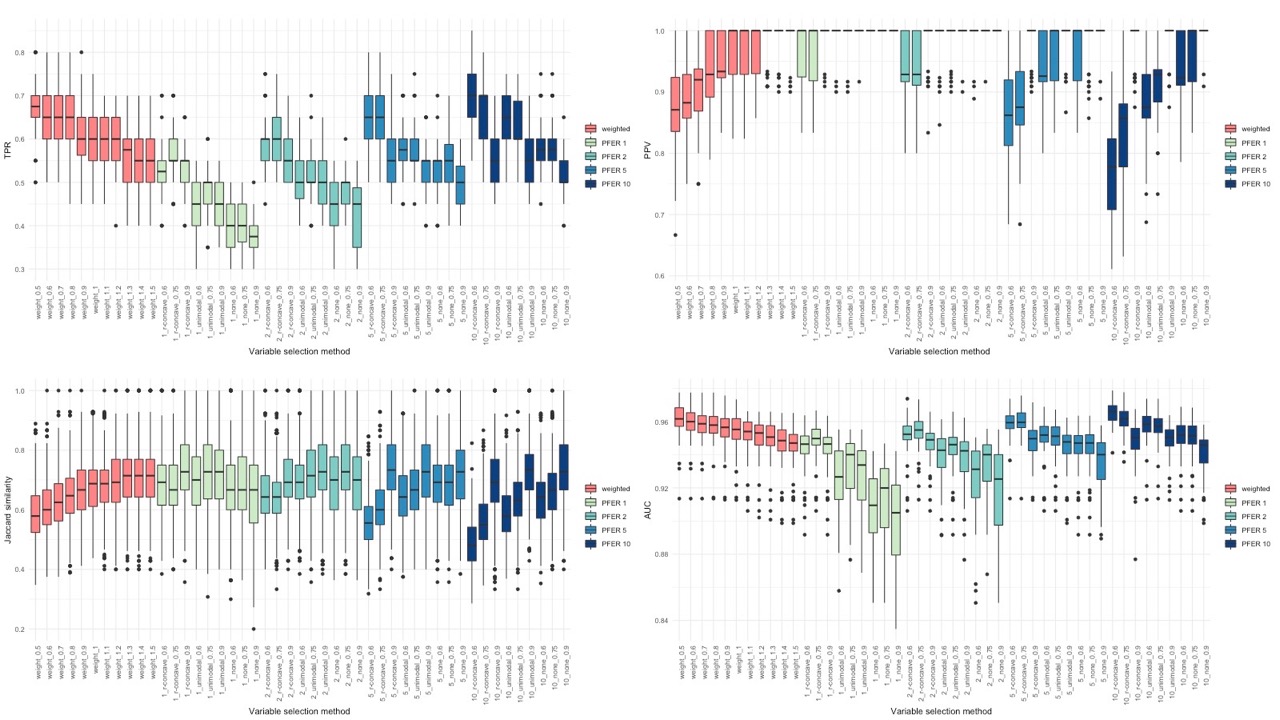


Box plots of scenario 46. $n=500, p=1000, p_{signal}=20, \beta_{j}$’s of the signal variables $\sim U\left( -3,3 \right)$, event prevalence$=0.3$ and the covariance structure of $X=$independent.


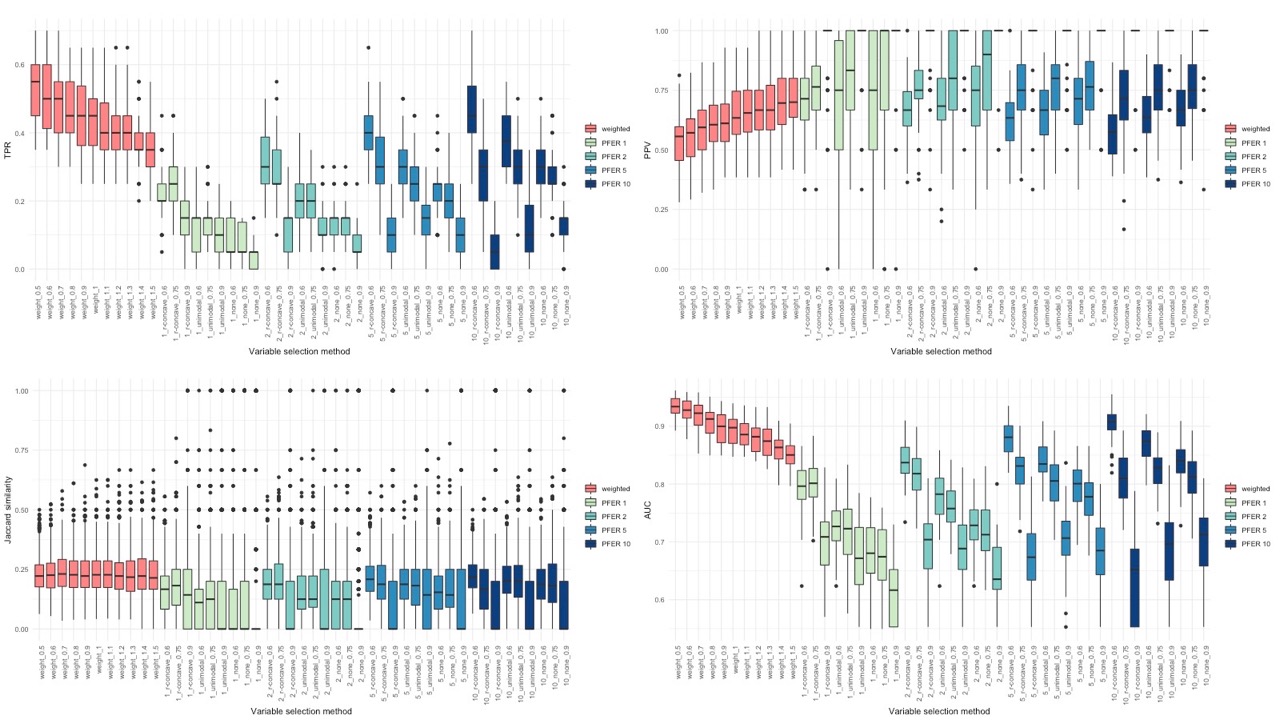


Box plots of scenario 47. $n=500, p=1000, p_{signal}=20, \beta_{j}$’s of the signal variables $\sim U\left( 0.5,1.5 \right)$, event prevalence$=0.3$ and the covariance structure of $X=$Toeplitz.


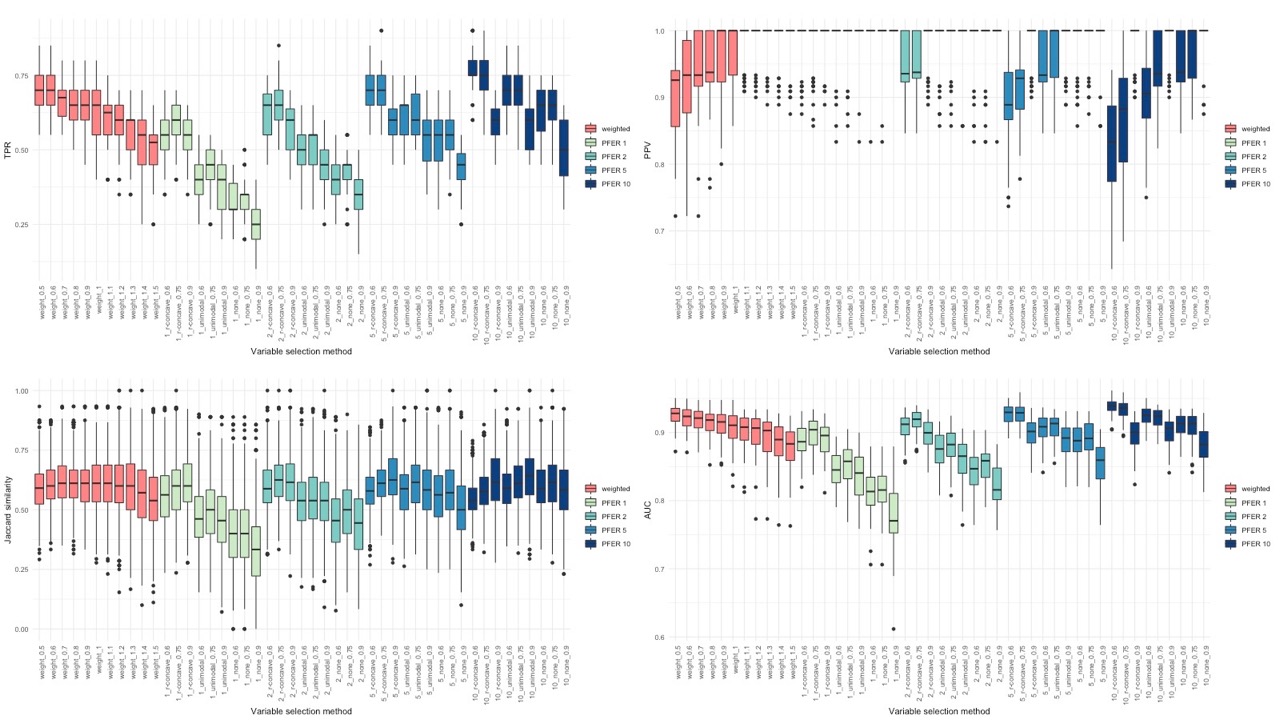


Box plots of scenario 48. $n=500, p=1000, p_{signal}=20, \beta_{j}$’s of the signal variables $\sim U\left( 0.5,1.5 \right)$, event prevalence$=0.3$ and the covariance structure of $X=$independent.


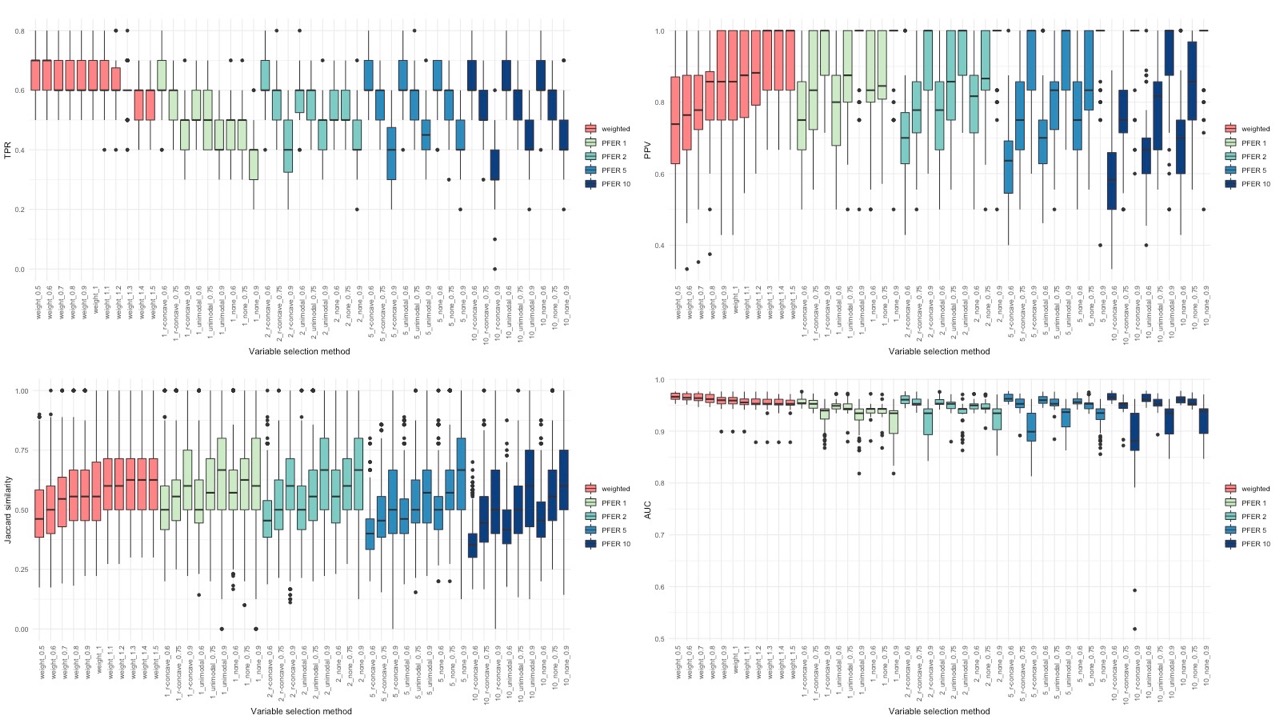


Box plots of scenario 49. $n=500, p=1000, p_{signal}=10, \beta_{j}$’s of the signal variables $\sim U\left( -3,3 \right)$, event prevalence$=0.3$ and the covariance structure of $X=$Toeplitz.


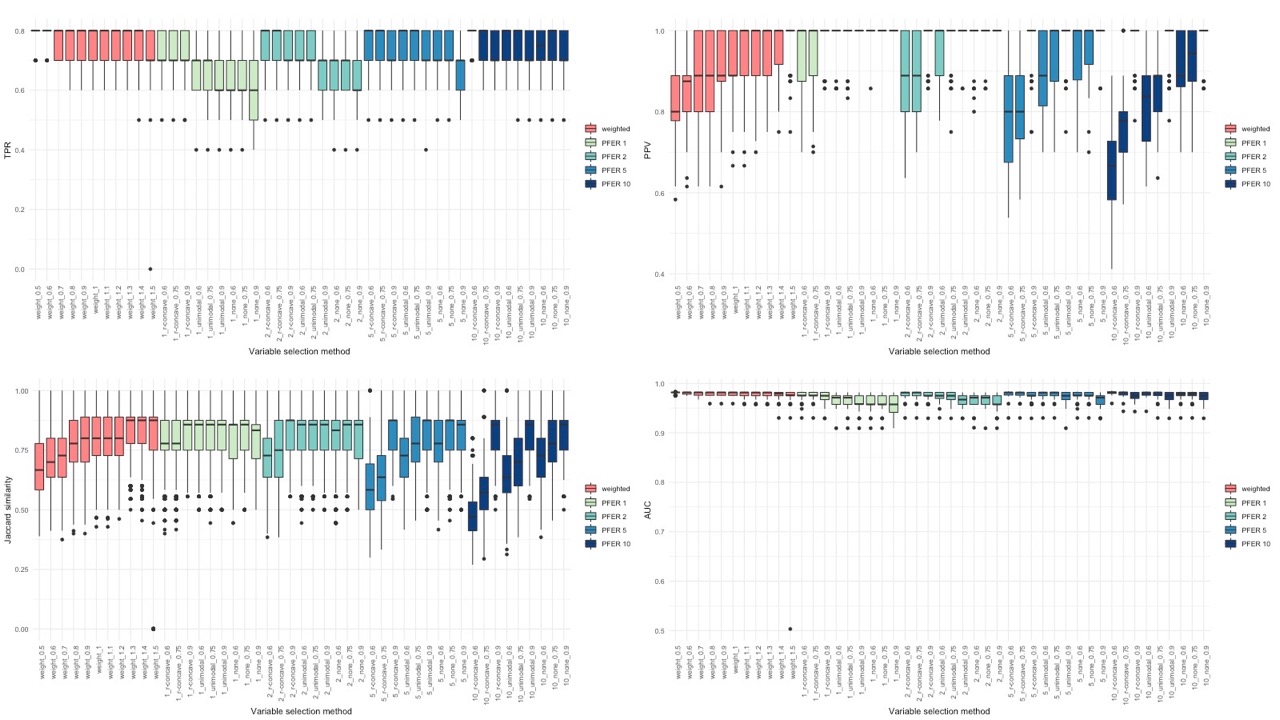


Box plots of scenario 50. $n=500, p=1000, p_{signal}=10, \beta_{j}$’s of the signal variables $\sim U\left( -3,3 \right)$, event prevalence$=0.3$ and the covariance structure of $X=$independent.


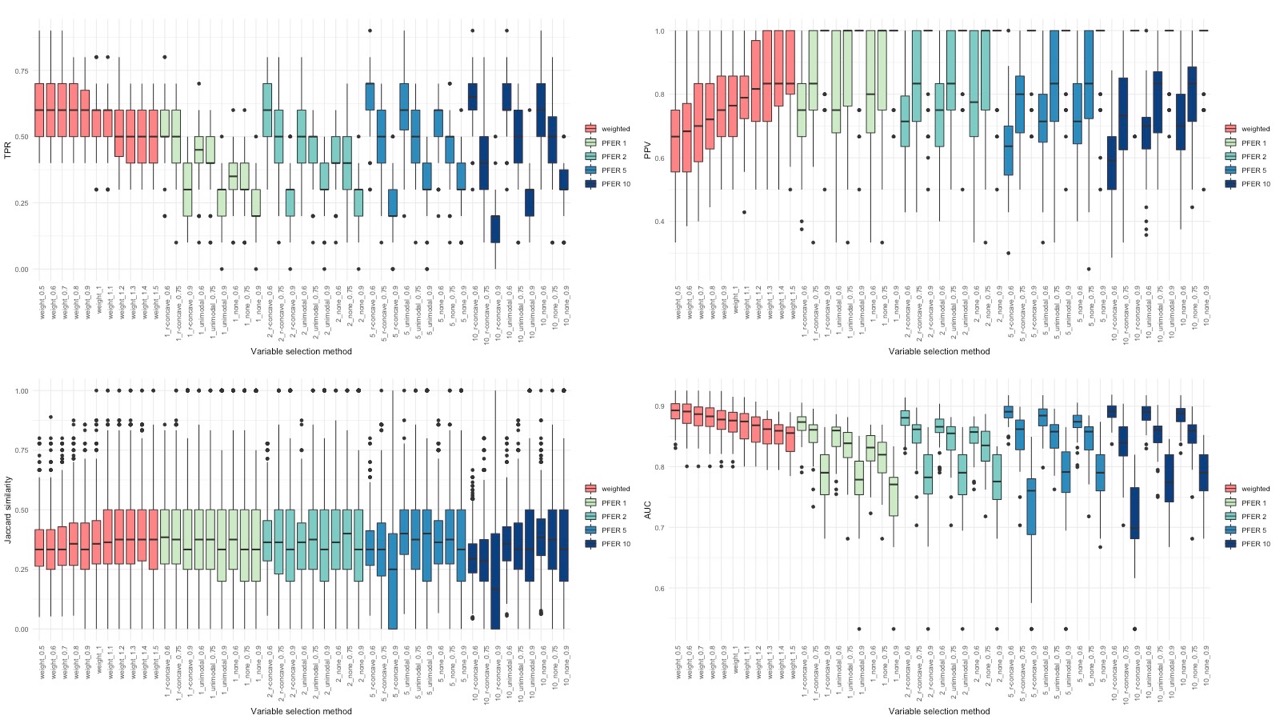


Box plots of scenario 51. $n=500, p=1000, p_{signal}=10, \beta_{j}$’s of the signal variables $\sim U\left( 0.5,1.5 \right)$, event prevalence$=0.3$ and the covariance structure of $X=$Toeplitz.


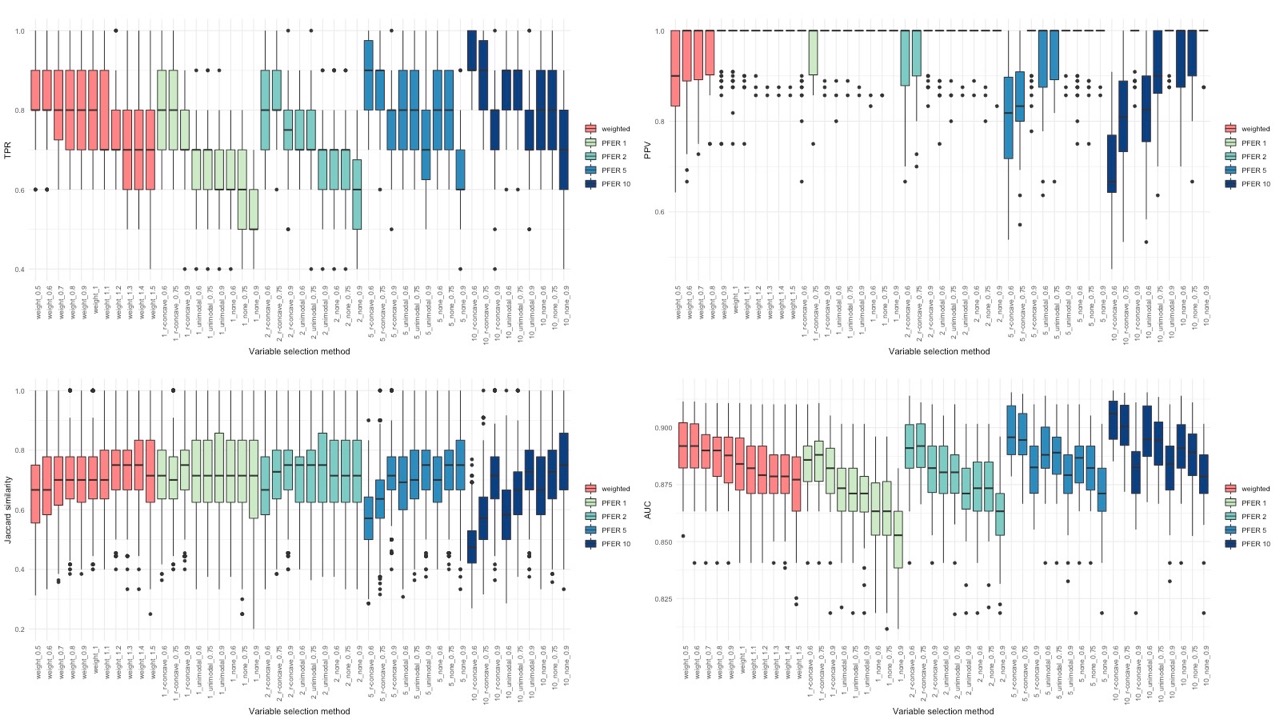


Box plots of scenario 52. $n=500, p=1000, p_{signal}=10, \beta_{j}$’s of the signal variables $\sim U\left( 0.5,1.5 \right)$, event prevalence$=0.3$ and the covariance structure of $X=$independent.


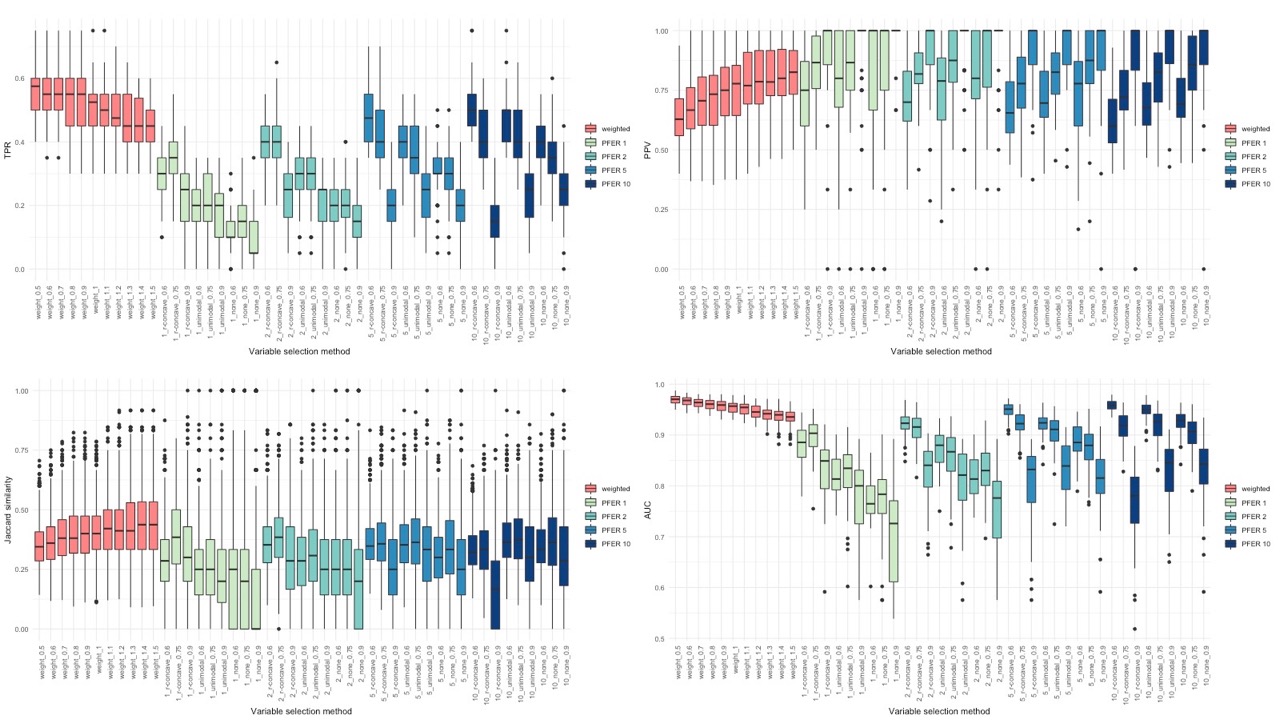


Box plots of scenario 53. $n=500, p=700, p_{signal}=20, \beta_{j}$’s of the signal variables $\sim U\left( -3,3 \right)$, event prevalence$=0.3$ and the covariance structure of $X=$Toeplitz.


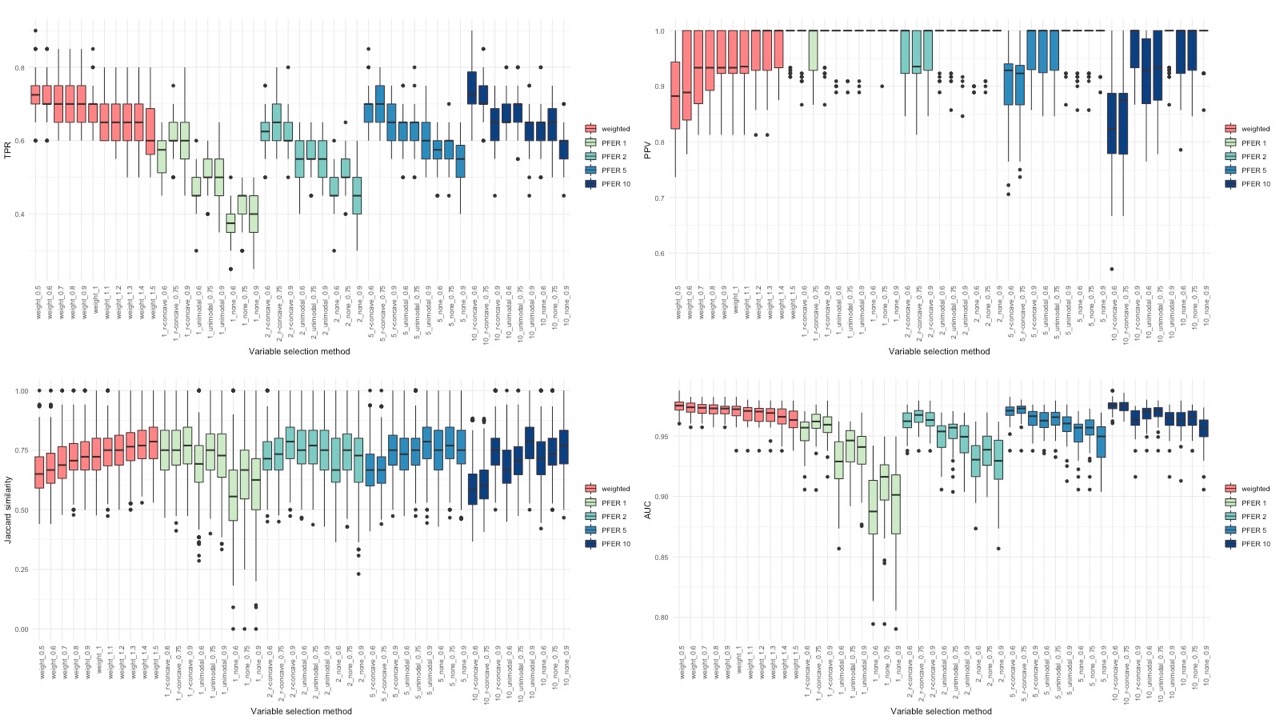


Box plots of scenario 54. $n=500, p=700, p_{signal}=20, \beta_{j}$’s of the signal variables $\sim U\left( -3,3 \right)$, event prevalence$=0.3$ and the covariance structure of $X=$independent.


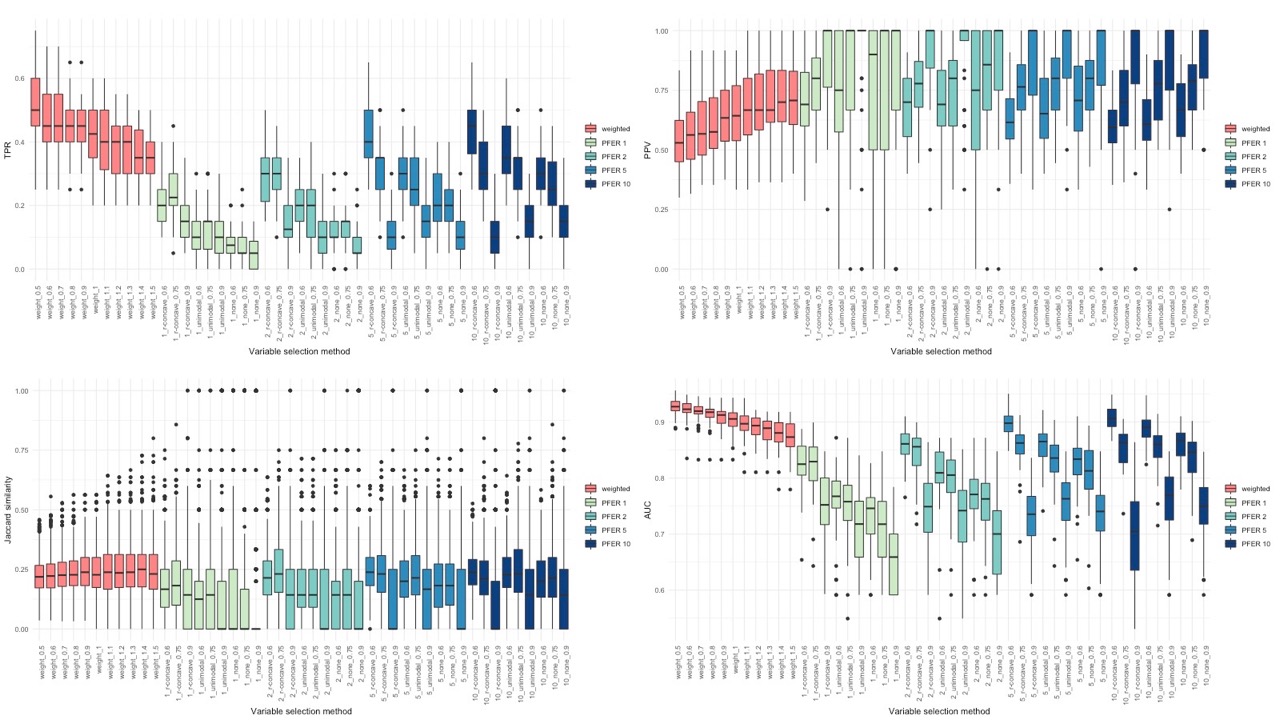


Box plots of scenario 55. $n=500, p=700, p_{signal}=20, \beta_{j}$’s of the signal variables $\sim U\left( 0.5,1.5 \right)$, event prevalence$=0.3$ and the covariance structure of $X=$Toeplitz.


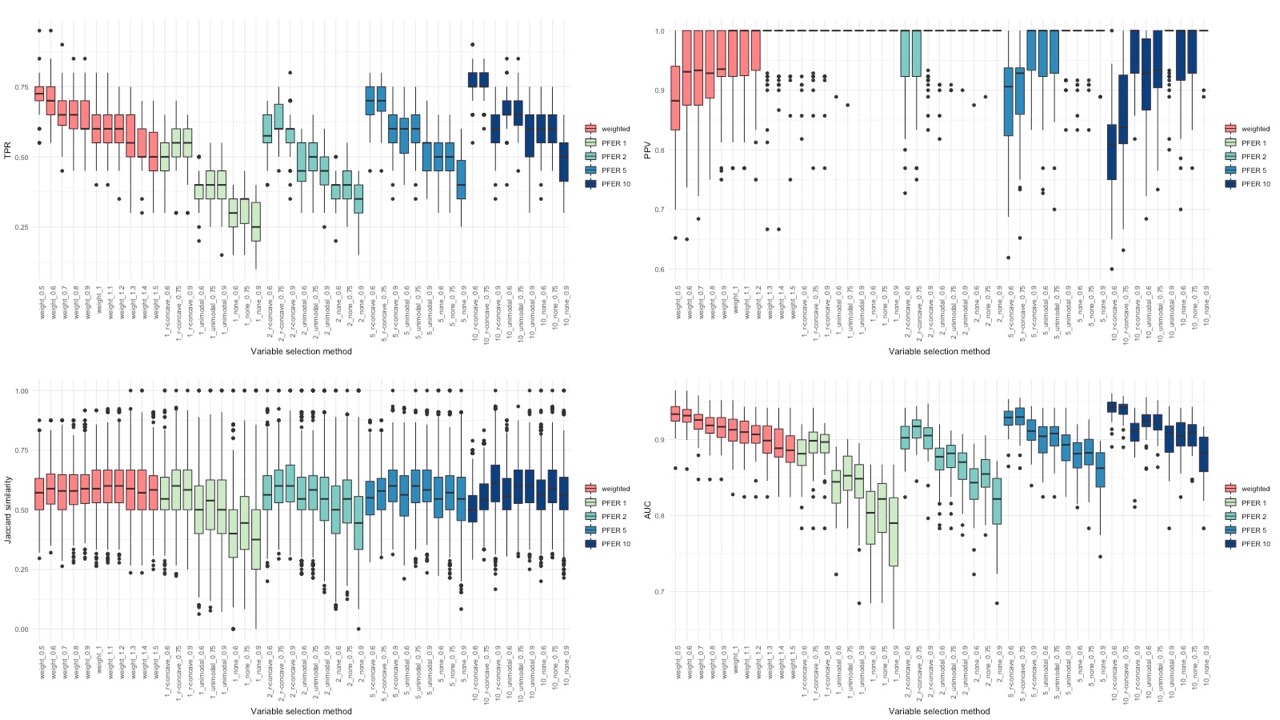


Box plots of scenario 56. $n=500, p=700, p_{signal}=20, \beta_{j}$’s of the signal variables $\sim U\left( 0.5,1.5 \right)$, event prevalence$=0.3$ and the covariance structure of $X=$independent.


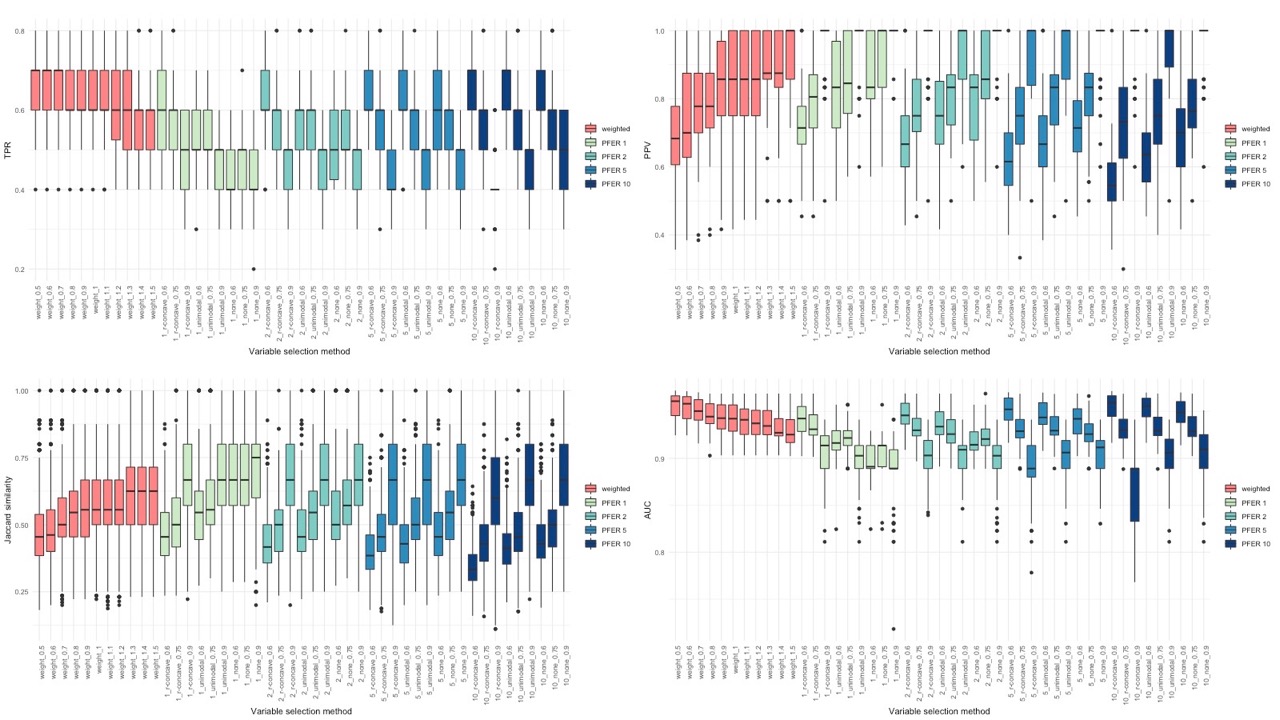


Box plots of scenario 57. $n=500, p=700, p_{signal}=10, \beta_{j}$’s of the signal variables $\sim U\left( -3,3 \right)$, event prevalence$=0.3$ and the covariance structure of $X=$Toeplitz.


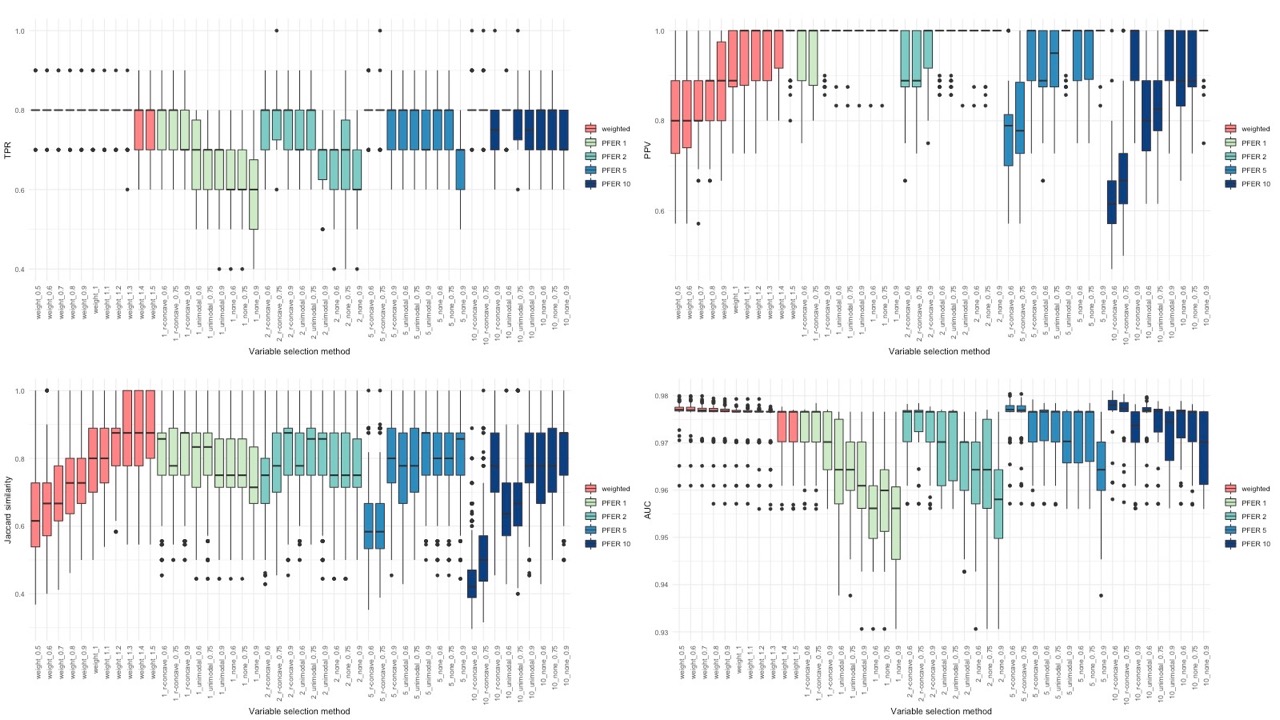


Box plots of scenario 58. $n=500, p=700, p_{signal}=10, \beta_{j}$’s of the signal variables $\sim U\left( -3,3 \right)$, event prevalence$=0.3$ and the covariance structure of $X=$independent.


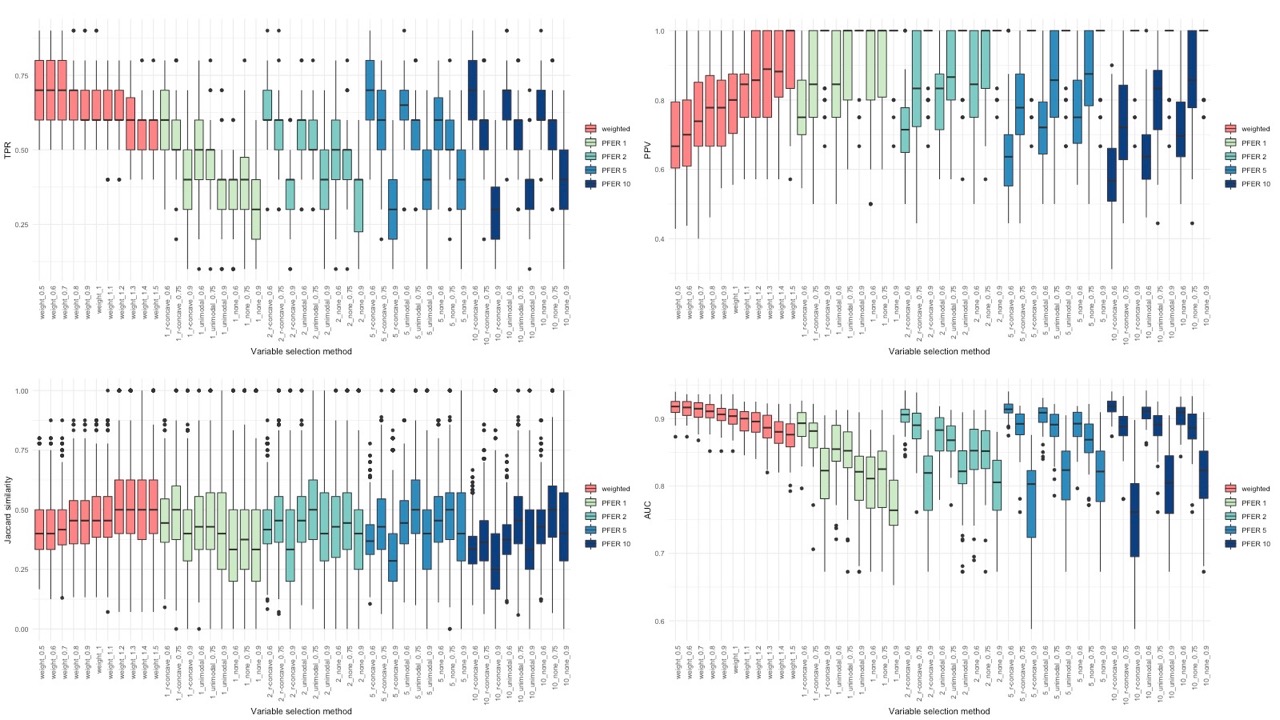


Box plots of scenario 59. $n=500, p=700, p_{signal}=10, \beta_{j}$’s of the signal variables $\sim U\left( 0.5,1.5 \right)$, event prevalence$=0.3$ and the covariance structure of $X=$Toeplitz.


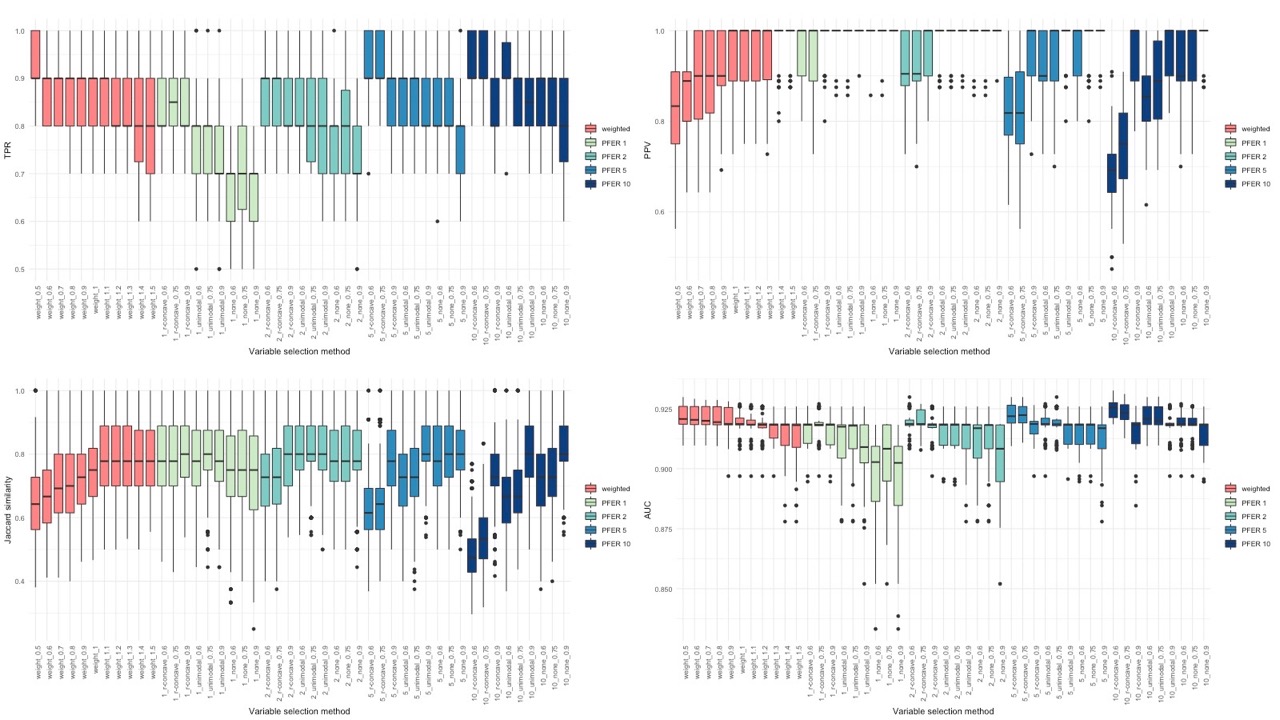


Box plots of scenario 60. $n=500, p=700, p_{signal}=10, \beta_{j}$’s of the signal variables $\sim U\left( 0.5,1.5 \right)$, event prevalence$=0.3$ and the covariance structure of $X=$independent.


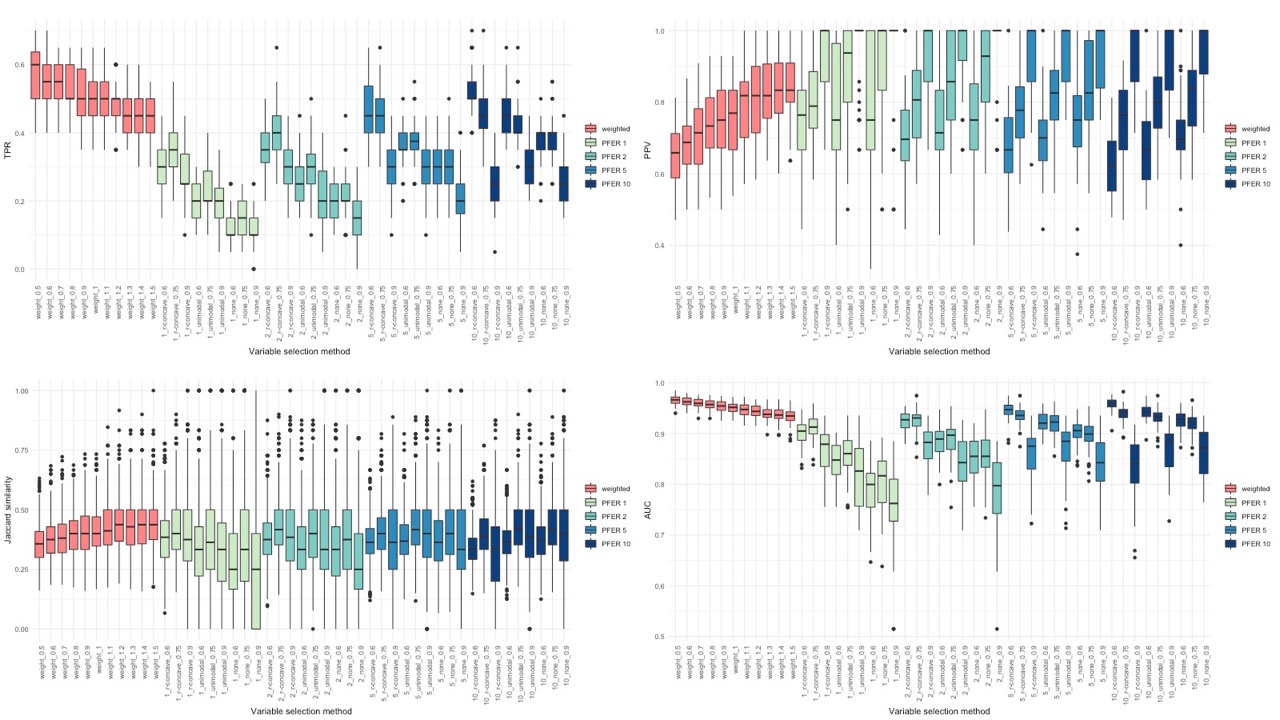


Box plots of scenario 61. $n=500, p=500, p_{signal}=20, \beta_{j}$’s of the signal variables $\sim U\left( -3,3 \right)$, event prevalence$=0.3$ and the covariance structure of $X=$Toeplitz.


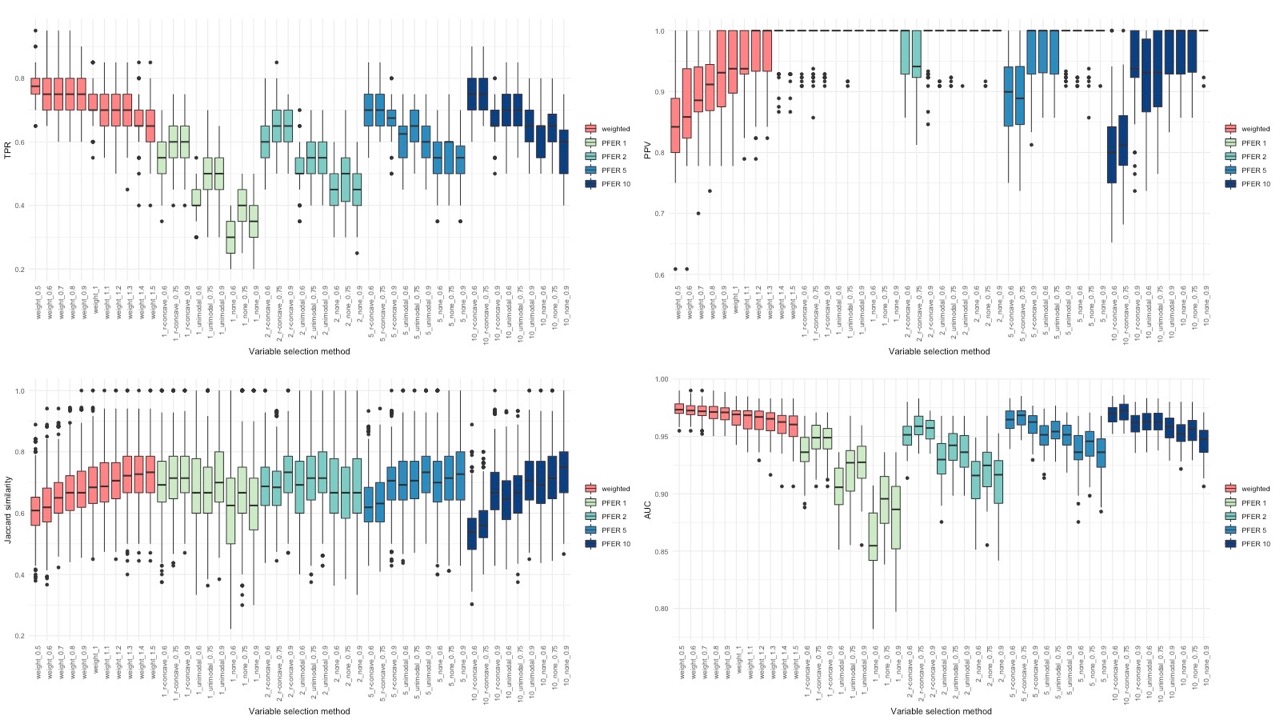


Box plots of scenario 62. $n=500, p=500, p_{signal}=20, \beta_{j}$’s of the signal variables $\sim U\left( -3,3 \right)$, event prevalence$=0.3$ and the covariance structure of $X=$independent.


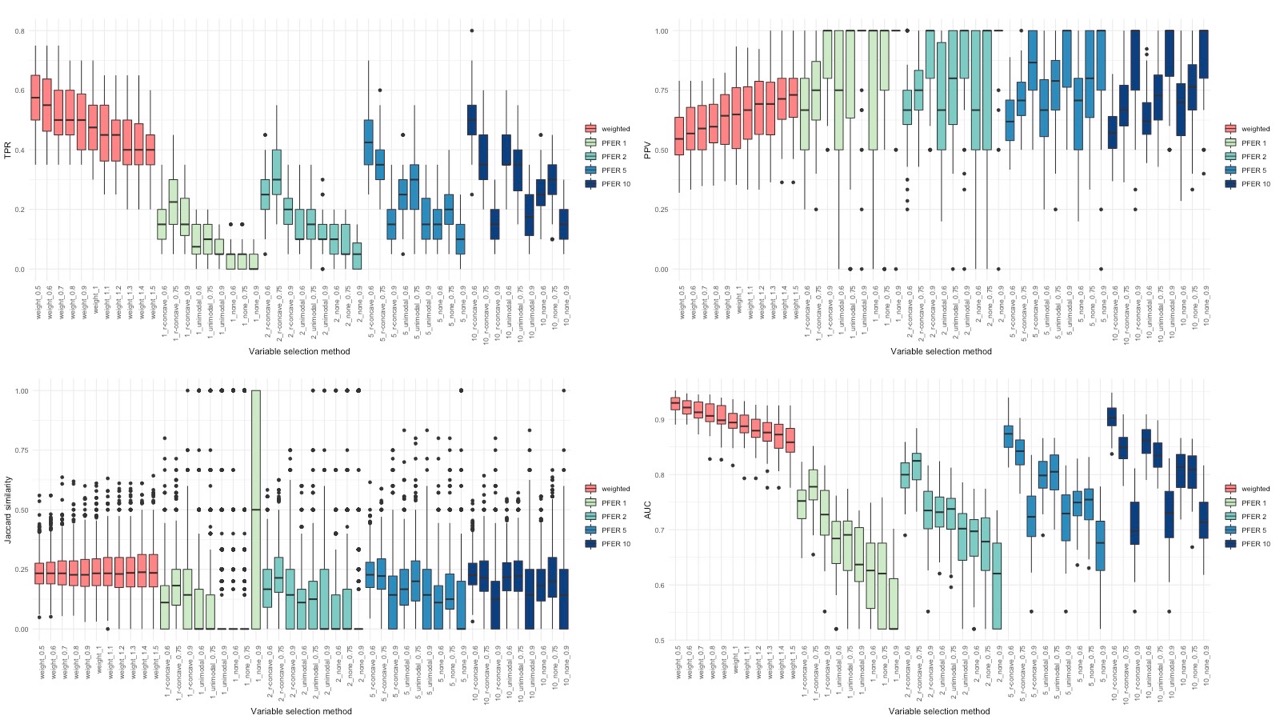


Box plots of scenario 63. $n=500, p=500, p_{signal}=20, \beta_{j}$’s of the signal variables $\sim U\left( 0.5,1.5 \right)$, event prevalence$=0.3$ and the covariance structure of $X=$Toeplitz.


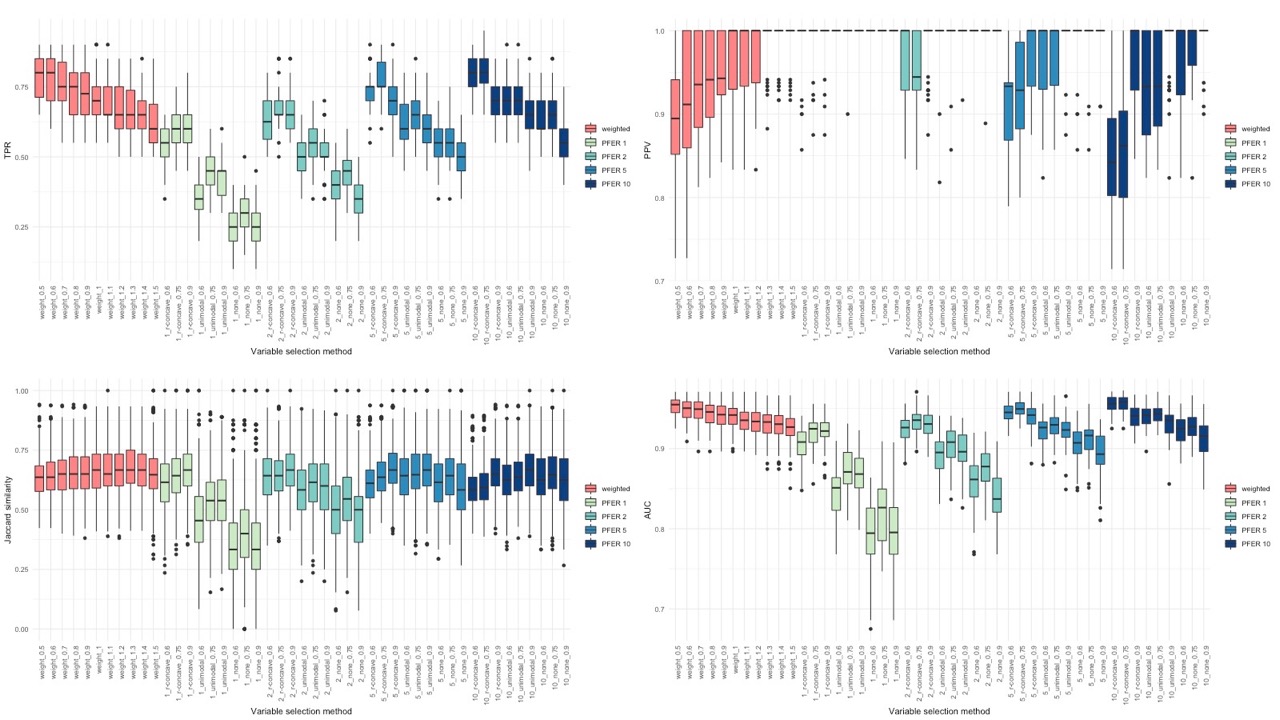


Box plots of scenario 64. $n=500, p=500, p_{signal}=20, \beta_{j}$’s of the signal variables $\sim U\left( 0.5,1.5 \right)$, event prevalence$=0.3$ and the covariance structure of $X=$independent.


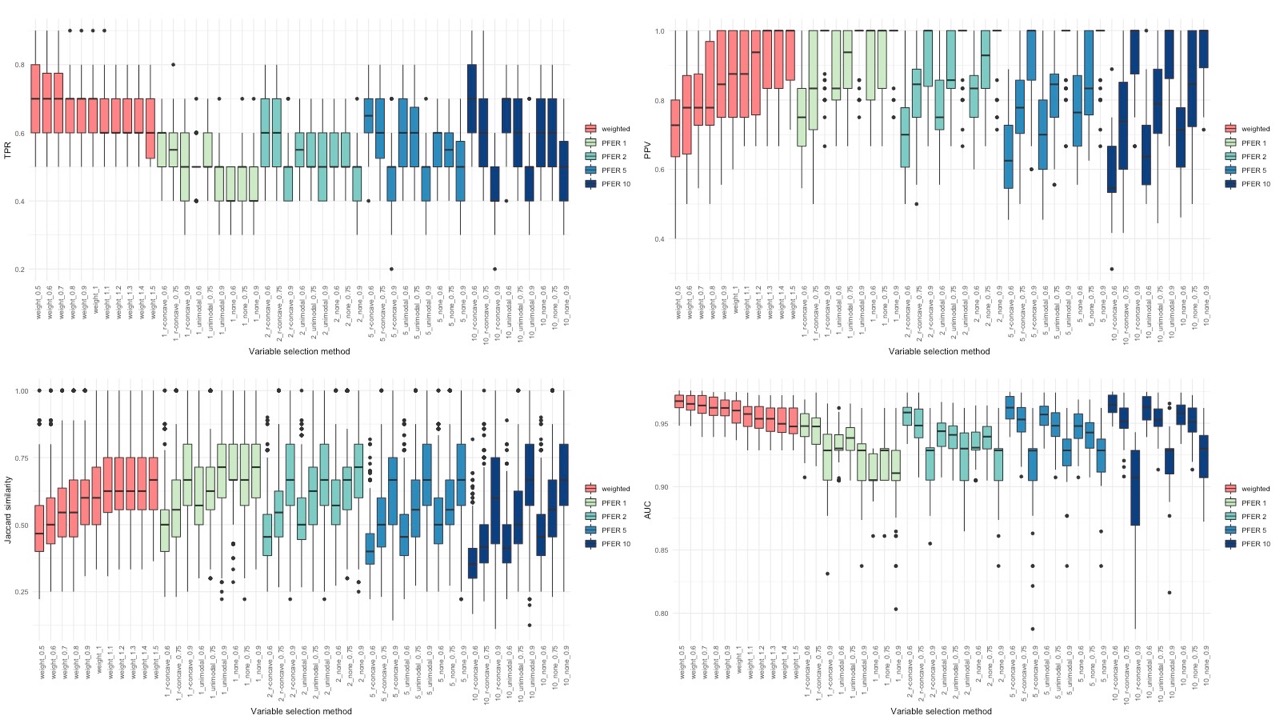


Box plots of scenario 65. $n=500, p=500, p_{signal}=10, \beta_{j}$’s of the signal variables $\sim U\left( -3,3 \right)$, event prevalence$=0.3$ and the covariance structure of $X=$Toeplitz.


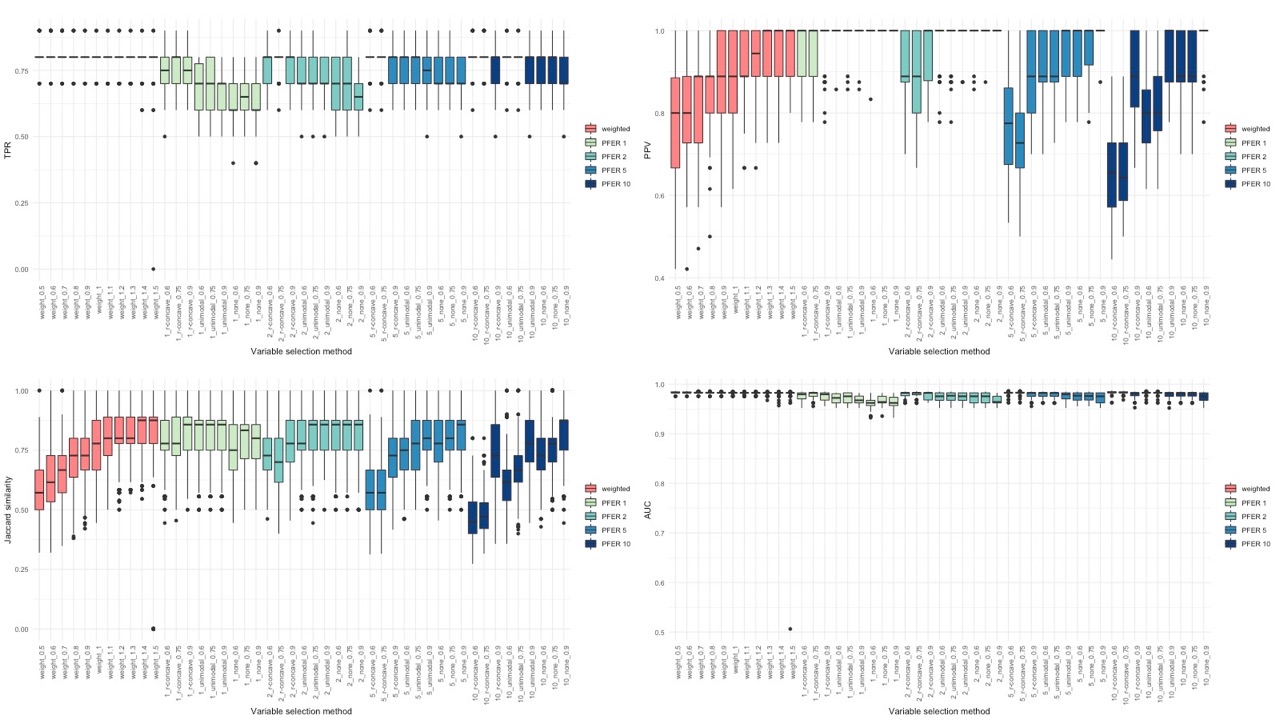


Box plots of scenario 66. $n=500, p=500, p_{signal}=10, \beta_{j}$’s of the signal variables $\sim U\left( -3,3 \right)$, event prevalence$=0.3$ and the covariance structure of $X=$independent.


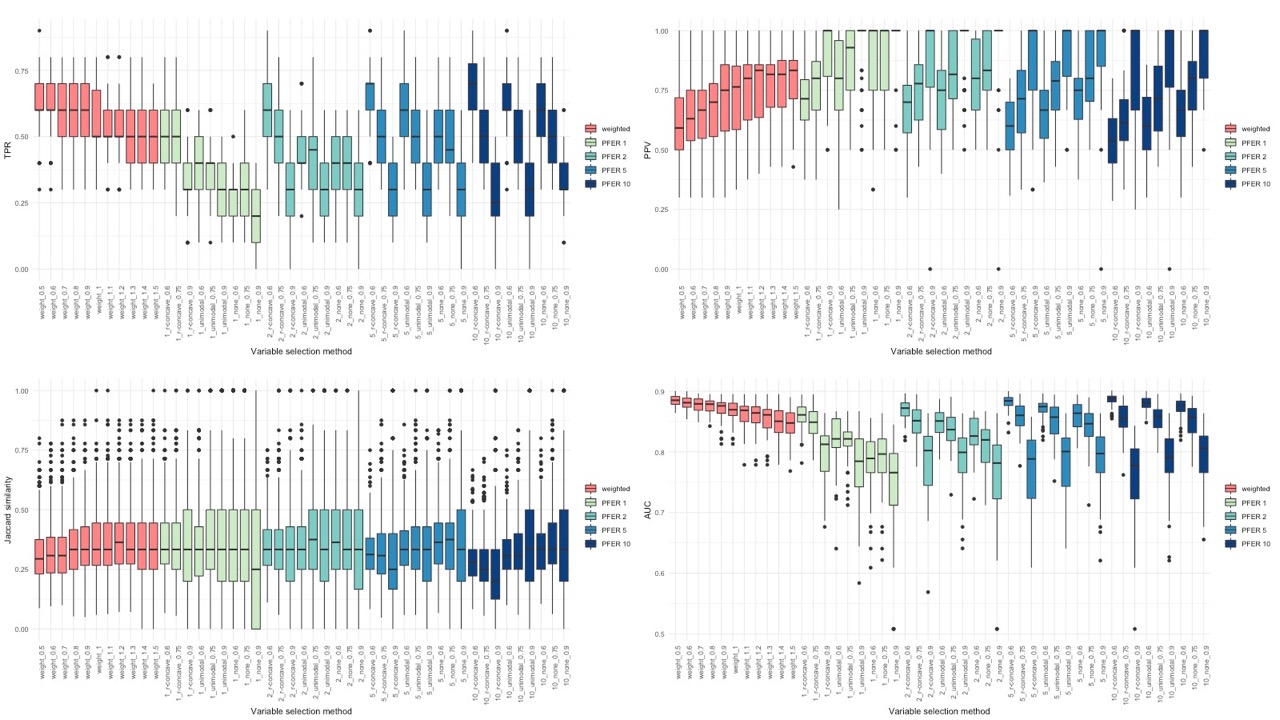


Box plots of scenario 67. $n=500, p=500, p_{signal}=10, \beta_{j}$’s of the signal variables $\sim U\left( 0.5,1.5 \right)$, event prevalence$=0.3$ and the covariance structure of $X=$Toeplitz.


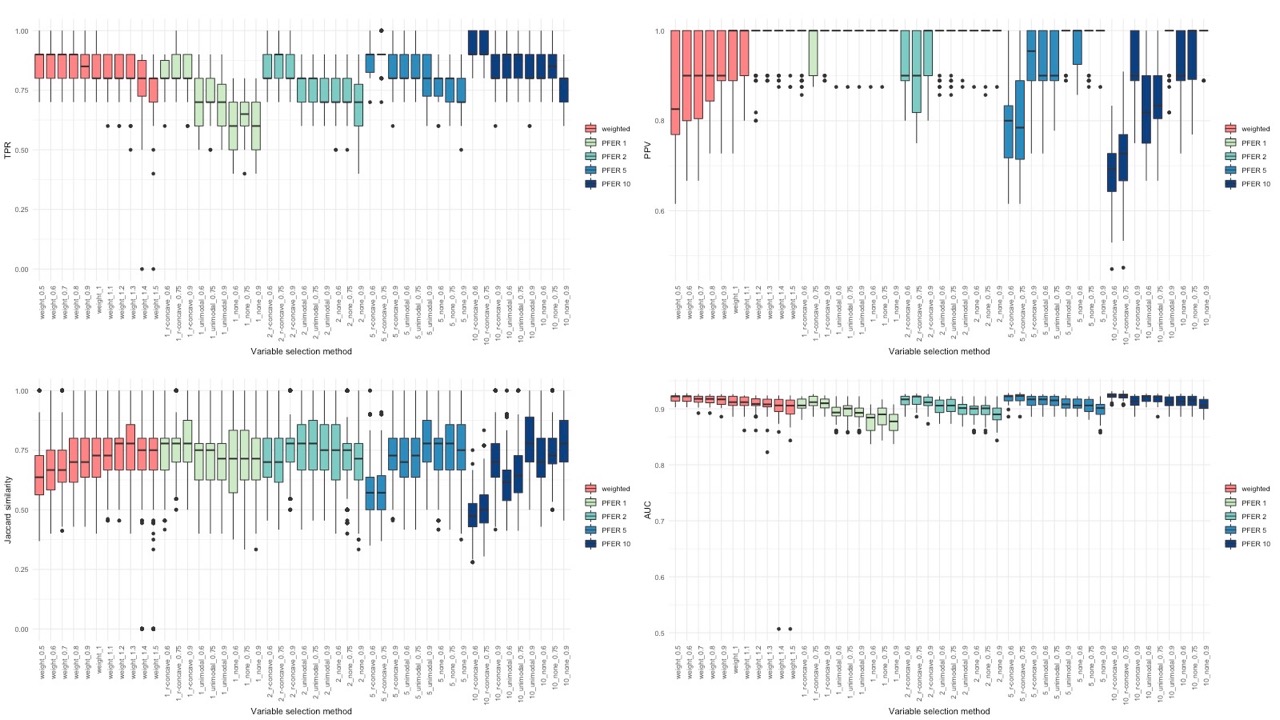


Box plots of scenario 68. $n=500, p=500, p_{signal}=10, \beta_{j}$’s of the signal variables $\sim U\left( 0.5,1.5 \right)$, event prevalence$=0.3$ and the covariance structure of $X=$independent.


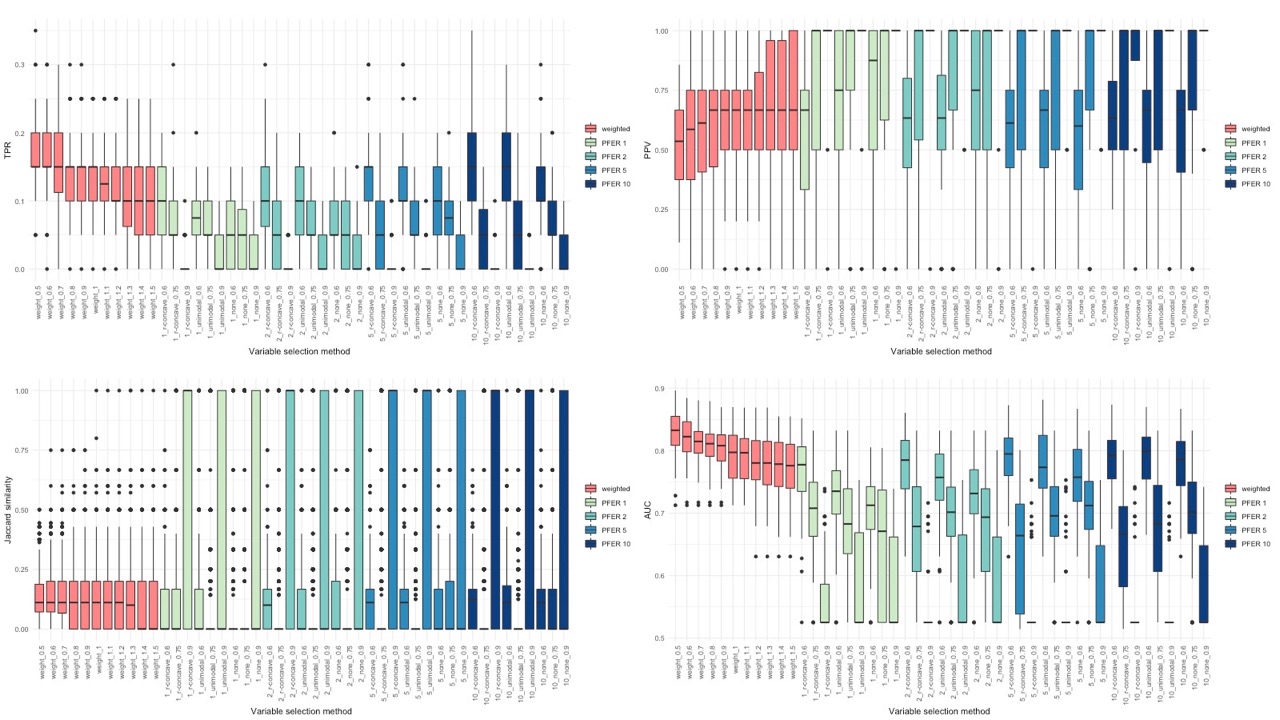


Box plots of scenario 69. $n=200, p=1000, p_{signal}=20, \beta_{j}$’s of the signal variables $\sim U\left( -3,3 \right)$, event prevalence$=0.3$ and the covariance structure of $X=$Toeplitz.


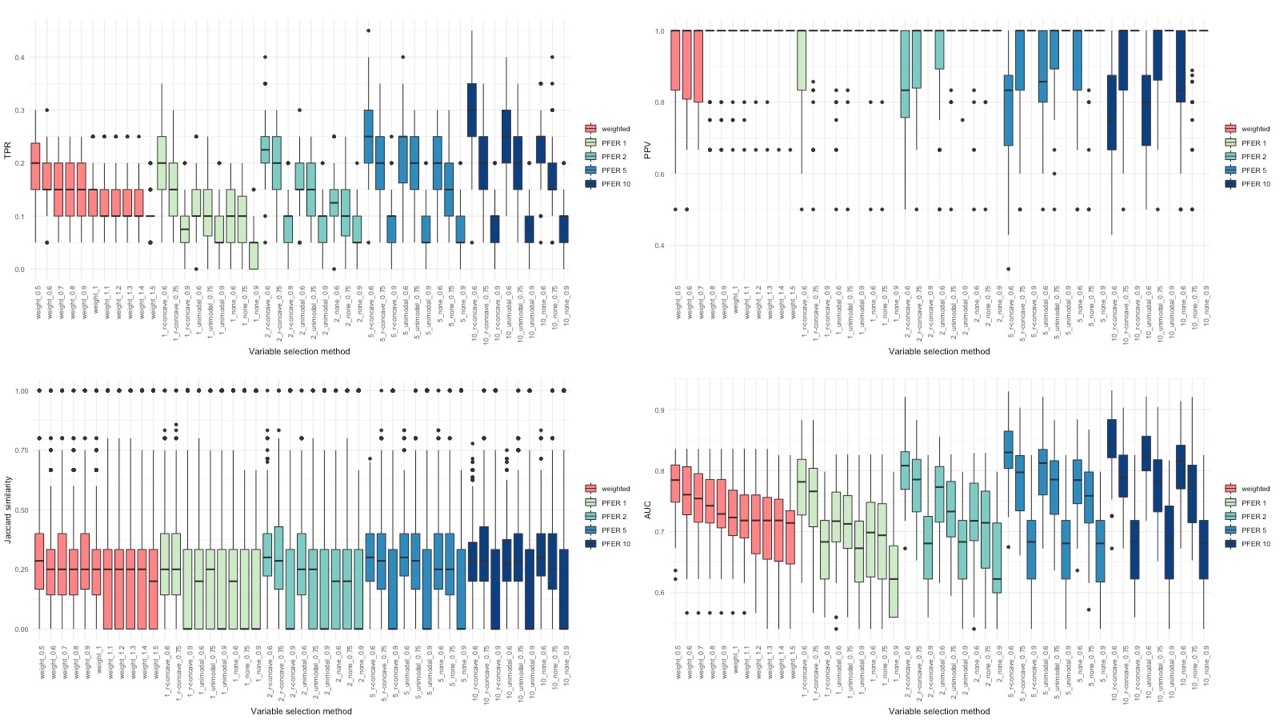


Box plots of scenario 70. $n=200, p=1000, p_{signal}=20, \beta_{j}$’s of the signal variables $\sim U\left( -3,3 \right)$, event prevalence$=0.3$ and the covariance structure of $X=$independent.


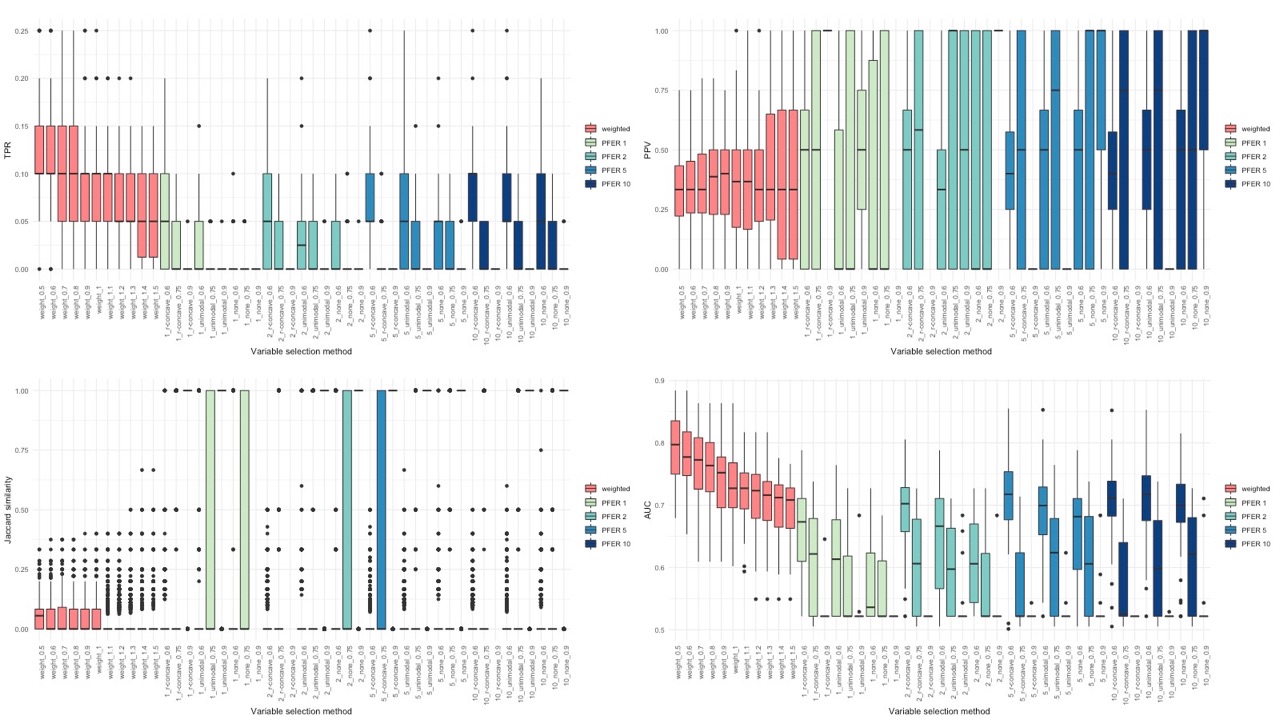


Box plots of scenario 71. $n=200, p=1000, p_{signal}=20, \beta_{j}$’s of the signal variables $\sim U\left( 0.5,1.5 \right)$, event prevalence$=0.3$ and the covariance structure of $X=$Toeplitz.


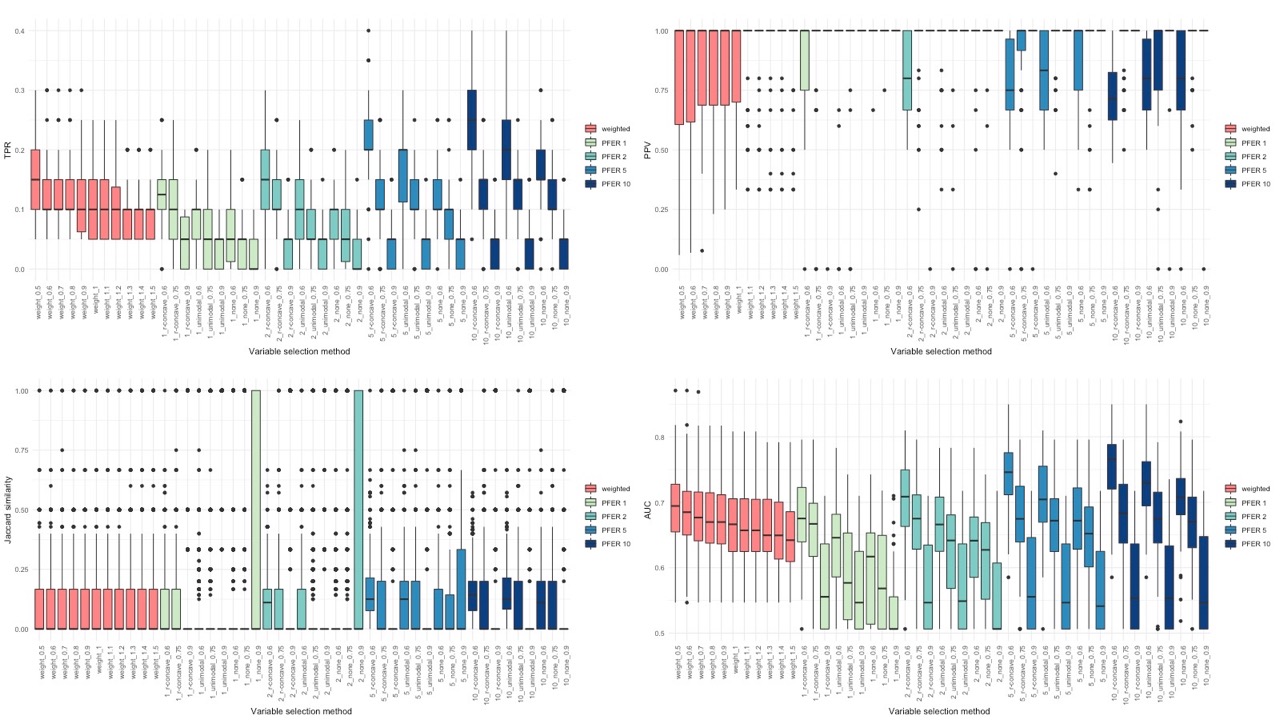


Box plots of scenario 72. $n=200, p=1000, p_{signal}=20, \beta_{j}$’s of the signal variables $\sim U\left( 0.5,1.5 \right)$, event prevalence$=0.3$ and the covariance structure of $X=$independent.


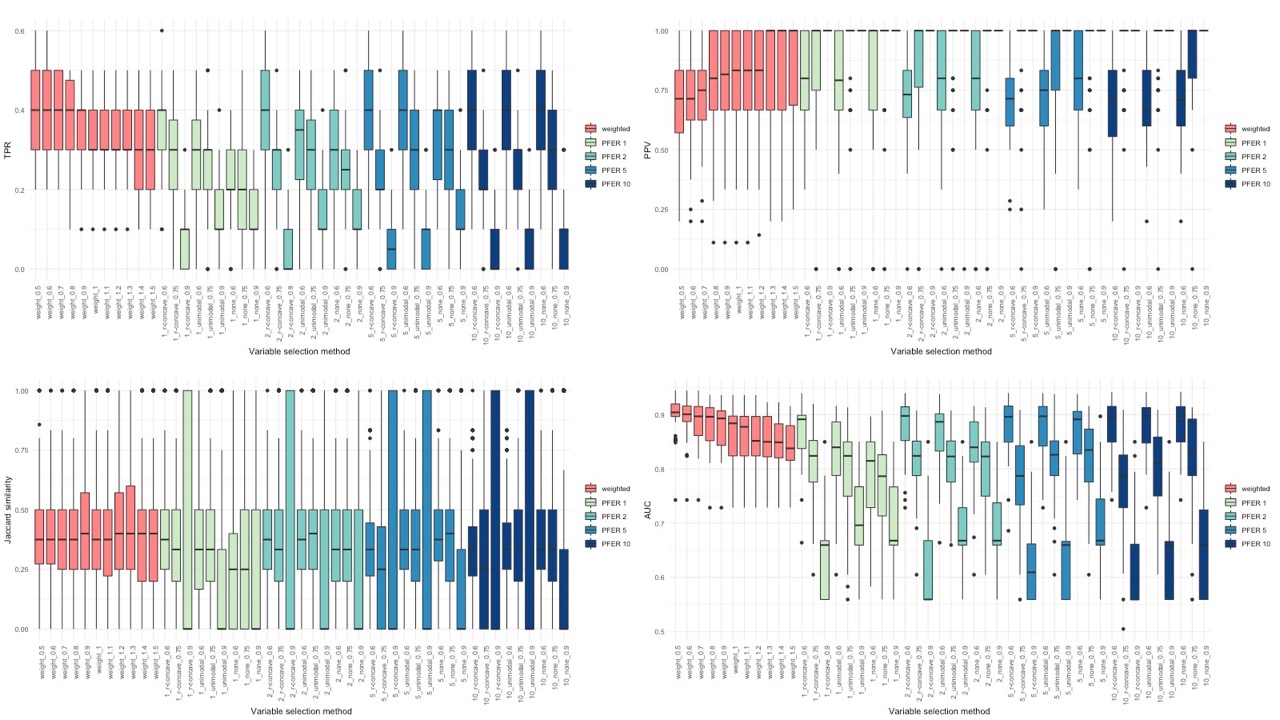


Box plots of scenario 73. $n=200, p=1000, p_{signal}=10, \beta_{j}$’s of the signal variables $\sim U\left( -3,3 \right)$, event prevalence$=0.3$ and the covariance structure of $X=$Toeplitz.


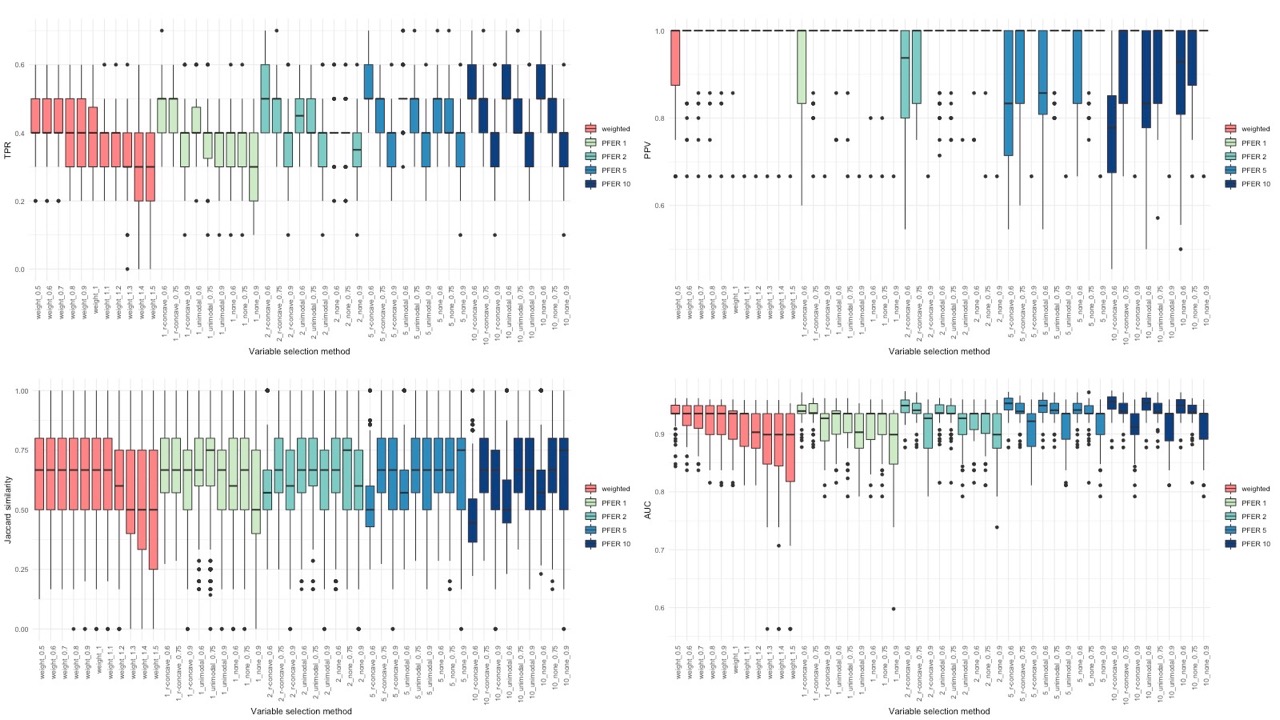


Box plots of scenario 74. $n=200, p=1000, p_{signal}=10, \beta_{j}$’s of the signal variables $\sim U\left( -3,3 \right)$, event prevalence$=0.3$ and the covariance structure of $X=$independent.


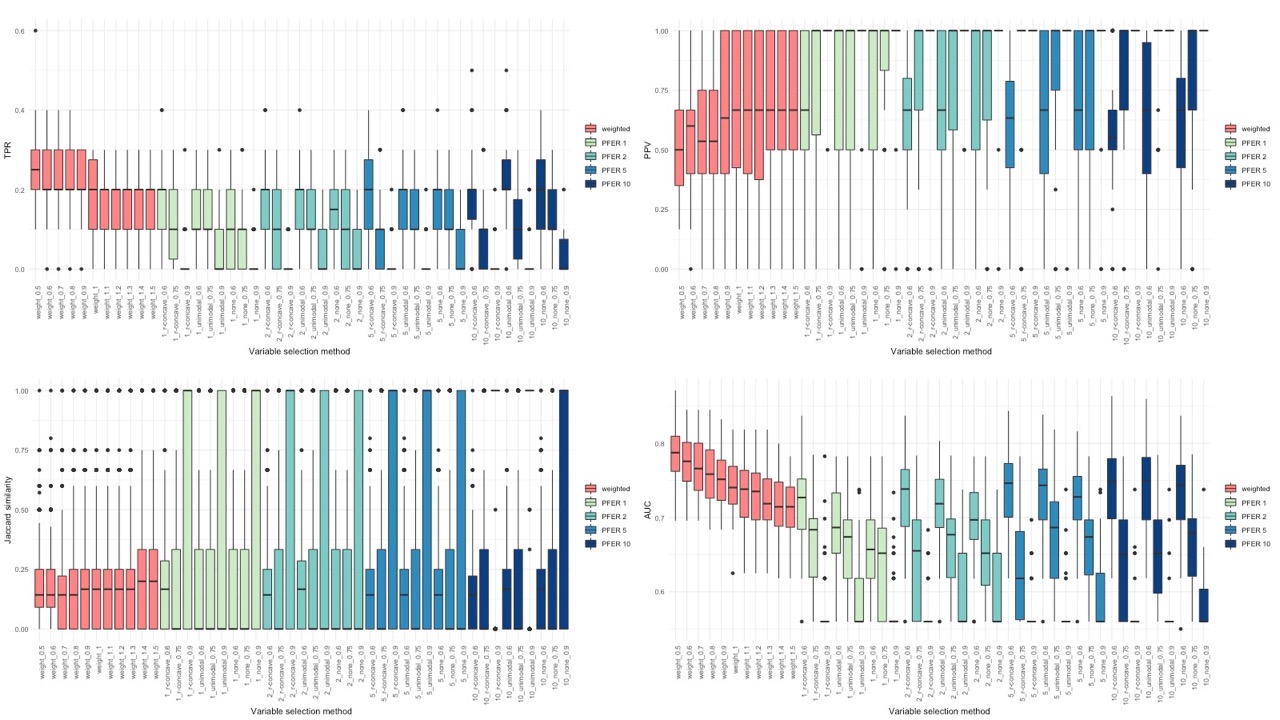


Box plots of scenario 75. $n=200, p=1000, p_{signal}=10, \beta_{j}$’s of the signal variables $\sim U\left( 0.5,1.5 \right)$, event prevalence$=0.3$ and the covariance structure of $X=$Toeplitz.


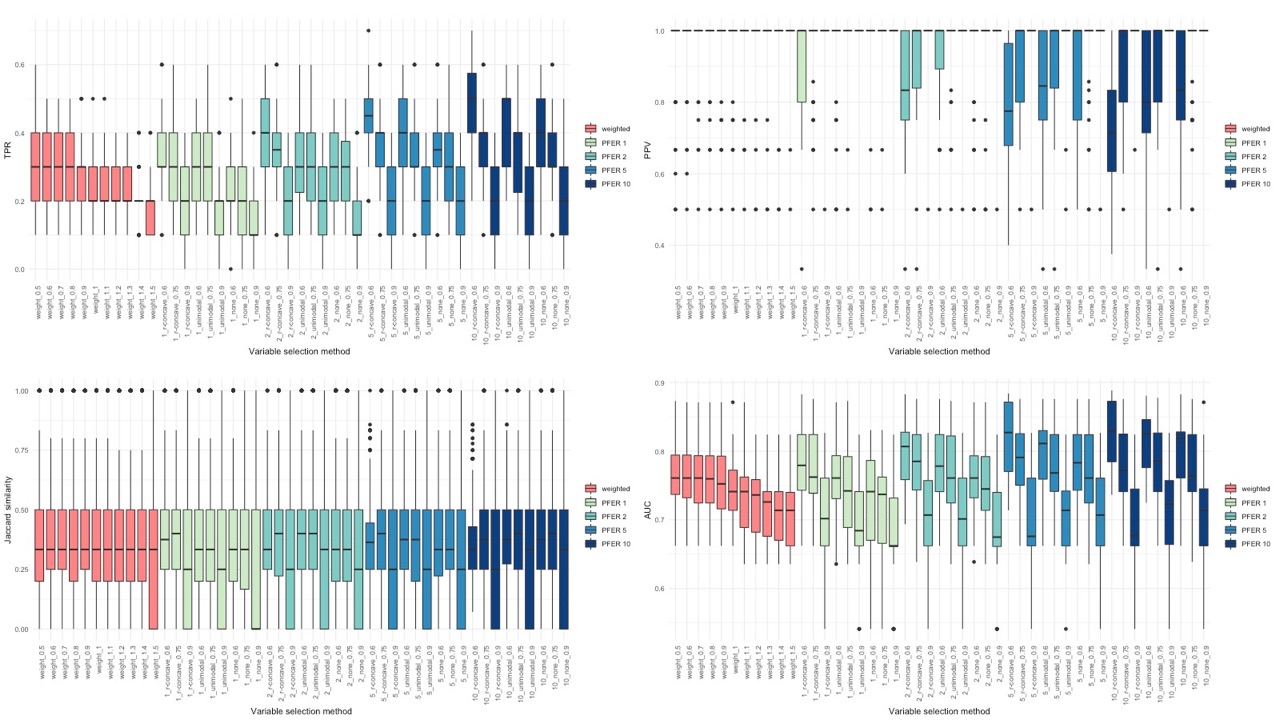


Box plots of scenario 76. $n=200, p=1000, p_{signal}=10, \beta_{j}$’s of the signal variables $\sim U\left( 0.5,1.5 \right)$, event prevalence$=0.3$ and the covariance structure of $X=$independent.


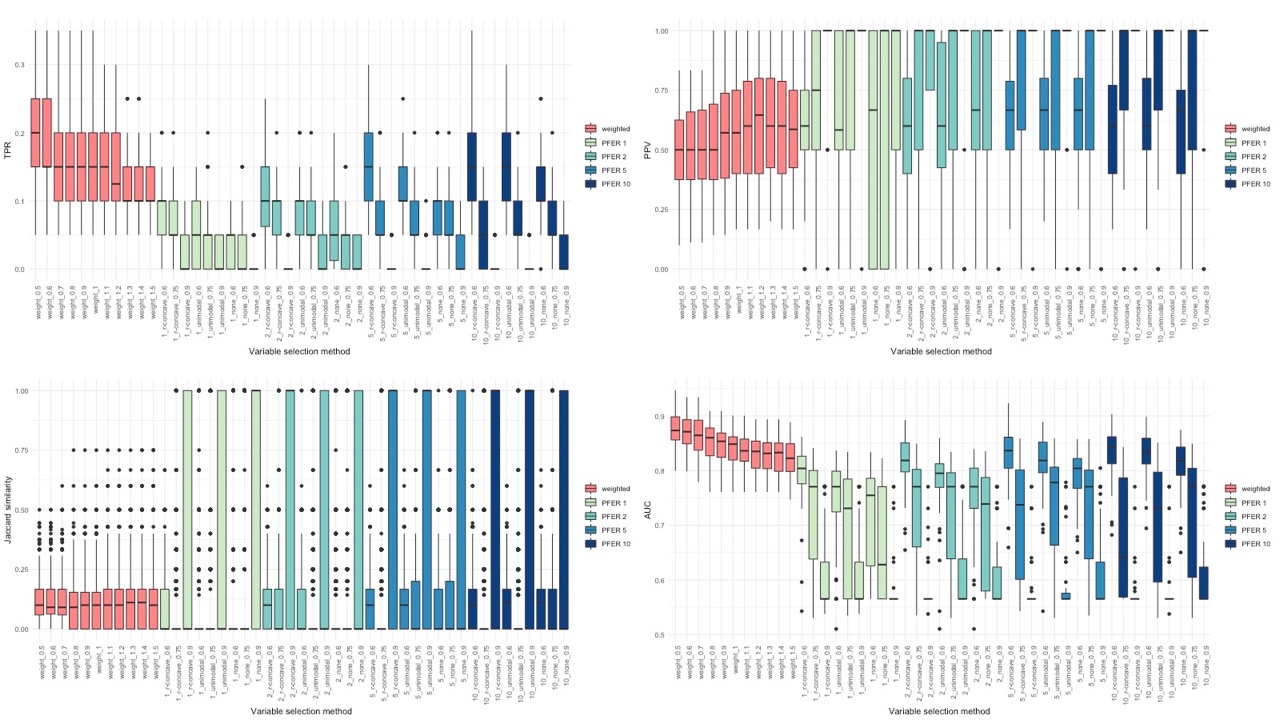


Box plots of scenario 77. $n=200, p=700, p_{signal}=20, \beta_{j}$’s of the signal variables $\sim U\left( -3,3 \right)$, event prevalence$=0.3$ and the covariance structure of $X=$Toeplitz.


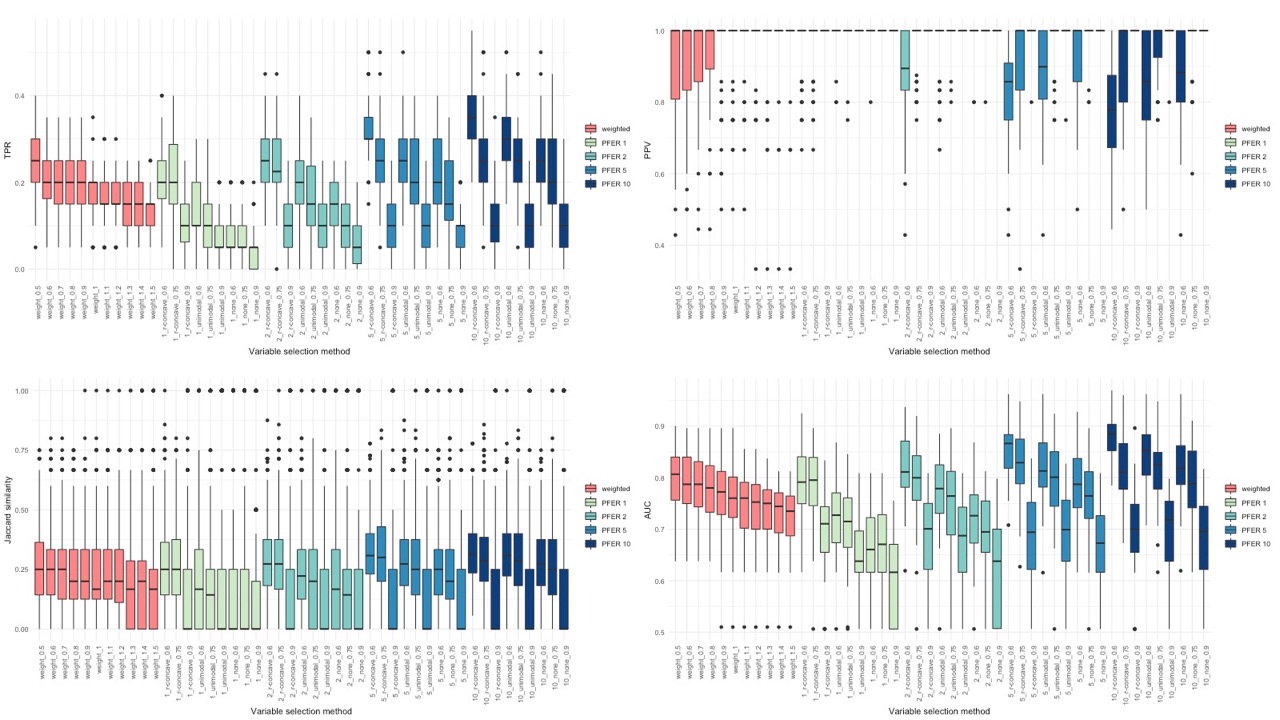


Box plots of scenario 78. $n=200, p=700, p_{signal}=20, \beta_{j}$’s of the signal variables $\sim U\left( -3,3 \right)$, event prevalence$=0.3$ and the covariance structure of $X=$independent.


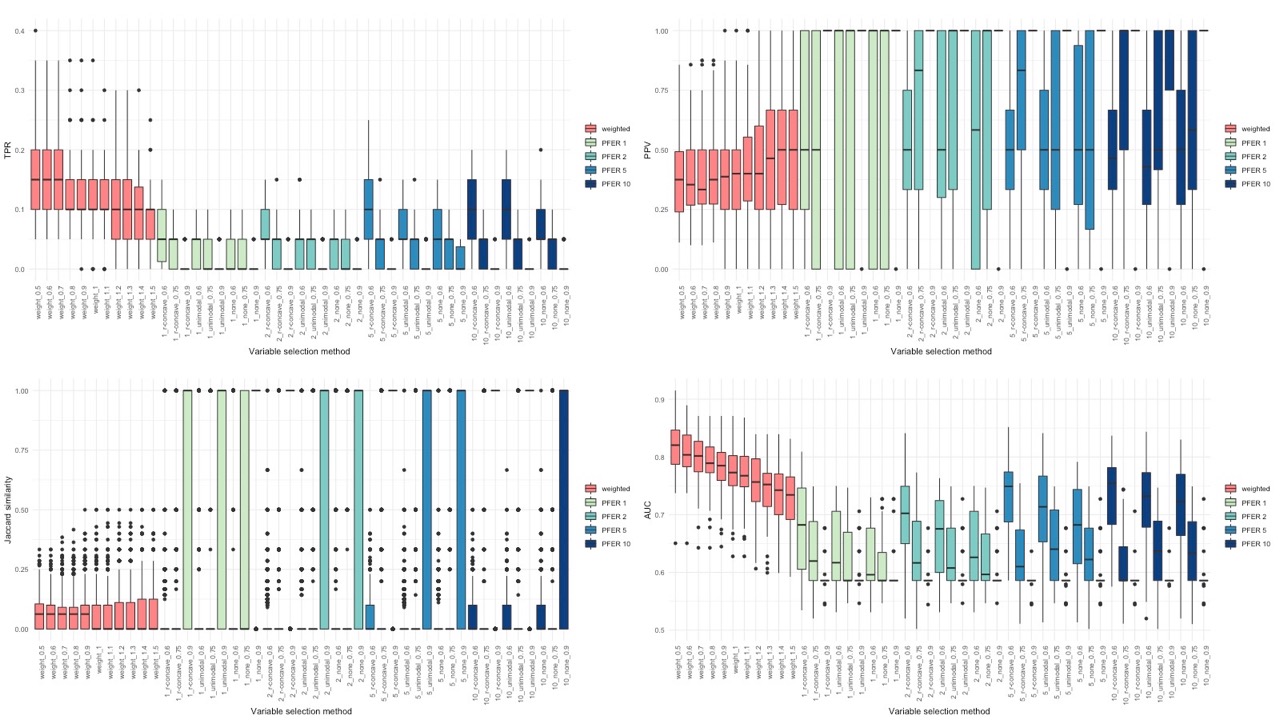


Box plots of scenario 79. $n=200, p=700, p_{signal}=20, \beta_{j}$’s of the signal variables $\sim U\left( 0.5,1.5 \right)$, event prevalence$=0.3$ and the covariance structure of $X=$Toeplitz.


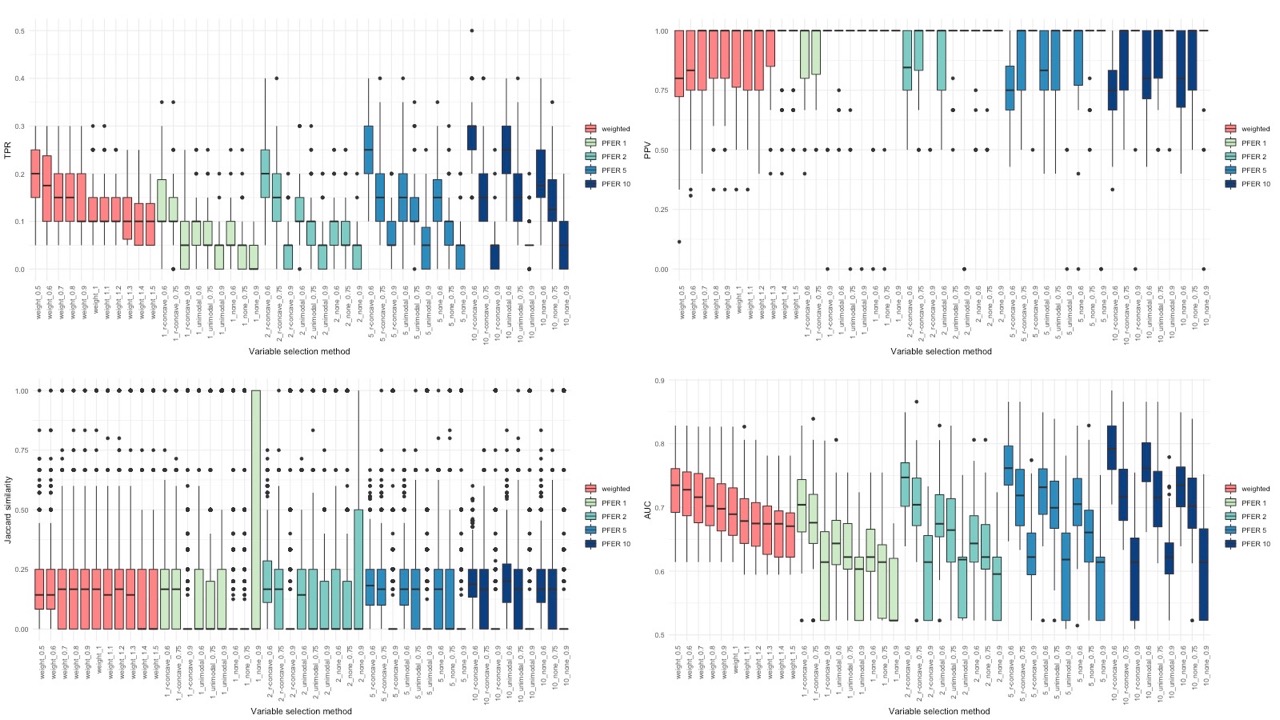


Box plots of scenario 80. $n=200, p=700, p_{signal}=20, \beta_{j}$’s of the signal variables $\sim U\left( 0.5,1.5 \right)$, event prevalence$=0.3$ and the covariance structure of $X=$independent.


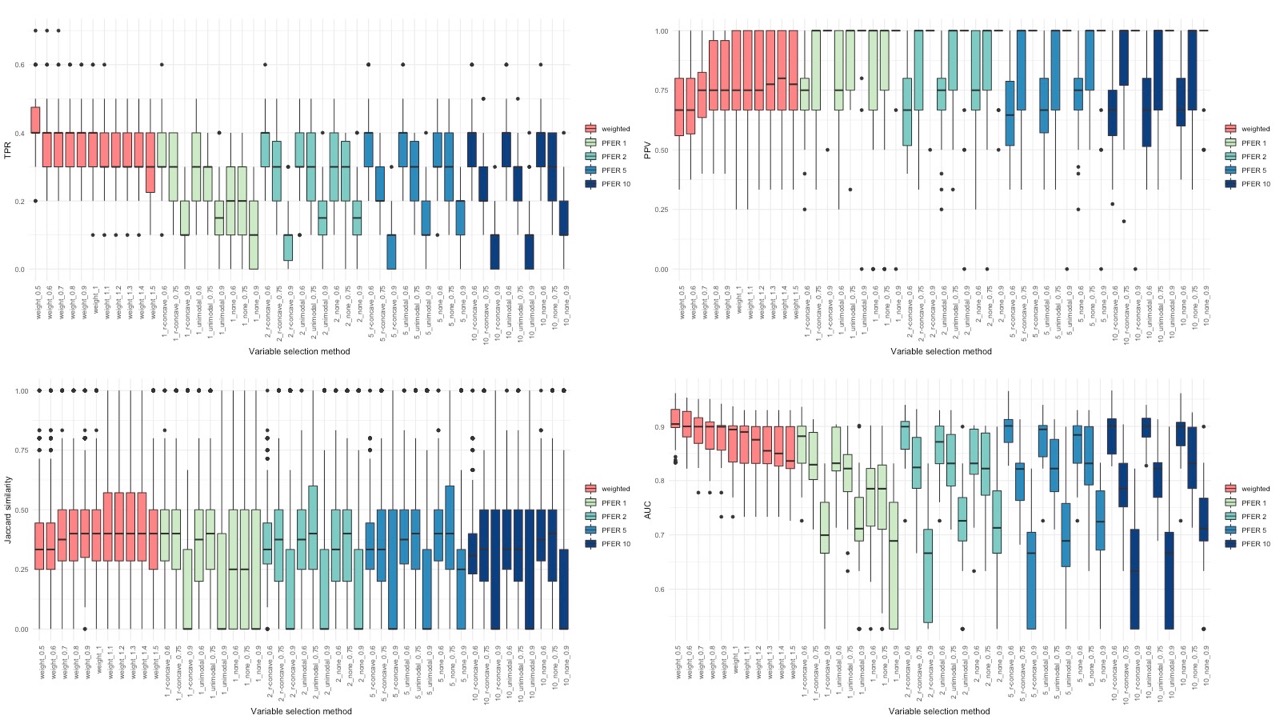


Box plots of scenario 81. $n=200, p=700, p_{signal}=10, \beta_{j}$’s of the signal variables $\sim U\left( -3,3 \right)$, event prevalence$=0.3$ and the covariance structure of $X=$Toeplitz.


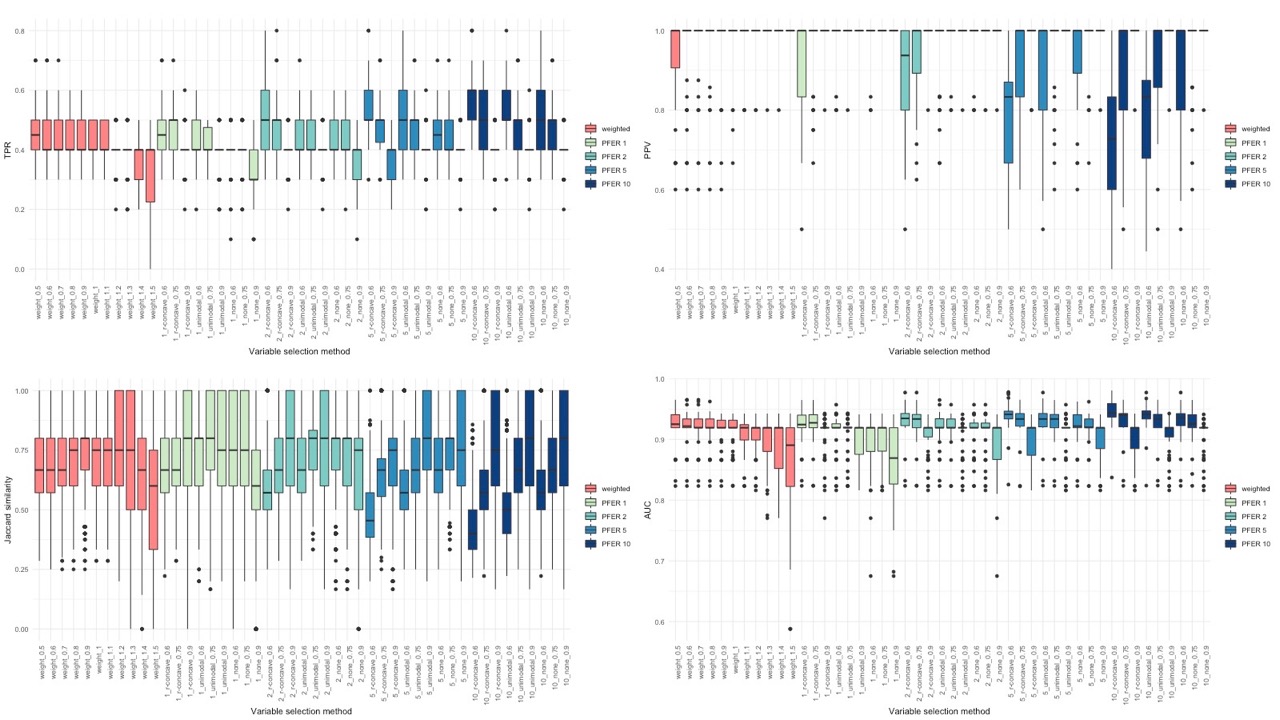


Box plots of scenario 82. $n=200, p=700, p_{signal}=10, \beta_{j}$’s of the signal variables $\sim U\left( -3,3 \right)$, event prevalence$=0.3$ and the covariance structure of $X=$independent.


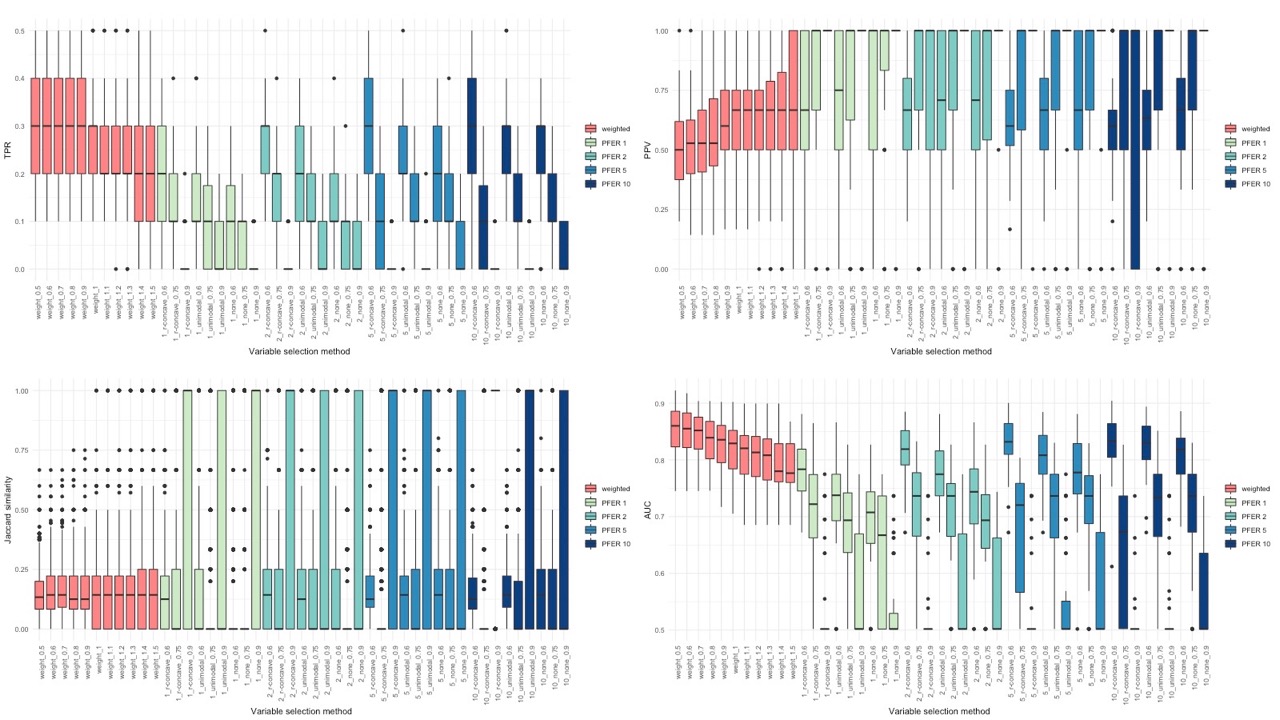


Box plots of scenario 83. $n=200, p=700, p_{signal}=10, \beta_{j}$’s of the signal variables $\sim U\left( 0.5,1.5 \right)$, event prevalence$=0.3$ and the covariance structure of $X=$Toeplitz.


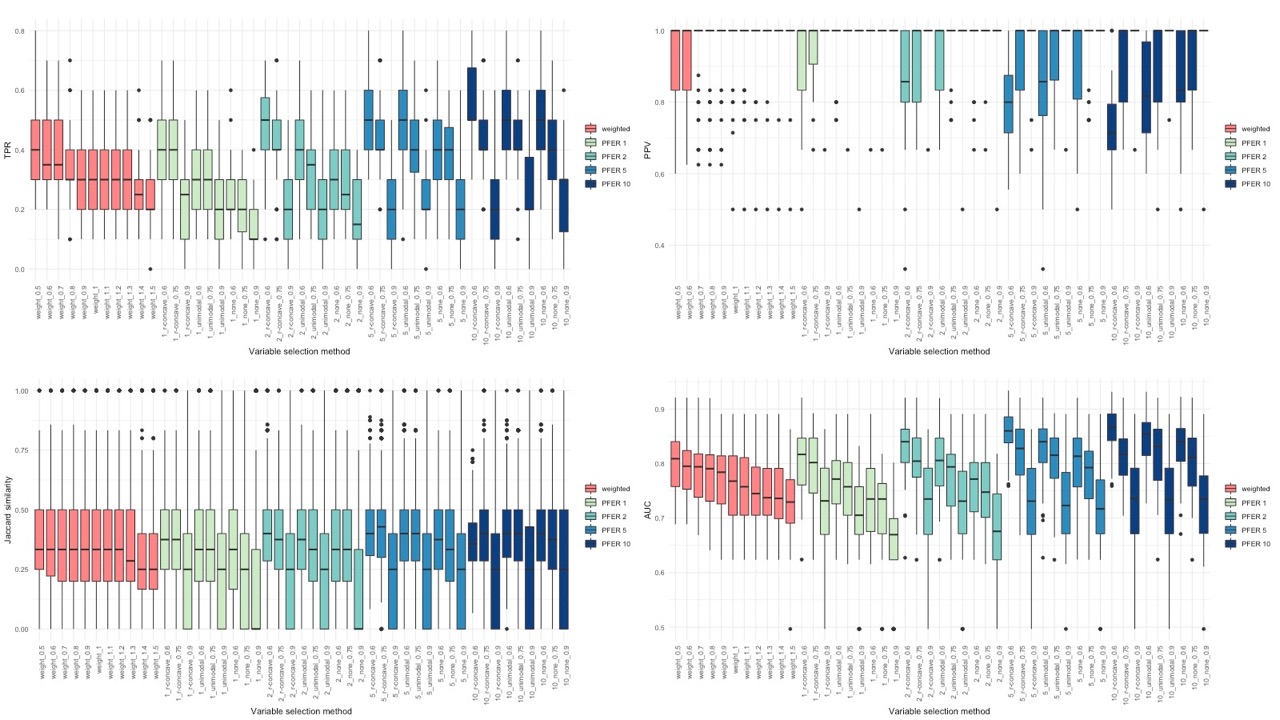


Box plots of scenario 84. $n=200, p=700, p_{signal}=10, \beta_{j}$’s of the signal variables $\sim U\left( 0.5,1.5 \right)$, event prevalence$=0.3$ and the covariance structure of $X=$independent.


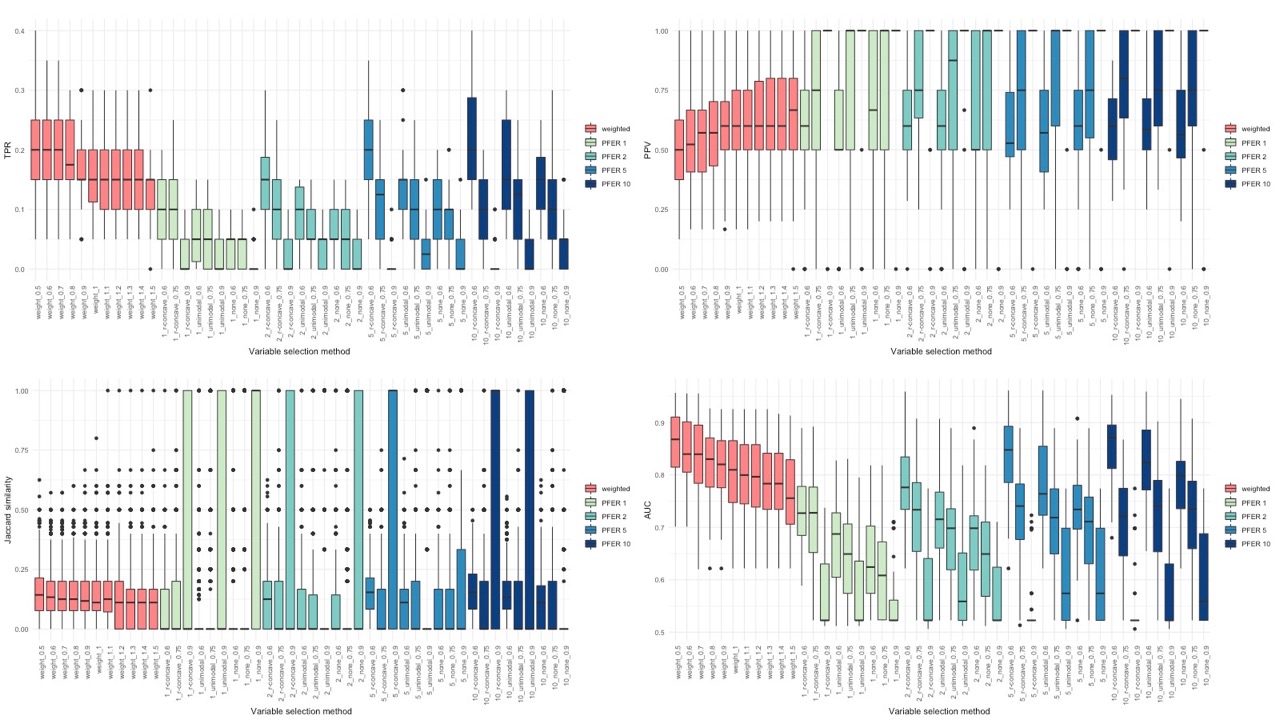


Box plots of scenario 85. $n=200, p=500, p_{signal}=20, \beta_{j}$’s of the signal variables $\sim U\left( -3,3 \right)$, event prevalence$=0.3$ and the covariance structure of $X=$Toeplitz.


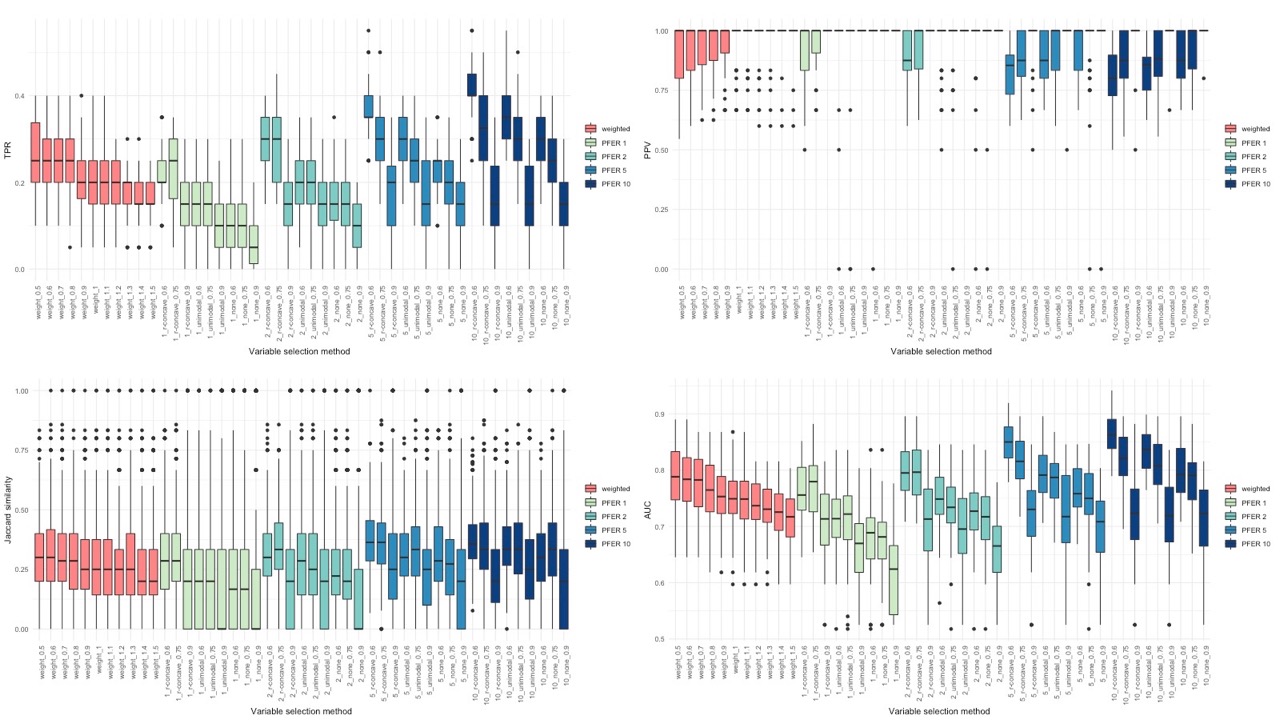


Box plots of scenario 86. $n=200, p=500, p_{signal}=20, \beta_{j}$’s of the signal variables $\sim U\left( -3,3 \right)$, event prevalence$=0.3$ and the covariance structure of $X=$independent.


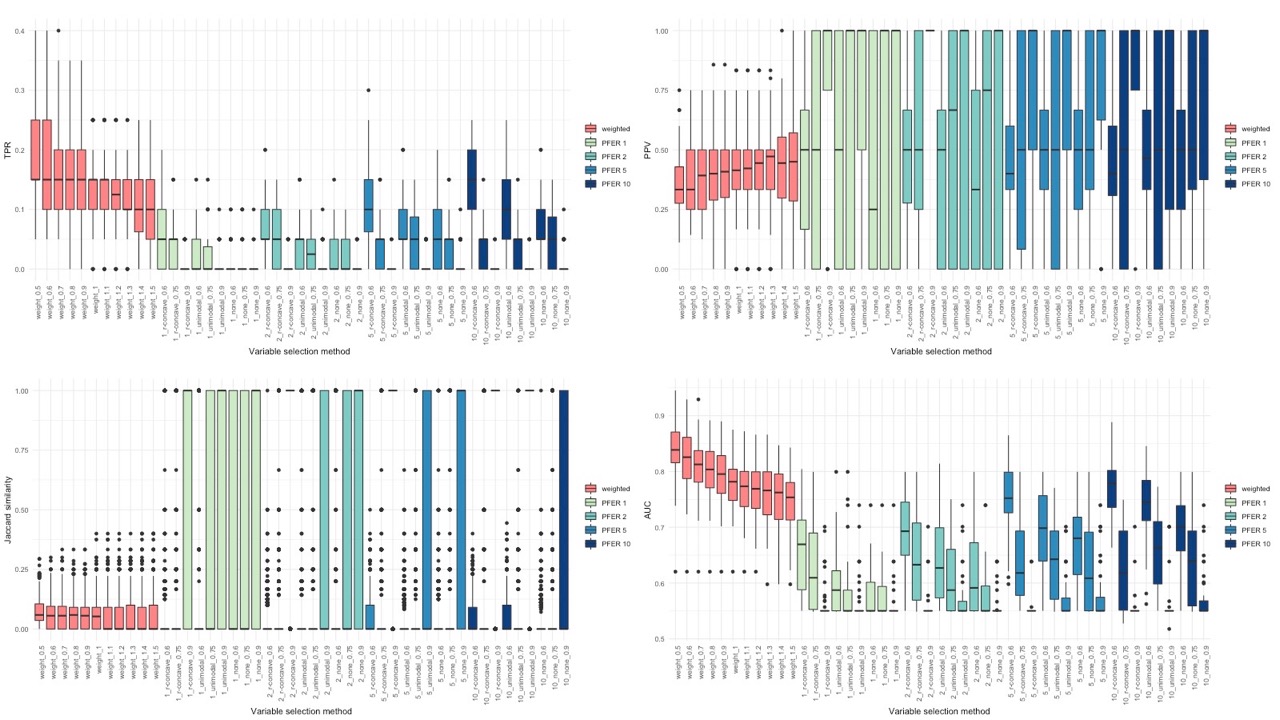


Box plots of scenario 87. $n=200, p=500, p_{signal}=20, \beta_{j}$’s of the signal variables $\sim U\left( 0.5,1.5 \right)$, event prevalence$=0.3$ and the covariance structure of $X=$Toeplitz.


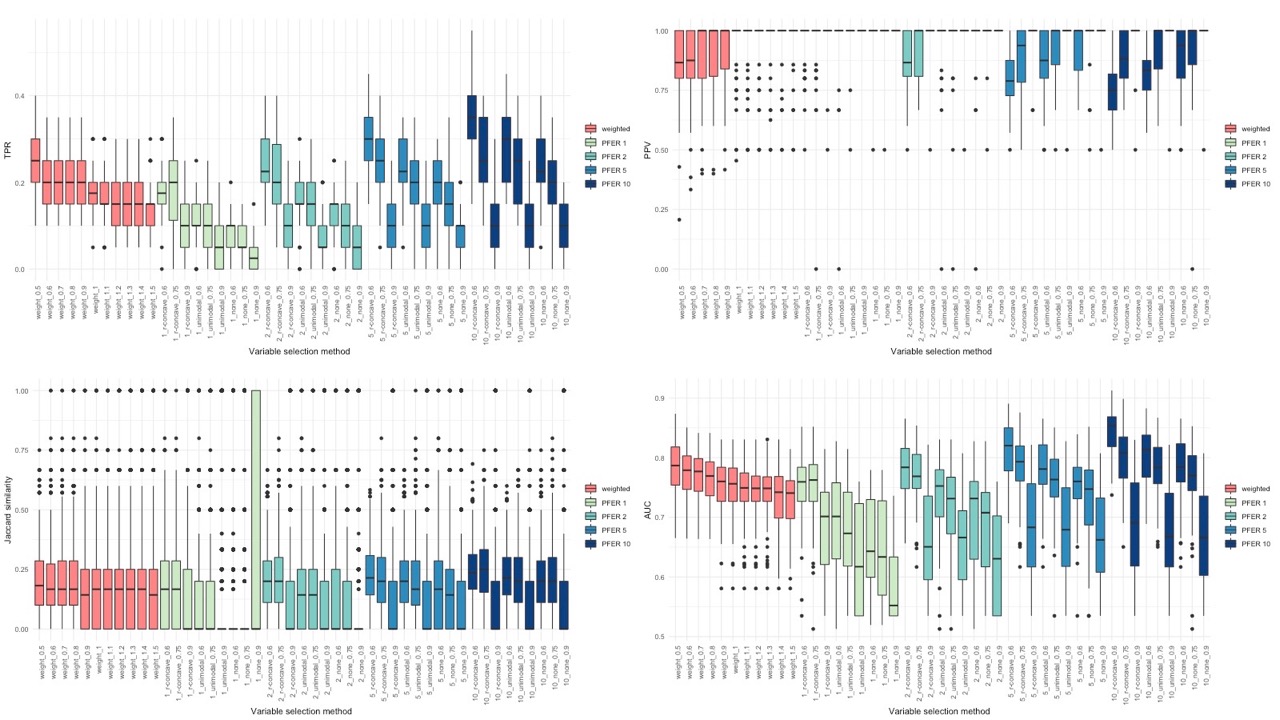


Box plots of scenario 88. $n=200, p=500, p_{signal}=20, \beta_{j}$’s of the signal variables $\sim U\left( 0.5,1.5 \right)$, event prevalence$=0.3$ and the covariance structure of $X=$independent.


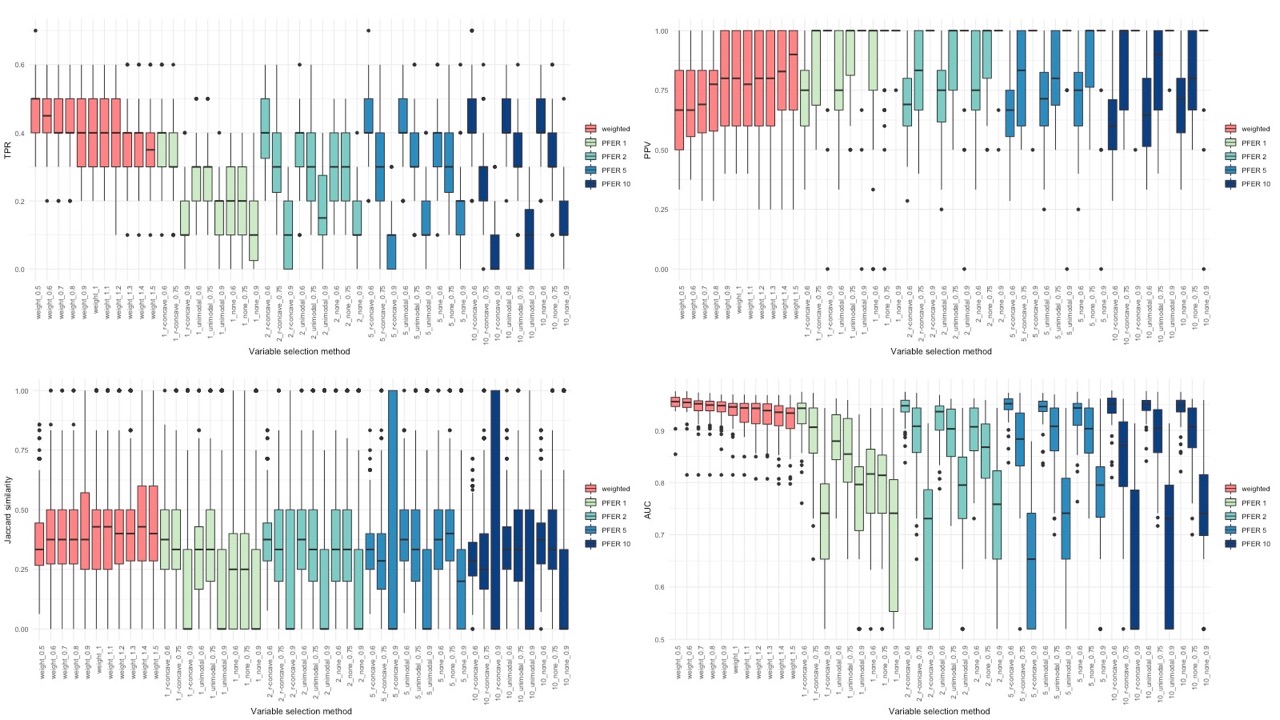


Box plots of scenario 89. $n=200, p=500, p_{signal}=10, \beta_{j}$’s of the signal variables $\sim U\left( -3,3 \right)$, event prevalence$=0.3$ and the covariance structure of $X=$Toeplitz.


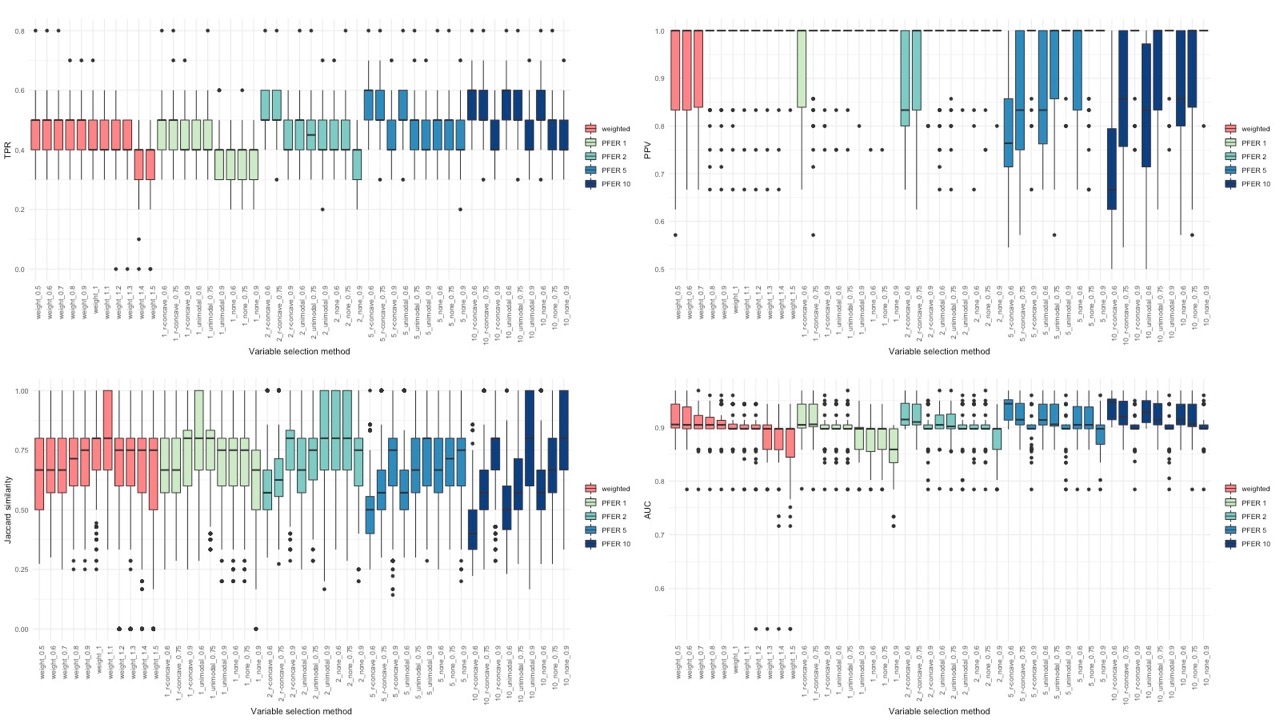


Box plots of scenario 90. $n=200, p=500, p_{signal}=10, \beta_{j}$’s of the signal variables $\sim U\left( -3,3 \right)$, event prevalence$=0.3$ and the covariance structure of $X=$independent.


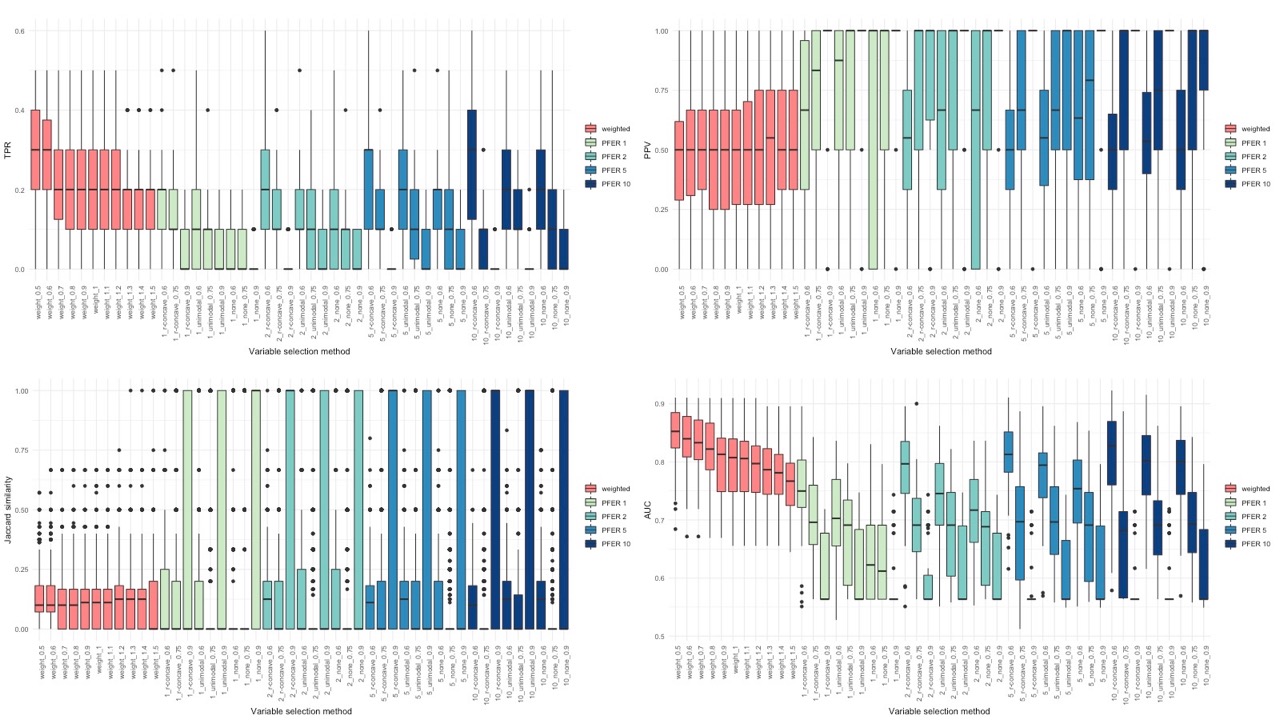


Box plots of scenario 91. $n=200, p=500, p_{signal}=10, \beta_{j}$’s of the signal variables $\sim U\left( 0.5,1.5 \right)$, event prevalence$=0.3$ and the covariance structure of $X=$Toeplitz.


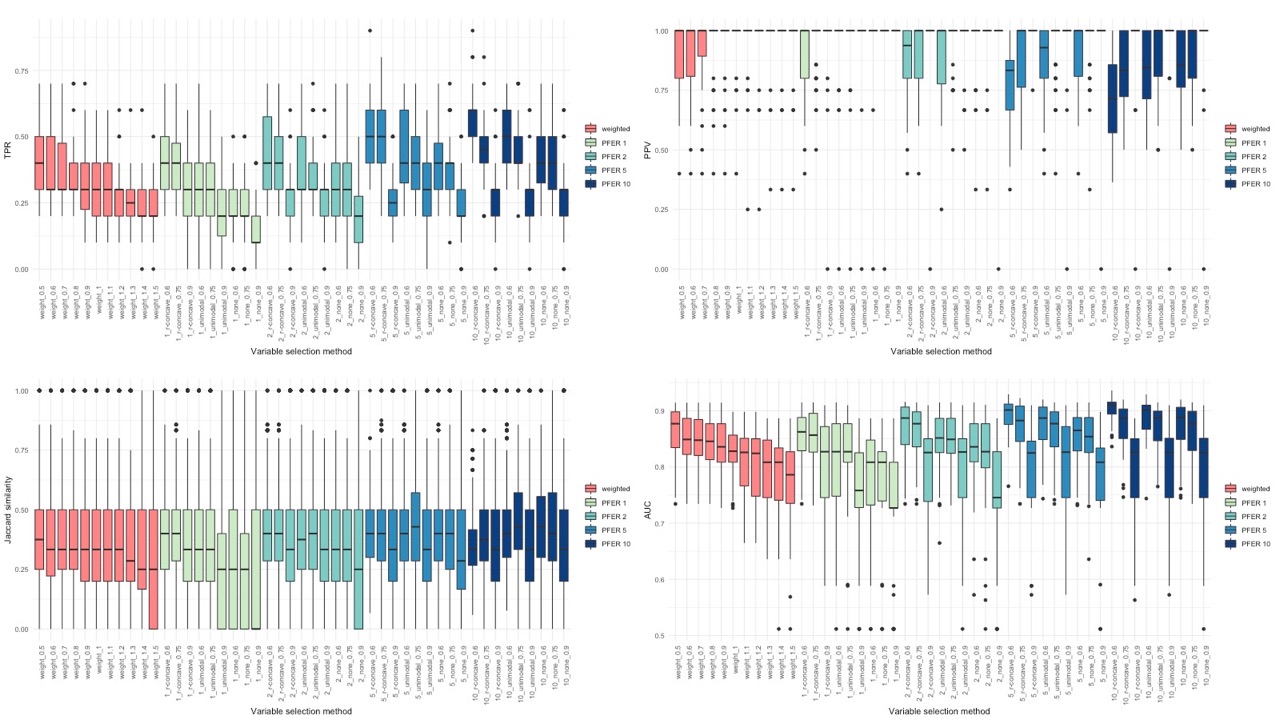


Box plots of scenario 92. $n=200, p=500, p_{signal}=10, \beta_{j}$’s of the signal variables $\sim U\left( 0.5,1.5 \right)$, event prevalence$=0.3$ and the covariance structure of $X=$independent.


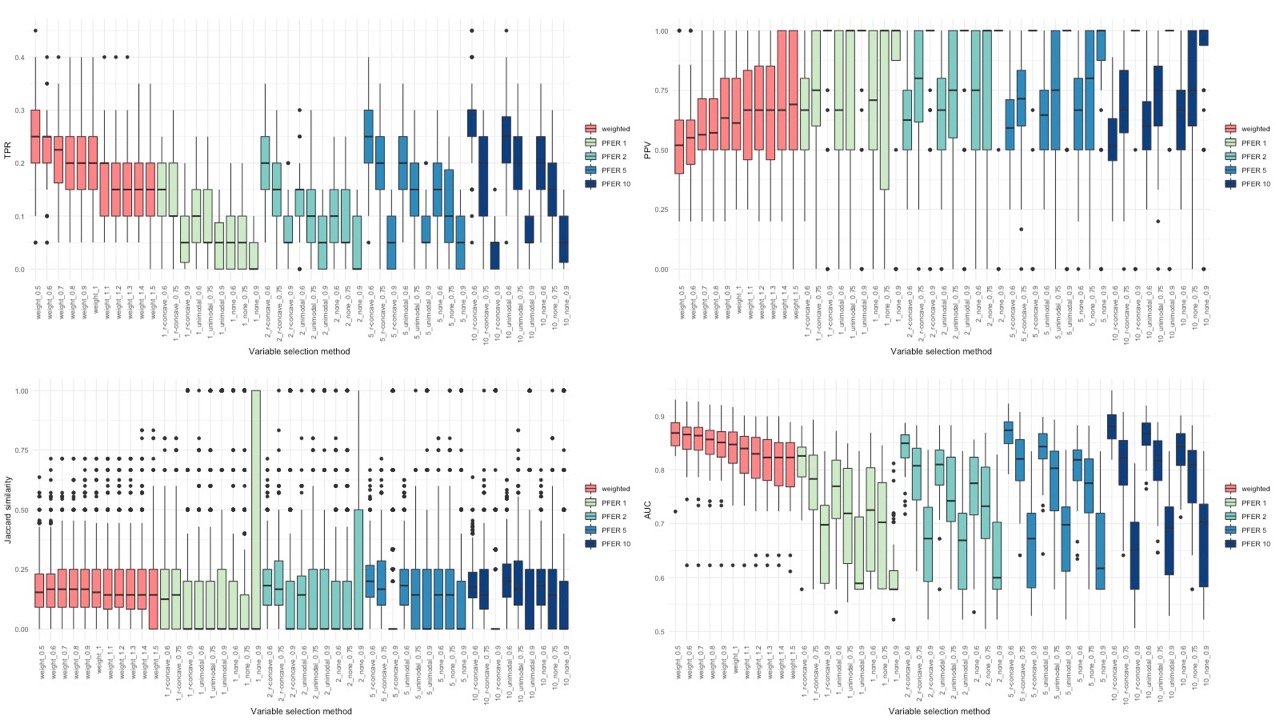


Box plots of scenario 93. $n=500, p=1000, p_{signal}=20, \beta_{j}$’s of the signal variables $\sim U\left( -3,3 \right)$, event prevalence$=0.1$ and the covariance structure of $X=$Toeplitz.


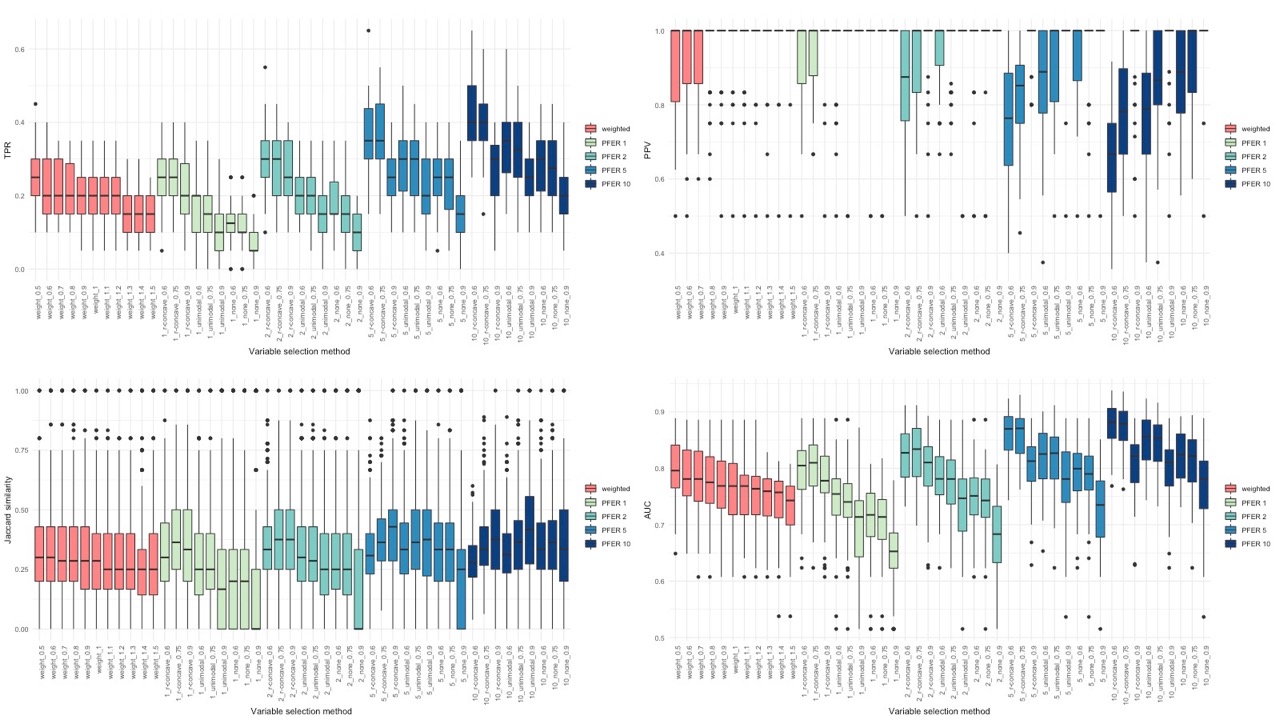


Box plots of scenario 94. $n=500, p=1000, p_{signal}=20, \beta_{j}$’s of the signal variables $\sim U\left( -3,3 \right)$, event prevalence$=0.1$ and the covariance structure of $X=$independent.


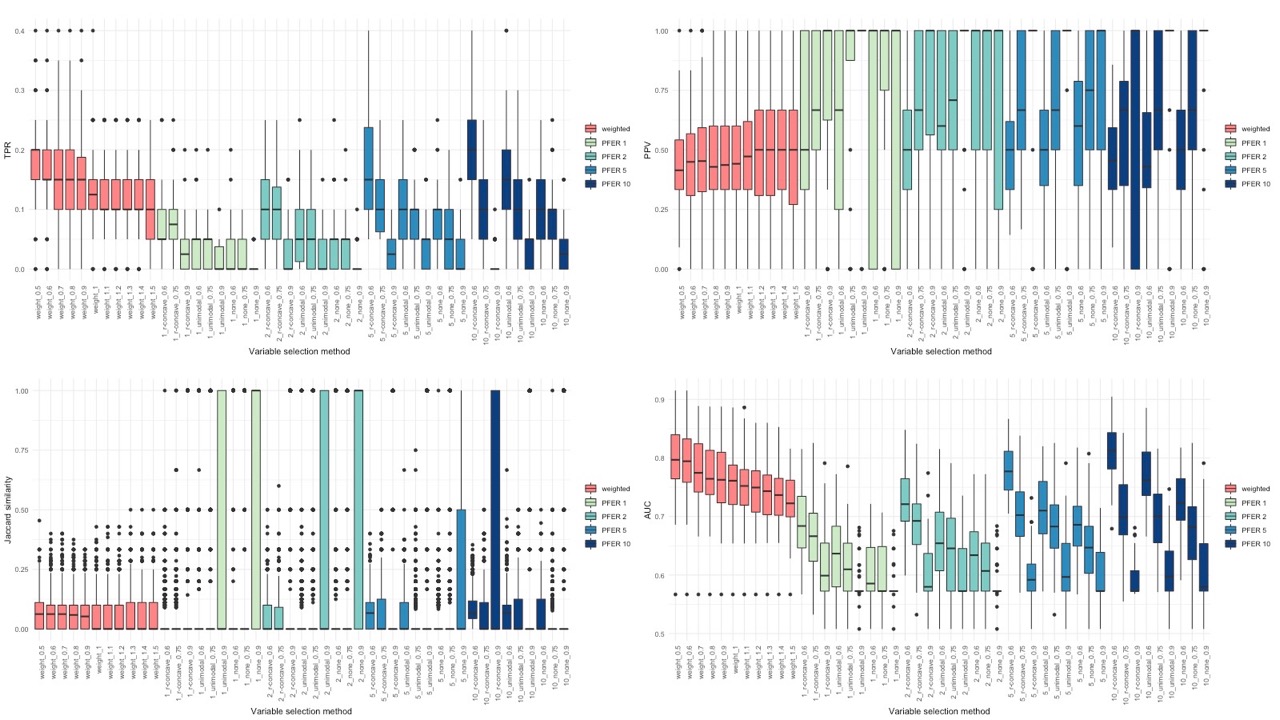


Box plots of scenario 95. $n=500, p=1000, p_{signal}=20, \beta_{j}$’s of the signal variables $\sim U\left( 0.5,1.5 \right)$, event prevalence$=0.1$ and the covariance structure of $X=$Toeplitz.


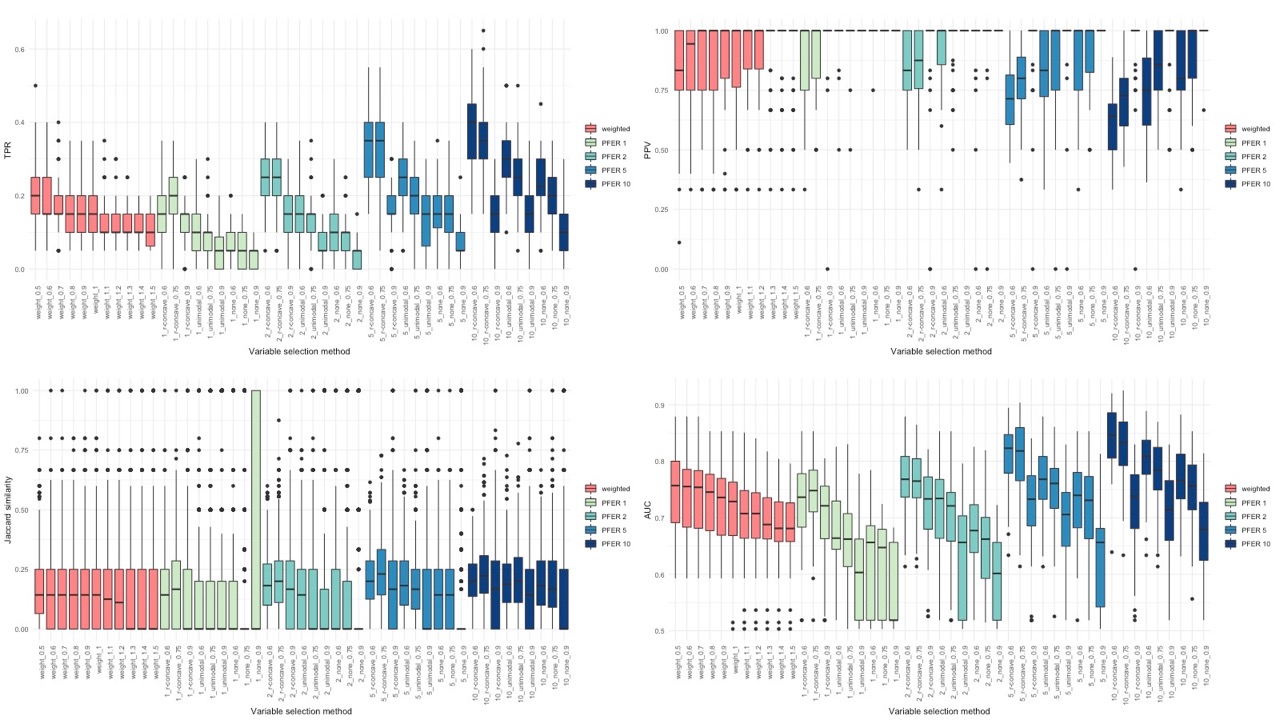


Box plots of scenario 96. $n=500, p=1000, p_{signal}=20, \beta_{j}$’s of the signal variables $\sim U\left( 0.5,1.5 \right)$, event prevalence$=0.1$ and the covariance structure of $X=$independent.


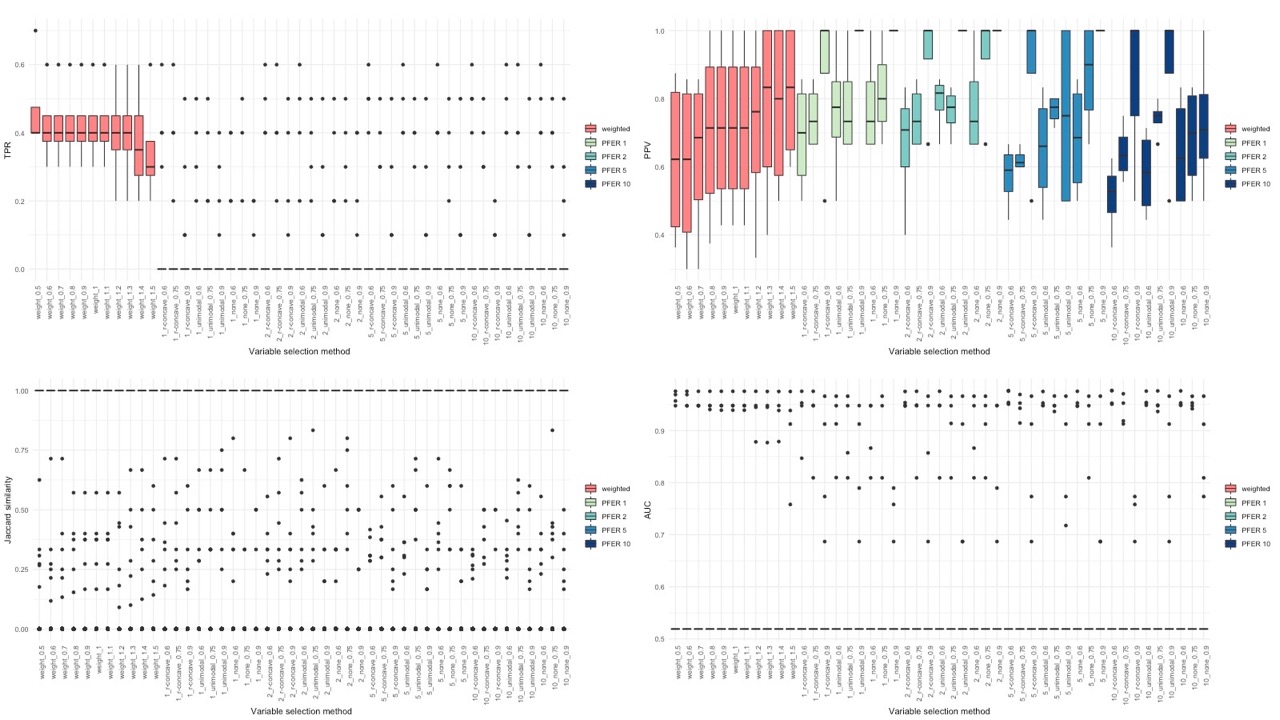


Box plots of scenario 97. $n=500, p=1000, p_{signal}=10, \beta_{j}$’s of the signal variables $\sim U\left( -3,3 \right)$, event prevalence$=0.1$ and the covariance structure of $X=$Toeplitz.


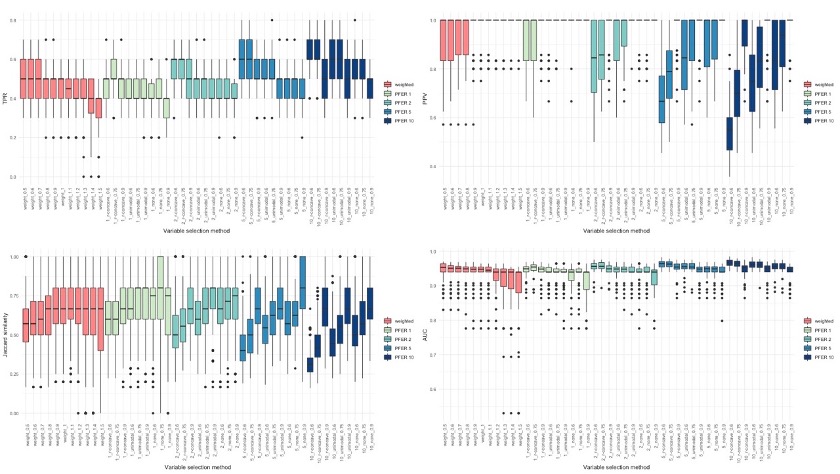


Box plots of scenario 98. $n=500, p=1000, p_{signal}=10, \beta_{j}$’s of the signal variables $\sim U\left( -3,3 \right)$, event prevalence$=0.1$ and the covariance structure of $X=$independent.


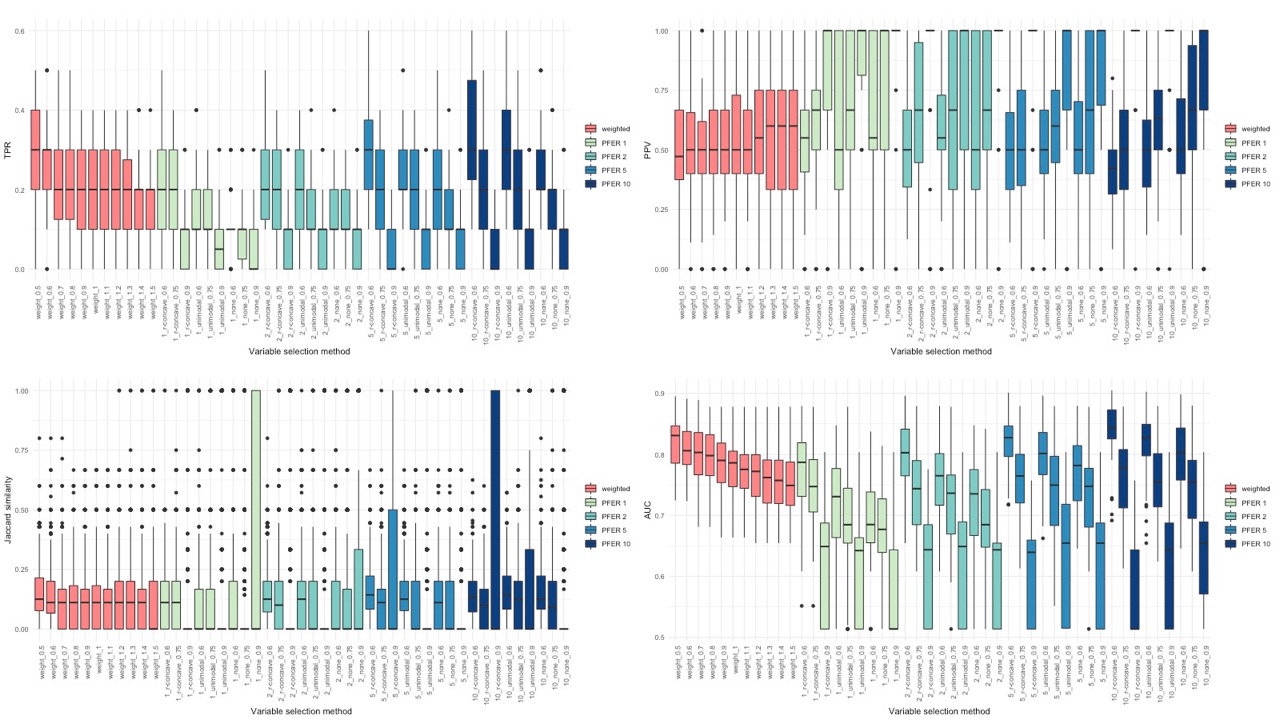


Box plots of scenario 99. $n=500, p=1000, p_{signal}=10, \beta_{j}$’s of the signal variables $\sim U\left( 0.5,1.5 \right)$, event prevalence$=0.1$ and the covariance structure of $X=$Toeplitz.


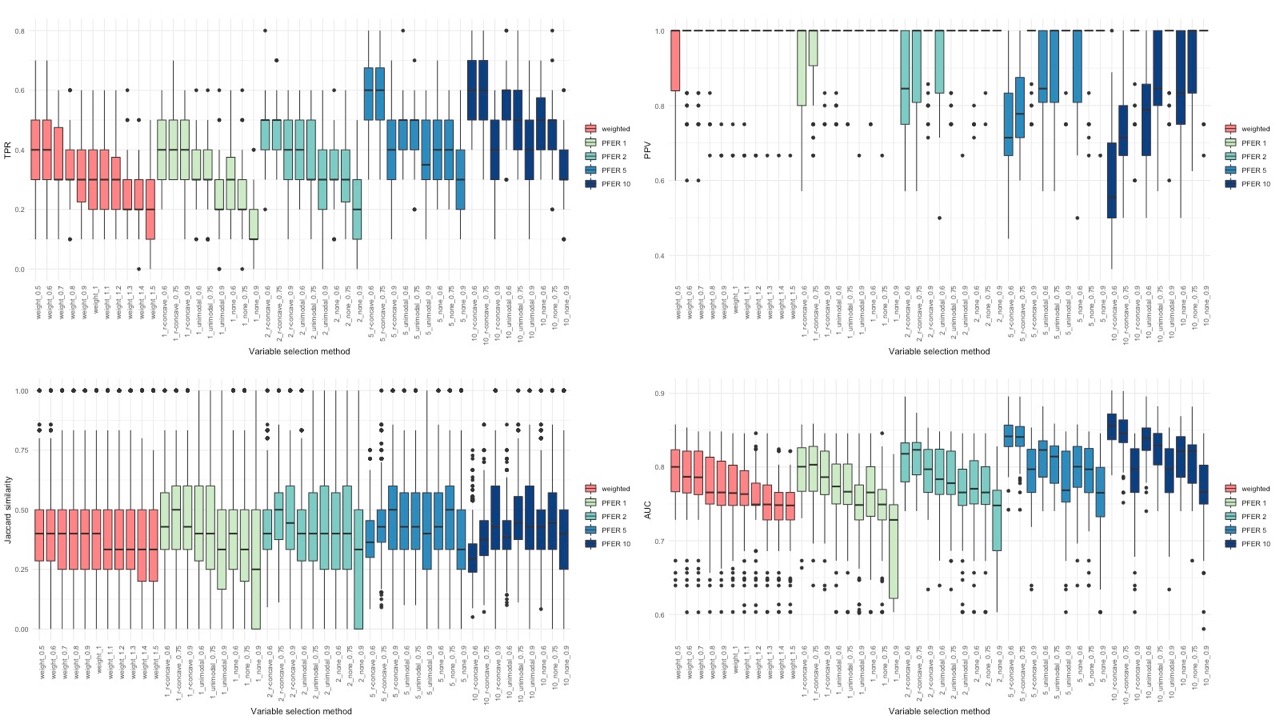


Box plots of scenario 100. $n=500, p=1000, p_{signal}=10, \beta_{j}$’s of the signal variables $\sim U\left( 0.5,1.5 \right)$, event prevalence$=0.1$ and the covariance structure of $X=$independent.

Box plots of scenario 101. $n=500, p=700, p_{signal}=20, \beta_{j}$’s of the signal variables $\sim U\left( -3,3 \right)$, event prevalence$=0.1$ and the covariance structure of $X=$Toeplitz.

Box plots of scenario 102. $n=500, p=700, p_{signal}=20, \beta_{j}$’s of the signal variables $\sim U\left( -3,3 \right)$, event prevalence$=0.1$ and the covariance structure of $X=$independent.

Box plots of scenario 103. $n=500, p=700, p_{signal}=20, \beta_{j}$’s of the signal variables $\sim U\left( 0.5,1.5 \right)$, event prevalence$=0.1$ and the covariance structure of $X=$Toeplitz.

Box plots of scenario 104. $n=500, p=700, p_{signal}=20, \beta_{j}$’s of the signal variables $\sim U\left( 0.5,1.5 \right)$, event prevalence$=0.1$ and the covariance structure of $X=$independent.

Box plots of scenario 105. $n=500, p=700, p_{signal}=10, \beta_{j}$’s of the signal variables $\sim U\left( -3,3 \right)$, event prevalence$=0.1$ and the covariance structure of $X=$Toeplitz.

Box plots of scenario 106. $n=500, p=700, p_{signal}=10, \beta_{j}$’s of the signal variables $\sim U\left( -3,3 \right)$, event prevalence$=0.1$ and the covariance structure of $X=$independent.

Box plots of scenario 107. $n=500, p=700, p_{signal}=10, \beta_{j}$’s of the signal variables $\sim U\left( 0.5,1.5 \right)$, event prevalence$=0.1$ and the covariance structure of $X=$Toeplitz.

Box plots of scenario 108. $n=500, p=700, p_{signal}=10, \beta_{j}$’s of the signal variables $\sim U\left( 0.5,1.5 \right)$, event prevalence$=0.1$ and the covariance structure of $X=$independent.

Box plots of scenario 109. $n=500, p=500, p_{signal}=20, \beta_{j}$’s of the signal variables $\sim U\left( -3,3 \right)$, event prevalence$=0.1$ and the covariance structure of $X=$Toeplitz.

Box plots of scenario 110. $n=500, p=500, p_{signal}=20, \beta_{j}$’s of the signal variables $\sim U\left( -3,3 \right)$, event prevalence$=0.1$ and the covariance structure of $X=$independent.

Box plots of scenario 111. $n=500, p=500, p_{signal}=20, \beta_{j}$’s of the signal variables $\sim U\left( 0.5,1.5 \right)$, event prevalence$=0.1$ and the covariance structure of $X=$Toeplitz.

Box plots of scenario 112. $n=500, p=500, p_{signal}=20, \beta_{j}$’s of the signal variables $\sim U\left( 0.5,1.5 \right)$, event prevalence$=0.1$ and the covariance structure of $X=$independent.

Box plots of scenario 113. $n=500, p=500, p_{signal}=10, \beta_{j}$’s of the signal variables $\sim U\left( -3,3 \right)$, event prevalence$=0.1$ and the covariance structure of $X=$Toeplitz.

Box plots of scenario 114. $n=500, p=500, p_{signal}=10, \beta_{j}$’s of the signal variables $\sim U\left( -3,3 \right)$, event prevalence$=0.1$ and the covariance structure of $X=$independent.

Box plots of scenario 115. $n=500, p=500, p_{signal}=10, \beta_{j}$’s of the signal variables $\sim U\left( 0.5,1.5 \right)$, event prevalence$=0.1$ and the covariance structure of $X=$Toeplitz.

Box plots of scenario 116. $n=500, p=500, p_{signal}=10, \beta_{j}$’s of the signal variables $\sim U\left( 0.5,1.5 \right)$, event prevalence$=0.1$ and the covariance structure of $X=$independent.
